# Citicoline Triggers Proteome Remodeling and Proteostatic Adaptation: Evidence from Shotgun Proteomics

**DOI:** 10.3390/pharmaceutics18010061

**Published:** 2026-01-01

**Authors:** Dario Cavaterra, Sara Giammaria, Irene Pandino, Gabriele Antonio Zingale, Valerio Delli Paoli, Rebecca Fiore, Manuele Michelessi, Gloria Roberti, Carmela Carnevale, Lucia Tanga, Daniela Cazzato, Elisa Peroni, Giuseppe Grasso, Gianluca Manni, Alessio Bocedi, Francesco Oddone, Massimiliano Coletta, Diego Sbardella, Grazia Raffaella Tundo

**Affiliations:** 1 Department of Chemical Sciences and Technologies, University of Tor Vergata, 00133 Rome, Italy; dario.cavaterra@uniroma2.it (D.C.); valerio.dellipaoli@students.uniroma2.eu (V.D.P.); daniela.cazzato@edu.unife.it (D.C.); alessio.bocedi@uniroma2.it (A.B.); 2 IRCCS-Fondazione Bietti, Via Livenza, 3, 00198 Rome, Italy; sara.giammaria@fondazionebietti.it (S.G.); irene.pandino@fondazionebietti.it (I.P.); gabriele.zingale@fondazionebietti.it (G.A.Z.); manuele.michelessi@fondazionebietti.it (M.M.); gloria.roberti@fondazionebietti.it (G.R.); carmela.carnevale@fondazionebietti.it (C.C.); lucia.tanga@fondazionebietti.it (L.T.); gianlucamanni53@gmail.com (G.M.); francesco.oddone@fondazionebietti.it (F.O.); massimiliano.coletta@fondazionebietti.it (M.C.); diego.sbardella@fondazionebietti.it (D.S.); 3 Department of Clinical Sciences and Translational Medicine, University of Tor Vergata, 00133 Rome, Italy; rebecca.fiore@students.uniroma2.eu; 4 CY Cergy Paris Universitè, CNRS, BioCIS UMR 8076, 95000 Cergy Pointoise, France; elisa.peroni@cyu.fr; 5 Department of Chemical Sciences, University of Catania, 95125 Catania, Italy; grassog@unict.it

**Keywords:** citicoline, glaucoma, proteomics, proteostasis, proteasome

## Abstract

**Background/Objectives**: Citicoline, also known as CDP-choline, is a nootropic agent currently used in the treatment of glaucoma and is undergoing evaluation as a first-line therapy in a multi-center, international, phase III, randomized clinical trial involving citicoline eyedrops (ClinicalTrials.gov ID: NCT05710198). Numerous clinical and preclinical studies have linked the neuroenhancement and neuroprotective effects of citicoline to its role as a metabolic precursor for structural and functional components of cell membranes (such as phosphatidylcholine and sphingomyelin) and for neurotransmitters (e.g., acetylcholine and dopamine). However, compelling evidence suggests that the molecular mechanisms underlying its cytoprotective activity involve additional as-yet uncharacterized pharmacological actions. **Methods**: To further elucidate its pharmacology, we investigated the effect of two cytoprotective doses of citicoline (0.1 mM and 1 mM) on the global proteome of neuroblastoma cells using an unbiased shotgun proteomics approach. **Results**: With over 4000 unique proteins identified and quantified per experimental condition, the proteomics analysis revealed that citicoline, after 6 h of stimulation, induces a profound and robust remodeling of the intracellular proteome compared to untreated cells. Importantly, this effect was observed to significantly diminish by 18 h of stimulation, highlighting its transient nature (data are available via ProteomeXchange with identifier PXD061053). The clustering and rationalization of proteins upregulated by citicoline treatment identified the enrichment of key pathways for mRNA splicing, protein translation, proteostasis balance through the ubiquitin proteasome system (UPS), and mitochondrial metabolism. **Conclusions**: These proteomics findings introduce previously uncharacterized biological effects of citicoline and foster the working hypothesis that this drug may exert its cytoprotective activity through molecular mechanisms linked to the hormesis principle. These data further support the rationale for its clinical application in neurodegenerative processes and human disorders characterized by proteotoxicity.

## 1. Introduction

Citicoline, also known as CDP-choline, is a nootropic agent employed in the therapy of glaucoma, a family of chronic optic neuropathies characterized by degeneration of the Retinal Ganglion Cells (RGCs) and the optic nerve [1,2,3]. Open-Angle Glaucoma (OAG), which is the prevalent form of the disease, is the second leading cause of irreversible blindness on a global scale [4,5,6].

Citicoline is currently utilized as adjunctive treatment for glaucoma to hypotensive therapies that lower the intraocular pressure (IOP). Indeed, IOP increase (>21 mmHg) is the most relevant risk factor of glaucoma and the only one modifiable by surgical or pharmacological approaches [4,7]. 

Over the last two decades, several studies with citicoline oral solution and eye drops have demonstrated its ability to slow down disease progression in patients with glaucoma [8,9,10,11,12]. A multi-center, international, phase III, randomized clinical trial with citicoline eyedrops is currently ongoing (ClinicalTrials.gov ID NCT05710198) for the treatment of glaucoma as a first-line therapy. The primary outcome of this trial is the difference in the rate of progression of visual field (VF) damage in the study eye between the two trial arms.

In addition to glaucoma, previous studies have also reported citicoline’s efficacy for the treatment of additional brain morbidities such as stroke and intracerebral hemorrhage [7,13,14,15,16].

Despite its diffuse use and experimentation for different diseases, the pharmacological properties and biological activities of citicoline are not fully uncharacterized yet.

However, there is compelling evidence that the molecular mechanism through which citicoline exerts the neuroenhancement and neuroprotective activities are multifarious. Citicoline, as endogenous metabolite, serves as a synthetic precursor for essential structural and functional components of the cell membrane, such as phosphatidylcholine and sphingomyelin, and for the neurotransmitters dopamine and acetylcholine [3,17]. Therefore, upon intake, citicoline and its metabolites are presumed to fuel biosynthetic pathways that stimulate neuron homeostasis, synaptic transmission, and plasticity, thereby promoting cognitive and visual performance [3,17].

In addition to these neuroprotective and neuroenhancement effects, several independent in vitro and in vivo studies have suggested that citicoline may possess additional and unexplored cytoprotective activities that further support its clinical efficacy [18,19,20,21,22,23].

In this context, in a previous study, we reported that citicoline acts as an allosteric modulator of the 20S proteasome [24]. The proteasome is the multi-enzymatic complex of the Ubiquitin–Proteasome System (UPS), which constitutes the main intracellular proteolytic system [25,26,27]. In our study, citicoline was found to promote the clearance of synthetic and macromolecular substrates, such as α-synuclein in vitro, and the turnover of proteasomal substrates in a neuroblastoma cell line [24].

In this regard, the UPS, along with several additional protein families (e.g., Heat Shock Proteins), constitutes a first-line defense of the cell against proteotoxic insults. Given the critical importance of proteostasis balance, especially for post-mitotic cells, eukaryotes have evolved multiple mechanisms to potentiate these protective systems depending on metabolic demands. A paradigmatic example is hormesis, which defines the process of proteome adaptation and potentiation in response to noxious stimuli, such as chemicals, physical, and biological stressors (including those against which citicoline was tested in vitro) [28,29,30]. A hormetic response is driven by increased production of cytoprotective and restorative proteins, including growth factors, phase II antioxidant enzymes, UPS components, endoplasmic reticulum (ER) stress proteins, and chaperones [28,29,30]. Thus, a deeper understanding of molecular hormesis mechanisms is leading to novel approaches for the prevention and treatment of many diseases, such as neurodegeneration [28,29,30].

To elucidate previously unidentified mechanisms governing citicoline’s neuroprotective and neuroenhancement properties, we here conducted an unbiased shotgun mass-spectrometry analysis of the global proteome in neuroblastoma SH-SY5Y cells. To this aim, cells were treated with citicoline doses previously linked to neuroprotection in various preclinical models, including this cell strain, for 6 h and 18 h [22,23,24]. Citicoline administration induced a transient yet robust remodeling of the intracellular proteome after 6 h of treatment. This comprehensive approach allowed us to identify the key cellular processes that undergo dynamic changes, including those related to protein turnover and proteostasis regulation, mitochondrial metabolism, transcriptional/translational processes, and RNA splicing casting light on novel and heterogeneous mechanisms through which citicoline may exert its reported neuroprotective activities.

## 2. Materials and Methods

### 2.1. Cell Cultivation, Citicoline Stimulation, Cytotoxicity Assay

SH-SY5Y cells (ATCC) were cultivated in High-Glucose Dulbecco Modified Eagle’s Medium (DMEM) supplemented with 10% Fetal Bovine Serum (FBS), MEM non-essential amino acids, and antibiotics (Penicillin and Streptomycin) under humidified atmosphere (5% CO_2_, 37 °C).

For citicoline stimulation, cells were seeded at a concentration of 6 × 10^5^ cells/well (for 6 h of stimulation) and at a concentration of 5 × 10^5^ (for 18 h of stimulation) in a 6-well Transwell plate in complete medium and allowed to adhere after an over-night incubation.

Citicoline (sodium salt, >99% purity, supplied by Omikron Italia Srl, Roma, Italy), was dissolved fresh before use in prewarmed (at 37 °C) complete DMEM and delivered at the indicated concentration (0.1 mM and 1 mM) in the culture medium. In parallel, cells were left untreated.

At the indicated incubation time (i.e., 6 h and 18 h), cell layers were gently washed with pre-chilled phosphate-buffered saline (PBS) 1x, detached by scraping, centrifuged (1500 rpm, 10 min, 4 °C) to remove cell debris, and the cell pellets stored at −80 °C until use.

Three biological replicates (n = 3), each analyzed twice, were used for every experimental condition in the shotgun proteomics study.

Before undertaking the proteomics workflow, the citicoline doses here tested were assayed for unwanted and confounding cytotoxic effects, although they were already proven to be safe and protective.

To this aim, a dimethylthiazol-carboxymethoxyphenyl-sulfophenyl-tetrazolium (MTS) (Abcam Limited, Cambridge, UK) assay was set up.

Briefly, 1.5 × 10^4^ SH-SY5Y cells were seeded in a 96-well Transwell cell plate and stimulated with a range of citicoline concentration spanning from 0.1 mM to 1 mM for 24 h. Citicoline solution was prepared freshly as above indicated. The day after stimulation, the MTS solution was added to the culture medium (20 μL/well), and after 4 h of incubation, optical densities (O.D.) were recorded at 490 nm into a Varioskan Lux spectrophotometer (Thermo Fisher Scientific, Waltham, MA, USA). Differences in cell count were analyzed using the non-parametric Kruskal–Wallis test in GraphPad Prism (v8.0).

### 2.2. Sample Preparation for Mass Spectrometry Studies

For shotgun proteomics, cell pellets were lysed in pre-chilled urea denaturing buffer (8 M urea, 50 mM Tris-HCl, 150 mM NaCl, pH 8) supplemented with proteases inhibitors cocktail, PMSF, and phosphatases inhibitors (sodium orthovanadate and β-glycerophosphate, both 1 mM) (Merck-Millipore, Darmstadt, Germany), sonicated (3 bursts for 10 s each, at 30 Hz), and cleared using centrifugation (13.000 rpm, 10 min, at 4 °C).

Protein concentration was determined using a BCA protein assay (Thermo Fisher Scientific, MA, USA), and 100 µg was enrolled for trypsin digestion. First, proteins were denatured with 5 mM dithiothreitol (DTT) (45 min, at room temperature [r.t.]), alkylated with 10 mM iodoacetamide (30 min in the dark, r.t.). Then, samples were diluted to reduce the urea concentration to 1 M and digested with mass-grade lysil C-endopeptidase (Wako Chemicals, Osaka, Japan) (1:200 enzyme–protein ratio, 2 h, r.t.) and mass-grade trypsin (Fisher Scientific, Waltham, MA, USA) (1:25 overnight, r.t.). The reaction was quenched with 0.4% trifluoroacetic acid (TFA) and peptides were desalted and cleaned using C18 stage-tips (Fisher Scientific, Waltham, MA, USA) by following the manufacturer’s instructions. Peptides were quantified by BCA peptide assay to inject equal quantities for each sample, dried in a Speed Vacuum concentrator (Labconco, Kansas City, MO, USA) and resuspended in 5% acetonitrile (ACN), 0.1% formic acid (FA).

### 2.3. Mass Spectrometry Parameters

Proteomic analysis was performed injecting 1 µg peptides for each experimental conditions into an Orbitrap Exploris 240 mass spectrometer coupled to an Ultimate 3000 nano-ultra high-performance liquid chromatography (nano-UHPLC) system (Thermo Fisher Scientific, MA, USA). Gradient was set as it follows: Solvent A: 100% H_2_O, 0.1% Formic Acid; Solvent B: 80% Acetonitrile, 0.1% Formic Acid. UHPLC Gradient (minutes–%B): 0–6.7; 2–6.7; 62–34.4; 67–55.5; 72–100; 80–100; 82–6.7; 88–6.7. Column oven temperature: 45 °C. Run time: 88 min. Loading Pump flowrate: 30 μL/min. NC Pump flowrate: 250 nL/min. Data acquisition was conducted in Data Dependent Acquisition (DDA) mode.

Orbitrap Resolution: 120,000; Scan Range (*m*/*z*): 375–1650; RF Lens (%): 80; Normalized AGC Target (%): 300. ddMS^2^ was triggered using the following filters: Isolation Window (*m*/*z*): 2; Normalized HCD Collision Energy (%): 30; Orbitrap Resolution: 15,000; Normalized AGC Target (%): 50.

### 2.4. Raw Data Processing and Statistical Analysis

Protein and peptides were searched using Proteome Discoverer (PD) software (v. 2.5, Thermo Fisher Scientific, Waltham, MA, USA) against a UniProt human protein FASTA database including protein isoforms. Sequest implemented with the Inferys rescoring algorithm was used and a concatenated target-decoy strategy applied for the determination of the proteins’ False Discovery Rate (strict FDR ≤ 0.01 and relaxed FDR ≤ 0.05). A contaminant database was included in the analysis. 

Trypsin (full) was set as enzyme, 10 ppm precursor mass tolerance, and 0.02 Da fragment mass tolerance. Carbamidomethylation of cysteines (+57.021) was set as static modification, whereas oxidation on methionine (+15.995) was set as dynamic modification per peptides according to the requirements of Inferys algorithm.

The resulting protein and peptide identifications and quantitation data were analyzed using in-house-built scripts written in R Studio (v 4.5.1) using the following libraries: ggplot, ggvenn, ggcorrplot, FactormMineR, and factoextra.

Quality metrics for proteomics data were individually assessed for all experimental replicates, as presented in relevant figures.

To identify differentially expressed proteins (DEPs) between the tested comparisons, an empirical Bayes moderated τ test, commonly known as Linear Models for Microarray Data (Limma), using limma R package was employed [31,32]. This method enhances robustness for studies with relatively low sample sizes by borrowing variance information across the different tests on individual proteins. False Discovery Rate (FDR) control was applied using both Storey’s q-value method (using the qvalue R package) and the Benjamini–Hochberg (BH) procedure, which is default implemented in limma package; both methods yielded consistent results.

Gene Ontology (GO) enrichment and Kyoto Encyclopedia of Genes and Genomes (KEGG) pathway analyses were performed within the R environment using the clusterProfiler package, leveraging the org.Hs.eg.db database for human annotations [33,34]. To focus discussion on main relevant terms and pathway, a more conservative filter (FDR ≤ 0.01) was applied for data filtering.

Protein–protein interaction (PPI) network analysis was performed using the STRING Network software (version 22.0) (https://string-db.org/) [35]. Networks were constructed with a high confidence threshold, setting the minimum interaction score at 0.9. To identify distinct, tightly connected groups, these networks were then filtered using the k-nearest neighbors (knn) procedure with n = 20. This unsupervised machine learning algorithm groups data points (proteins in this case) based on their proximity to the network, effectively identifying local neighborhoods or communities.

### 2.5. Western Blotting Studies

For Western blotting studies, the same cell lysates (urea buffer) enrolled in proteomics analysis were used. In all cases, 5 µg proteins were heat-denatured and reduced in Laemmli buffer 1× supplemented with 1 mM DTT. Thereafter, 4–20% acrylamide pre-cast gels (Bio-Rad, Hercules, CA, USA) were used to separate proteins by SDS-PAGE. After separation, proteins were transferred to a HyBond-ECL nitrocellulose filters (Bio-Rad, Hercules, CA, USA) and probed with indicated antibodies. All antibodies used were purchased from ProteinTech (Rosemont, IL, USA). Antibodies were diluted 1:3000 in 0.1% Tween-PBS plus 0.1% fat-free milk and with a horseradish peroxidase-conjugated anti-rabbit or anti-mouse IgG antibody (Bio-Rad, Hercules, CA, USA), diluted 1:10,000 in 0.1% Tween-PBS plus 0.1% fat-free milk.

Proteins were developed using ECL chemiluminescence and recorded using an iBright 1500 (ThermoFisher scientific, Waltham, MA, USA).

Protein intensities were normalized on total protein staining by Ponceau S according to recent guidelines and to the calculation of the linear dynamic range of internal controls (e.g., β-actin, tubulin) previously calculated in our laboratory [36,37].

Statistical differences across the experimental groups were analyzed using the Kruskal–Wallis test followed by Dunn’s test for multiple comparisons between groups. Significance threshold was set for *p* ≤ 0.05. Analysis of Western blotting data was performed using GraphPad Prism software (v8.0).

Uncropped filters used for Wb panel are shown in Supplementary Figure S6.

## 3. Results

### 3.1. Study Outline

To enhance our current knowledge regarding citicoline pharmacology and to elucidate the molecular mechanisms underpinning its neuroprotective effects, we investigated whether administration of this nootropic agent perturbs the intracellular proteome of a cultured cell line. To this aim, SH-SY5Y cells were stimulated with two citicoline concentrations, 0.1 mM and 1 mM, for 6 h and 18 h. These tested doses have previously demonstrated neuroenhancement and neuroprotective activities against various noxious stimuli across different experimental models, including the cell strain utilized in this study [22,23,24].

First, potential cytotoxic effects were ruled out. A 24 h MTS assay confirmed that 0.1 mM and 1 mM citicoline did not induce adverse effects on cell proliferation and viability (Supplementary Figure S1). These findings, consistent with previous studies, showed no impact of the tested citicoline doses on cell growth and viability [22,23,24].

**Figure 1:**
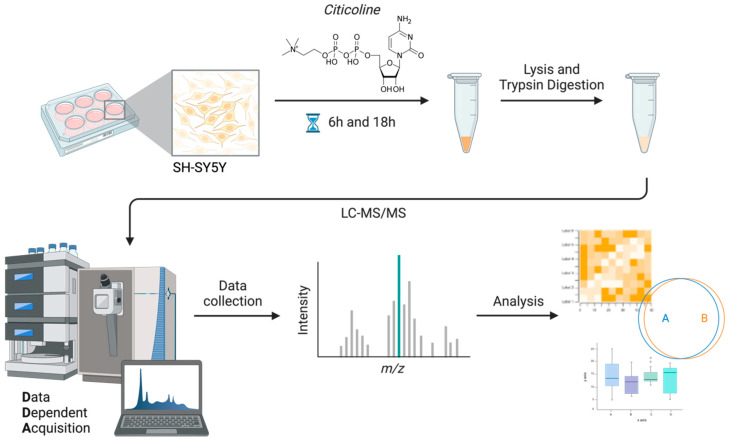
Schematic representation of the shotgun proteomic study workflow.

Subsequently, SH-SY5Y cells were cultured and stimulated with citicoline according to the workflow summarized in Figure 1. The global proteome of SH-SY5Y cells was then explored using a shotgun label-free quantification (LFQ) approach, with spectra acquired via DDA (Data-Dependent Acquisition) modality. All .raw files were submitted to Proteome Discoverer (PD) v2.5 and spectra were searched against a human FASTA database using Sequest supplemented with the Inferys rescoring algorithm and setting FDR ≤ 0.01 for PSM validation by a concatenated target-decoy strategy. The proteins included in the downstream analysis were preliminarily filtered for Master proteins (PD glossary) identified with high confidence (FDR ≤ 0.01, with ≥2 peptides in almost all cases).

### 3.2. Shotgun Proteomics Analysis Identified > 4000 Unique Proteins per Experimental Condition

The high-throughput datasets were first assessed for technical soundness. Initial analysis involved filtering out contaminants, which were found to be negligible. Upon log_2_ transformation (Supplementary Figure S2A), median raw intensities (i.e., prior to data normalization) displayed a comparable pattern across biological replicates (intensities of two replicates for each experimental point were first averaged). Furthermore, the density plot of the log_2_-transformed data clearly indicated a comparable and substantially overlapping distribution across all samples included in the study, suggesting high technical consistency between replicates and experimental groups (Supplementary Figure S2B). Subsequently, to further verify data reproducibility across biological replicates, the Pearson coefficient of correlation was computed and visualized via a correlogram (Supplementary Figure S2C). The resulting data highlighted a strong degree of correlation among replicates.

**Figure 2:**
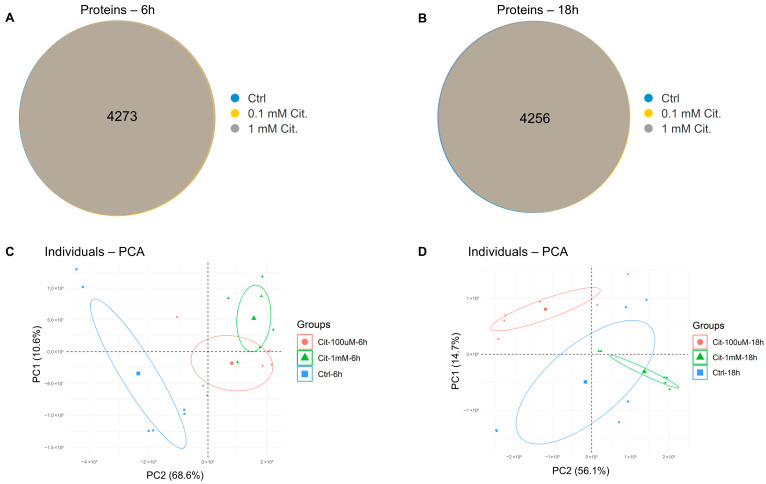
(**A**) Venn diagram of proteins identified (master proteins with FDR ≤ 0.01) in each experimental group relative to 6 h of citicoline stimulation; (**B**) Venn diagram of proteins identified (master proteins with FDR ≤ 0.01) in each experimental group relative to 18 h of citicoline stimulation; (**C**) PCA of proteins identified in each experimental group relative to 6 h of citicoline stimulation. The centroid position is reported; (**D**) PCA of proteins identified in each experimental group relative to 18 h of citicoline stimulation. The centroid position is reported.

In total, 4273 unique proteins were identified and quantified across all experimental groups exposed to 6 h of citicoline stimulation (Figure 2A) and 4256 proteins for 18 h of stimulation (Figure 2B). Of these, 3800 proteins were consistently quantified, exhibiting no missing values in any replicate across all experimental groups.

Based on all these technical data, a quantile normalization (QN) procedure was deemed applicable.

After normalization, imputation of missing values was performed using the CART method.

To investigate the global differences between experimental groups while reducing data dimensionality, a principal component analysis (PCA) was computed separately for the 6 h (Figure 2C) and 18 h (Figure 2D) time points. The distinct separation of data along PC1 and PC2, coupled with the position of the experimental group-specific centroids, strongly suggests that the proteomes of citicoline-stimulated cells possessed distinct features from control cells, irrespective of the concentration tested or the duration of the stimulus.

### 3.3. Citicoline Stimulation Induces Global Changes in Intracellular Proteome After 6 h of Treatment

Upon validation of technical soundness, protein intensities were interrogated for differentially expressed proteins (DEPs). To this aim, proteomes of cells treated with citicoline (both doses) for 6 h were first analyzed and compared to the untreated cells, utilizing a moderated Bayesian *t*-test (Limma). Significance was defined by thresholds of log_2_FC ≥ ∣0.57∣ (corresponding to a ≥1.5-fold change in protein intensity) and FDR ≤ 0.05 for 1 mM citicoline and a more relaxed (for comparison purposes only) FDR ≤ 0.1 for 0.1 mM citicoline (FDR corresponds to the q.value calculated by Storey’s test within the limma analysis).

**Figure 3:**
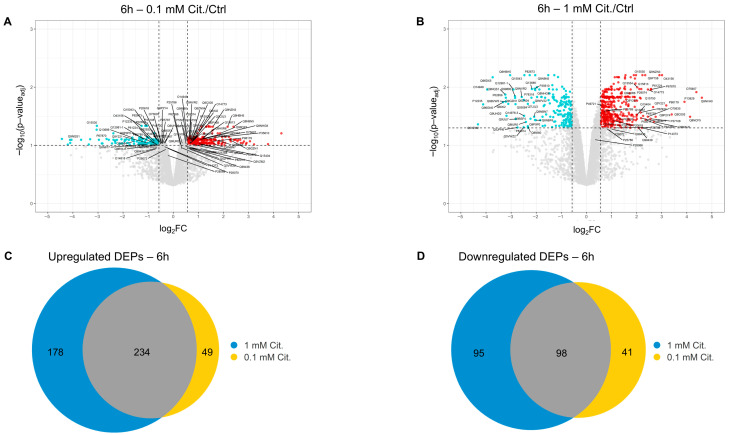
(**A**,**B**) Volcano plots illustrating the distribution of differentially expressed proteins in SHSY5Y cells treated with 0.1 mM citicoline (**A**) or 1 mM citicoline (**B**) for 6 h, compared to untreated control cells. Upregulated proteins are shown in red and downregulated proteins in turquoise. Proteins that did not pass the threshold set for significance are highlighted in grey. The dashed lines represent the significance thresholds for differential expression. For both panels, the log^2^FC threshold was set at log_2_FC|0.57|. The negative logarithm (base 10) of the adjusted *p*-value (−log_10_*p*-value_adj_) is plotted on the *y*-axis. In panel (**A**) (0.1 mM citicoline), proteins were filtered for *p*-value_adj_ ≤ 0.1, while in panel (**B**) (1 mM citicoline), the *p*-value_adj_ threshold was ≤ 0.05. Proteins with a log^2^FC ≥ 2 and subunits of the 20S proteasome (most starting with ‘P’ in their UniProt accession number) are labeled with their UniProt accession numbers. Labels in panel (**B**) point to the corresponding proteins in panel (**A**) to highlight the overlap in findings between the two citicoline concentrations. (**C**,**D**) Venn diagrams showing the overlap of differentially expressed proteins (DEPs) identified at 6 h of stimulation. Panel (**C**) shows the overlap of upregulated DEPs among cells treated with 1 mM citicoline, 0.1 mM citicoline, and untreated controls. Panel (**D**) shows the overlap of downregulated DEPs among cells treated with 1 mM citicoline, 0.1 mM citicoline, and untreated controls.

For the 1 mM citicoline and 6 h treatment condition, this analysis retrieved 412 upregulated proteins and 193 downregulated DEPs, and the sets of upregulated and downregulated DEPs closely overlapped with those computed for 0.1 mM citicoline (vs. Ctrl) (Figure 3A,D). The full list of proteins upregulated and downregulated by 1 mM citicoline (6 h) is provided in Supplementary Tables S1 and S2, respectively, along with evidence of those showing an analogous trend in the presence of 0.1 mM citicoline.

**Figure 4:**
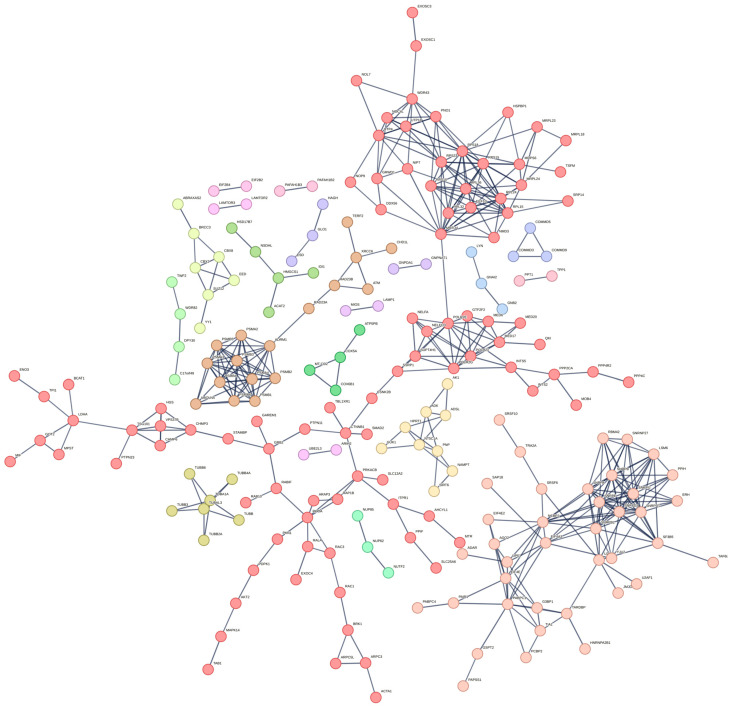
STRING Network analysis of proteins upregulated by 1 mM citicoline over 6 h of stimulation (compared to untreated cells). Only proteins with the highest degree (>0.9) of connection were retained in the plot. Clusters have been filtered using the k-mean method.

To identify enriched protein–protein interaction (PPI) networks and gain insights into the cellular mechanisms influenced by citicoline, proteins significantly upregulated or downregulated by 1 mM citicoline after 6 h of treatment (compared to untreated cells) were submitted separately to STRING Network software. Interactions were filtered for the highest confidence (score ≥ 0.9), and the resulting networks were partitioned using k-means clustering (n = 20) (Figure 4).

**Figure 5:**
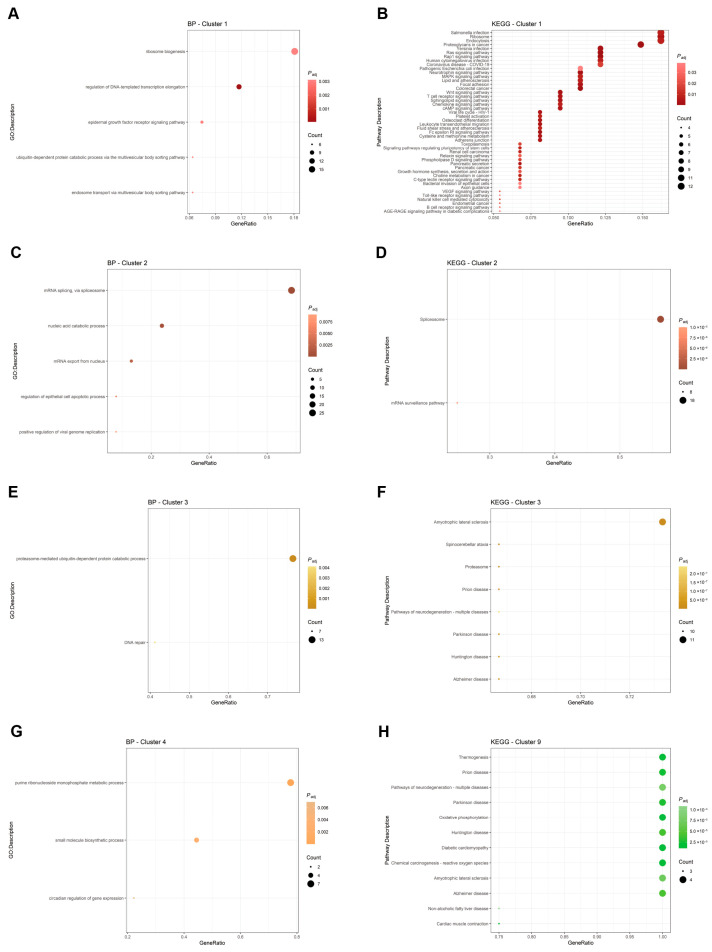
Biological Process (BP) and KEGG pathway charts derived from GO and KEGG analyses of clusters identified via PPI analysis in STRING. Specifically, panels (**A**,**B**) pertain to Cluster 1; panels (**C**,**D**) illustrate Cluster 2; and panels (**E**,**F**) show Cluster 3. Panel (**G**) displays the BP of Cluster 4, and panel (**H**) presents the KEGG analysis of Cluster 9. For clarity and consistency, the color associated with each cluster corresponds to the color assigned by the STRING software, as indicated in Supplementary Table S3. All data were filtered using a Benjamini–Hochberg adjusted *p*-value ≤ 0.01. The dots in the legend represent the gene count for each specific term identified.

In the case of upregulated proteins, a highly interconnected network was computed, comprising 405 nodes and 2233 edges, with an average node degree of 11 (PPI enrichment *p*-value: 0.0039). This dense connectivity suggests a coordinated cellular response to citicoline treatment. Further inspection revealed 20 unique and tightly packed protein clusters (Supplementary Table S3). Individual clusters were then subjected to Gene Ontology (GO) analysis to identify enriched biological processes, molecular functions, and cellular components, and to KEGG pathway analysis (Figure 5, Supplementary Figure S3, Supplementary Table S3). The following discussion focuses on a selected panel of clusters, prioritized for their high protein count, their translational relevance to neurodegeneration, and their effective identification of GO- and KEGG-enriched terms and pathways (clusters 1, 2, 3, 4, 9). However, a comprehensive list of all clusters and proteins is provided in Supplementary Table S3.

The first cluster (94 proteins) robustly highlighted an increase in the cell’s capacity for protein synthesis, primarily characterized by an enrichment of the proteins involved in ribosome biogenesis and structure. This cluster also showed significant enrichment in terms related to the regulation of DNA transcription, epidermal growth factor signaling pathway, G protein activity, and multiple terms associated with the multivesicular body (MVB) sorting pathway, including components of the Endosomal Sorting Complexes Required for Transport (ESCRT). KEGG analysis further confirmed the enrichment of key pathways critical for cell adhesion, motility, and neuronal mechano-transduction, notably Ras, Rap, MAPK, and Wnt signaling pathways. These findings collectively suggest that citicoline treatment robustly enhances fundamental cellular machinery for protein synthesis and quality control while significantly impacting cell signaling pathways critical for neuronal plasticity and stress response (Figure 5, Supplementary Figure S3, Supplementary Table S3). Notable proteins within this cluster included the following: Actin-related protein 2/3 complex subunit 3 [ARPC3] (implicating cytoskeletal dynamics), AKT Serine/Threonine Kinase 2 [AKT2] (involved in several signaling pathways), Phosphatidylinositol 3,4,5-trisphosphate-dependent GTPase [PIP3-GEF] (involved in PI3K signaling), Ras Homolog Family Member A [RhoA] (involved in signaling pathways related to G-proteins), Catenin beta-1 [CTNNB1] (a key component of Wnt signaling and cell adhesion), components of mRNA splicing machinery (Integrator complex subunit 2 [INTS2], U3 small nucleolar RNA-associated protein 18 homolog [UTP18]), mRNA transcription machinery (Mediator of RNA polymerase II transcription subunits -17 [MED17], -20 [MED20], and -6 [MED6], different DNA-directed RNA polymerases I, II, and III subunits), and mitochondrial ribosomal proteins (M26 [MRPL26], M27 [MRPL27], S6 [MRPS6]), indicating an enhanced capacity for mitochondrial protein synthesis (Figure 5, Supplementary Figure S3, Supplementary Table S3).

The second cluster (39 proteins) was densely populated by proteins crucial for mRNA capping, binding, and particularly splicing. This included various Polyadenylate-binding proteins (e.g., PABPC1 and PABPC4) and a comprehensive suite of components from the major splicing complexes, including U1 (snRPA1), U2, U4/U6, and U5 small nuclear ribonucleoproteins (snRNPs), along with associated splicing factors (e.g., U2AF2/U2AF1). Correspondingly, GO analysis prominently highlighted terms for mRNA binding, splicing, and nuclear export, with strong enrichment in the spliceosome and mRNA surveillance pathway (KEGG analysis). The significant upregulation of this entire machinery points towards a heightened capacity for stringent post-transcriptional control over gene expression, representing a potentially protective adaptation given the recognized role of RNA processing dysregulation in neurodegenerative diseases (Figure 5, Supplementary Figure S3, Supplementary Table S3).

The third cluster (17 proteins) was remarkably characterized by the presence of several core members of the ubiquitin-proteasome system (UPS). Specifically, we identified 9 out of the 14 α- and β-proteins that constitute the 20S catalytic particle of the proteasome. Consistent with this, GO analysis highlighted enrichment for the proteasome complex, the ubiquitin proteasome system, and DNA repair.

Accordingly, GO analysis for this cluster highlighted enrichment for terms such as proteasome complex, ubiquitin proteasome system, and DNA repair (Figure 5, Supplementary Figure S3, Supplementary Table S3). The inclusion of DNA repair terms is strongly linked to the presence of UV excision repair protein RAD23 homolog A [RAD23A] and B [RAD23B], as well as Serine-protein kinase ATM [ATM], key players in DNA damage response. Critically, KEGG analysis for this cluster retrieved a long list of neurodegenerative diseases, including Amyotrophic Lateral Sclerosis, Ataxia, Huntington’s, Parkinson’s, and Alzheimer’s diseases (Figure 5, Supplementary Figure S3, Supplementary Table S3). This compelling upregulation of the UPS and associated stress response pathways strongly suggests that citicoline enhances the cell’s capacity for clearing misfolded or damaged proteins and responding to cellular stress, mechanisms that are fundamentally impaired in many neurodegenerative conditions, including glaucoma.

The fourth cluster, a smaller group of nine proteins was populated by proteins involved in energetic metabolism and nucleotide synthesis and salvage pathways. Specific members included Hypoxanthine-guanine phosphoribosyltransferase [HPRT1], Adenosine kinase [ADK], Nicotinamide phosphoribosyltransferase [NAMPT] (an enzyme in NAD+ biosynthesis), NAD-dependent protein deacetylase sirtuin-6 [SIRT6], and Purine nucleoside phosphorylase [PNP] (Figure 5, Supplementary Figure S3, Supplementary Table S3). Consistently with their known biological roles, GO charts for this cluster highlighted terms for purine ribonucleoside monophosphate metabolic processes, small-molecule biosynthetic processes, and pentosyltransferase activity. No enriched cellular component (CC) or KEGG terms were found for this cluster, suggesting a focused impact on specific metabolic processes. The upregulation of these enzymes implies that citicoline treatment may be bolstering cellular energy reserves and the capacity for nucleotide synthesis, crucial for the high metabolic demands of neurons and for repairing cellular damage (Figure 5, Supplementary Figure S3, Supplementary Table S3).

Regarding the less densely populated clusters (less than 7 proteins per cluster), besides the upregulation of several tubulin α and β chains (cluster 5, indicating cytoskeletal reorganization), and a panel of proteins involved in transcriptional repression (such as Chromobox protein homologs [CBX], Polycomb protein EED [EED], Transcriptional repressor protein YY1 [YY1]), and additional components of chromatin complexes (e.g., Chromatin complex subunit BAP18 [DCAF10] in clusters 6 and 8), clusters 7 and 9 are particularly noteworthy (Figure 5, Supplementary Figure S3, Supplementary Table S3).

While cluster 7 reported several genes involved in cholesterol biosynthetic pathways (Acetyl-CoA acetyltransferase [ACAT1/ACAT2], Hydroxymethylglutaryl-CoA synthase [HMGCS1], 3-keto-steroid reductase [HSD17B7], Isopentenyl-diphosphate Delta-isomerase 1 [IDI1], Sterol-4-alpha-carboxylate 3-dehydrogenase, decarboxylating [NSDHL]), suggesting potential modulation of lipid metabolism, cluster 9 casts light on novel findings for citicoline’s mechanism of action directly related to mitochondrial function. This cluster notably included ATP synthase F(0) complex subunit B1 [ATP5PB], Cytochrome c oxidase subunit 5A [COX5A], 6B1 [COX6B1], and 2 [COX6B2], all of which are integral components of the mitochondrial respiratory chain and oxidative phosphorylation. Accordingly, GO and KEGG analysis of these proteins retrieved terms such as proton transmembrane transporter activity, electron transfer activity, and the same panel of human neurodegenerative diseases already observed in the KEGG analysis of cluster 3 (e.g., Alzheimer’s, Parkinson’s) (Figure 5, Supplementary Figure S3, Supplementary Table S3). Further enriched terms included thermogenesis and oxidative phosphorylation. The upregulation of these critical mitochondrial components strongly suggests that citicoline enhances mitochondrial bioenergetic capacity, a vital neuroprotective mechanism given that mitochondrial dysfunction is a central pathological feature in glaucoma and other neurodegenerative conditions.

**Figure 6:**
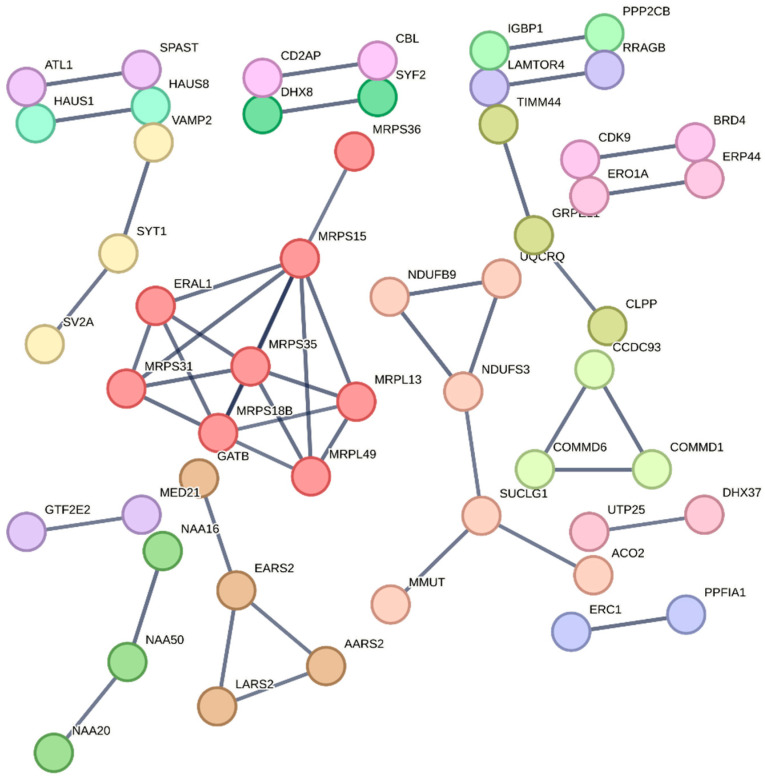
STRING Network analysis of proteins downregulated by 1 mM citicoline over 6 h of stimulation (compared to untreated cells). Only proteins with the highest degree (>0.9) of connection were retained in the plot. Clusters have been filtered using the k-mean method.

In the case of downregulated proteins, despite a relatively high number of individual proteins, the same STRING Network analysis settings retrieved only three but highly informative individual clusters (Figure 6 and Supplementary Table S4).

The first downregulated cluster was populated by an array of mitochondrial ribosomal proteins, including L13 [MRPL13], L49 [MRPL49], S15 [MRPS15], S18b [MRPS18B], S35 [MRPS35], S36 [MRPS36], and S37 [MRPS37]. Correspondingly, the relative enriched GO chart included terms for mitochondrial translation and mitochondrial gene expression and mitochondrial ribosome (Supplementary Figure S4). The downregulation of mitochondrial ribosomal proteins is a significant finding. While seemingly contradictory to the upregulated mitochondrial components in cluster 9 of the upregulated proteins, this might suggest a dynamic and complex mitochondrial adaptation to citicoline treatment. It could indicate a shift in mitochondrial protein synthesis or a feedback mechanism, potentially prioritizing nuclear-encoded mitochondrial components over those typically synthesized within the mitochondrion, or a transient downregulation to allow for structural reorganization. This warrants further investigation to understand the precise implications for mitochondrial health and function in the long term.

Conversely, the second downregulated cluster, although it did not retrieve specific enriched terms, was characterized by the presence of several key enzymes of the tricarboxylic acid cycle (TCA) (Supplementary Figure S4, Supplementary Table S4). These included Aconitate hydratase [ACO2], Methylmalonyl-CoA mutase [MMUT], NADH dehydrogenase [ubiquinone] 1 beta subcomplex subunit 9 [NDUFB9], NADH dehydrogenase [ubiquinone] iron-sulfur protein 3 [NDUFS3], Succinate-CoA ligase [ADP/GDP-forming] subunit alpha [SUCLG1], and Cytochrome b-c1 complex subunit 8 [UQCRB]. The downregulation of these central components of the TCA cycle, which is the primary hub for cellular energy production, suggests a potential modulation of metabolic flux under citicoline treatment. This could imply a temporary reduction in glucose oxidation, perhaps favoring alternative energy substrates or indicating a metabolic reprogramming in response to the drug’s effects. Further metabolic profiling would be beneficial to fully understand the consequences of this observed downregulation.

### 3.4. Citicoline Stimulation for 18 h Is Not Accompanied by Proteome Perturbations

To determine whether the substantial remodeling of the intracellular proteome observed after 6 h of citicoline stimulation represented a transient phenomenon or a prolonged drug effect, a time-course analysis was conducted using Limma. This involved comparing the log_2_ fold change (log_2_FC) in protein intensity across different time points and treatment groups, utilizing a time*group interaction model to specifically identify proteins whose response to citicoline varied significantly over time. Significance was defined by thresholds of log_2_FC ≥ ∣0.57∣ and FDR ≤ 0.05.

At the 18 h time point, the proteomic alterations were markedly different from those observed at 6 h. Interestingly, through this time-dependent interaction effect, a distinct set of proteins still exhibited significant changes at 18 h, indicating a sustained or complex temporal regulation. These included the following: Zinc Finger Protein 219 (ZNF219) [MAZ] (log_2_FC: −8), 3-phosphoinositide-dependent protein kinase 1 [PDK1] (log_2_FC: −1.8), Putative ATP-dependent RNA helicase DHX57 [DHX57] (log_2_FC: −1.8), Retinol dehydrogenase 14 [RDH14] (log_2_FC: 2), and Ankyrin repeat and SOCS box protein 9 [ASB9] (log_2_FC: 6). The fact that these specific proteins still show significant changes in the time*group interaction context suggests they are either subject to a more prolonged citicoline effect or their regulation is part of a complex, adaptive temporal response.

**Figure 7:**
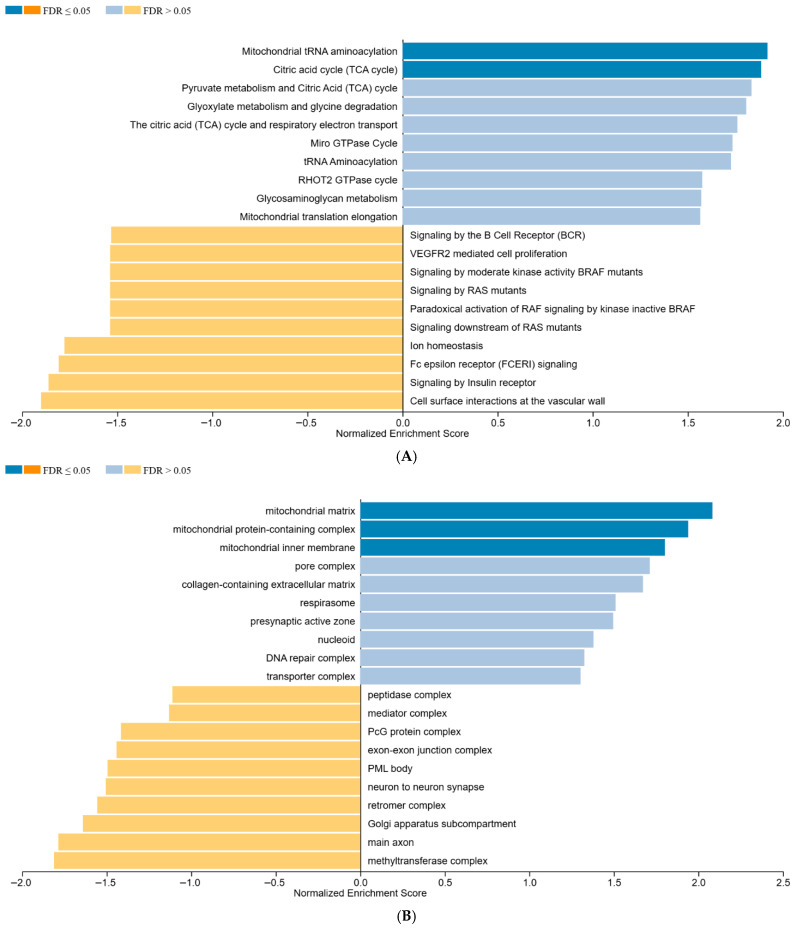
GSEA of proteins ranked by log2FC for the comparison between 1 mM citicoline over 18 h of stimulation and untreated cells. Panel (**A**) shows the outcome of Reactome analysis; Panel (**B**) shows the outcome of the GO Cellular Component. All enriched terms are here shown. Those identified upregulated with FDR ≤ 0.05 are shaded darker, as indicated in the legend. Downregulated terms did not reach FDR≤ 0.05.

To further characterize the sustained biological effects, if any, beyond direct differential expression, the protein lists from the 18 h time*group analysis were submitted to Gene Set Enrichment Analysis (GSEA). Among the analyses performed, we found enriched terms when uploading the protein list to Reactome pathways and Cellular Component (CC) charts within Gene Ontology (GO). Both these approaches consistently highlighted terms related to mitochondria and the TCA cycle, suggesting that while individual protein expression changes were largely resolved, the underlying cellular metabolic machinery related to energy production might still be subtly modulated or recovering (Figure 7A,B).

To explicitly confirm the transient nature of citicoline’s overall proteomic effects, a canonical two-group comparison of proteomes at 18 h (comparing both citicoline concentrations to untreated cells) was performed using Limma. This approach confirmed that the widespread proteomic perturbations identified at 6 h were no longer apparent. Specifically, no proteins were found to be significantly upregulated or downregulated when applying the same conservative threshold (i.e., log_2_FC ≥ ∣0.57∣, FDR ≤ 0.05) used for the 6 h analysis, except for the enhancer of polycomb homolog 1 among the downregulated proteins. Supplementary Tables S5 and S6 report the list of proteins upregulated and downregulated in the presence of 1 mM citicoline vs. untreated cells after 18 h of stimulation and filtering for p.mod ≤ 0.01 (Supplementary Figure S5).

To provide further evidence for this transient effect, we specifically tracked the proteins that were originally significantly upregulated and downregulated after 6 h of 1 mM citicoline stimulation. After 18 h of treatment, only a very small fraction of these proteins retained their initial directional change at a relaxed significance level: a mere 10 out of 412 originally upregulated proteins still showed a positive log_2_FC (compared to untreated cells) with a moderate *p*-value (p.mod ≤ 0.01) and, similarly, only 10 out of 192 originally downregulated proteins retained a negative log_2_FC (compared to untreated cells) again with a moderate *p*-value (p.mod ≤ 0.01).

The identity of upregulated proteins is the following: Cytoplasmic polyadenylation element-binding protein 4 [CPEB4], Protein VAC14 homolog [VAC14], ER membrane protein complex subunit 10 [EMC10], Nucleus accumbens-associated protein 1 [NACAP1], E3 ubiquitin-protein ligase KCMF1 [KCMF1], Brefeldin A-inhibited guanine nucleotide-exchange protein 2 [BIG2], Queuosine 5′-phosphate N-glycosylase/hydrolase [QNGH], Single-stranded DNA-binding protein 3 [SSBP3], Putative peptidyl-tRNA hydrolase PTRHD1 [PTRHD1], and Zinc finger protein RFP [RFP].

The identity of downregulated proteins is the following: Metal cation symporter ZIP14 [ZIP14], Nucleic acid dioxygenase ALKBH1 [ALKBH1], Galanin peptides [GAL], Sodium/hydrogen exchanger 8 [NHE8], Probable ATP-dependent RNA helicase DHX37 [DHX37], Transcription factor GATA-4 [GATA4], COMM domain-containing protein 6 [COMMD6], Enhancer of polycomb homolog 1 [EPC1], Heparan sulfate 2-O-sulfotransferase 1 [HS2ST1], and Small ribosomal subunit protein uS15m [uS15m].

Submission of these proteins (separately for upregulated and downregulated) to STRING analysis did not retrieve any network or evidence of association between them, envisaging that the behavior observed over time was unrelated to any specific biological or metabolic process.

This confirms that the major proteomic shifts induced by citicoline at 6 h were largely transient, with the proteome largely returning to a state comparable to untreated cells by 18 h.

### 3.5. Validation of Proteomics Data by Western Blotting Analysis of a Selection of Proteins Stimulated by Citicoline After 6 h of Treatment

**Figure 8:**
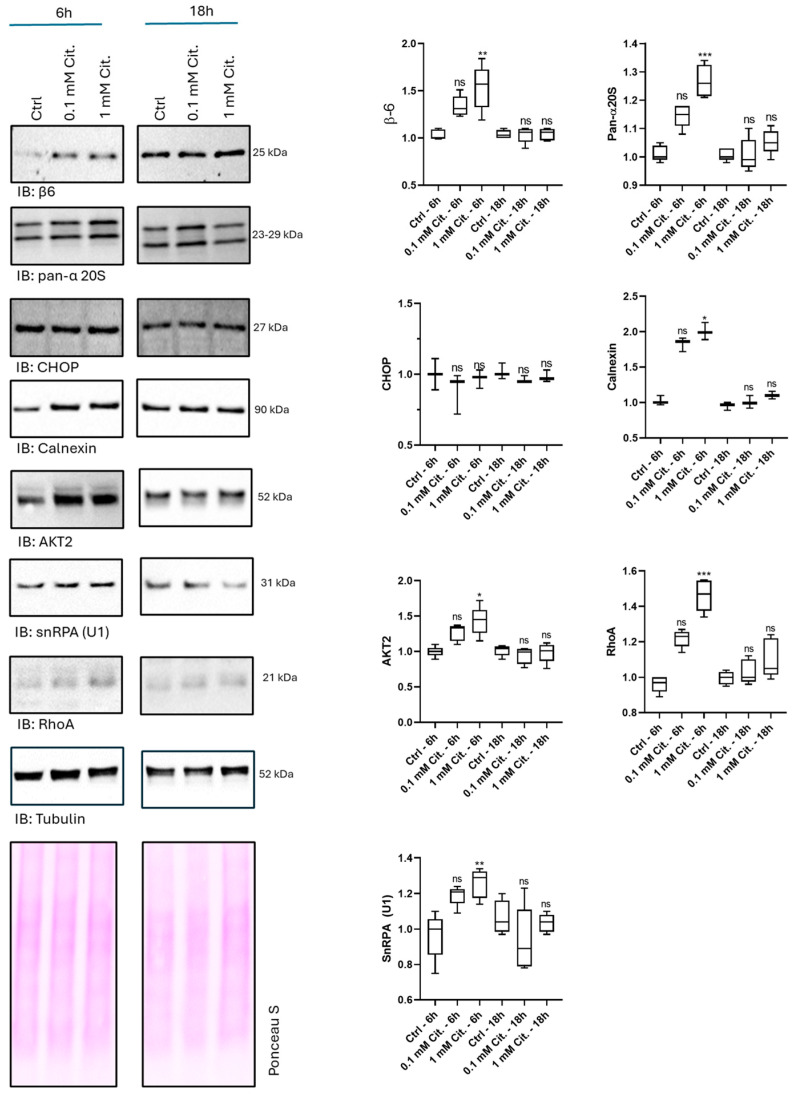
Western blotting panel showing the fold change in protein (i.e., β6, pan-α 20S, calnexin, CHOP, AKT2, RhoA, SNRPA-U1 proteins) intensity in treated (1 mM and 0.1 mM citicoline, 6 h, 18 h) vs. untreated (ctrl) cells. Tubulin staining is shown together with total protein content stained by Ponceau S, which was used for protein intensity normalization across groups. Box and whisker plots (right panel) report the median (internal line) and mix–max values per experimental condition. A nominal value 1 was attributed to the intensity of the first lane of ctrl cells. * *p* ≤ 0.05, ** *p* ≤ 0.01, *** *p* ≤ 0.001, Kruskal–Wallis non-parametric test followed by Dunn’s correction for multiple comparisons. ns = not significant.

To further investigate the transient induction of proteins observed after 6 h of citicoline treatment, we conducted a confirmatory Western blotting study (Figure 8). We utilized the same cell lysates (prepared with urea buffer) as in the shotgun proteomics study, supplementing them with a confirmatory analysis on three independent biological replicates. In addition to representative subunits of the 20S catalytic core—selected to reconcile the proteomics data with those previously reported by our group in cell extracts not including the nuclear fraction [24]—we expanded the investigation to include key proteins relevant to the observed proteomic changes. We probed filters against the endoplasmic reticulum (ER) marker calnexin and the ER stress marker CHOP [38]. Calnexin was specifically chosen because it showed upregulation at 6 h of citicoline stimulation in our proteomics data (log2FC: 0.57; q.mod: 0.03), although it was not part of any identified protein clusters. We also included the key proteins involved in signaling pathways and cell metabolism that were grouped in Clusters 1 and 2. Immunoblotting revealed a significant upregulation of the β6 subunit (encoded by the PSMB1 gene) and all α-subunits (except PSMA4, using a validated pan-α20S antibody) in cells stimulated for 6 h with 1 mM citicoline compared to untreated control cells. Under the same experimental conditions (i.e., 6 h), calnexin levels mirrored the increased pattern in the presence of citicoline doses compared to untreated cells. CHOP, however, remained unchanged regardless of the citicoline dose or exposure time, suggesting the observed response was not linked to overt ER stress. A similar trend of induction by 1 mM citicoline was confirmed for AKT2, RhoA, and snRPA (U1) proteins, further strengthening the proteomics data. Across the entire study, the 0.1 mM citicoline dose, although seemingly found to stimulate the levels of all these proteins (except for CHOP), did not reach the statistical threshold of significance after multiple-comparison testing using Dunn’s method. This result was expected, given the relatively low sample size of the study; however, the trend followed by protein intensities was suggestive of a dose-dependent effect. Notably, all proteins investigated returned to basal levels of unstimulated cells after 18 h of citicoline treatment. At this time point, all proteins investigated showed levels comparable to that of untreated cells.

## 4. Discussion

This unbiased shotgun proteomics study provides robust evidence that citicoline, beyond its established pharmacological and metabolic activities in phospholipid and neurotransmitter biosynthesis, triggers a rapid and profound, yet largely transient, remodeling of the intracellular proteome when administered to SH-SY5Y cells.

Over the last two decades, extensive preclinical (e.g., SH-SY5Y cells, Trabecular Meshwork Cells, and rodent models of glaucoma) and clinical data have highlighted the undeniable cytoprotective activities of citicoline against various pathological conditions and toxic stimuli, including intracerebral hemorrhage, ischemia, glutamate excitotoxicity, and redox imbalance [13,15,16,17,20,21,22,23,24].

Citicoline is currently an adjunctive treatment for glaucoma, used in conjunction with hypotensive medications [7,8,9,10,13,14,15,16,17,18], but an international phase III clinical trial for citicoline eyedrops is currently underway (NCT05710198) to assess, as first-line medication, the therapeutic effect of the drug on visual field deterioration of glaucoma subjects during a 3-years follow-up period.

Citicoline’s action is multifaceted. It acts as a precursor for crucial cell membrane components (phosphatidylcholine and sphingomyelin) and neurotransmitters (dopamine and acetylcholine) [3,17]. This is thought to enhance neuron homeostasis, synaptic transmission, and plasticity, ultimately improving cognitive and visual function. However, the exact systems-level mechanisms of its cytoprotective effects require further experimental validation.

Our results, particularly the differentially expressed proteins (DEPs) identified in cells stimulated with both 1 mM and 0.1 mM citicoline after 6 h of treatment, introduce a compelling working hypothesis: the neuroprotective activity of the drug likely relies upon rapidly priming several central pathways crucial for cell metabolism and homeostasis. These include fundamental processes such as gene transcription, mRNA splicing, protein translation, intracellular proteostasis, and critically, mitochondrial metabolism.

The striking overlap in DEP sets between the two citicoline doses (0.1 mM and 1 mM) after 6 h further suggests that a significant proteomic response is initiated even at lower concentrations, hinting at a robust and potentially saturable induction of these protective mechanisms.

In this regard, cell homeostasis is primarily dependent on a balanced proteostasis network (PN). PN identifies the complex equilibrium between protein synthesis, folding, trafficking, and degradation [39,40,41,42].

Alterations in any of these steps can lead to protein overload and proteotoxicity, a common feature in various cellular stressors and neurodegenerative conditions. Eukaryotic cells have evolved an intricate network of chaperones, proteolytic systems (such as the Ubiquitin–Proteasome System [UPS] and autophagy), and scavenging systems to adequately preserve the PN. The enrichment of this molecular machinery following pharmacological stimulation is often considered a prerequisite for improving cellular tolerance to stressors, a concept consistent with hormesis [29].

In this proteomic study, a heterogeneous repertoire of cytoprotective mechanisms was indeed significantly upregulated by citicoline stimulation at 6 h. This included components of protein synthesis (ribosome biogenesis, protein translation), mRNA processing (splicing machinery, RNA surveillance), and particularly the ubiquitin–proteasome system (UPS). This observation was further supported by the lack of induction of typical ER stress markers like CHOP or calnexin regardless of the citicoline dose used [38]. This absence of stress induction suggests that citicoline is not acting as a mild stressor itself to trigger these responses, but rather as a genuine modulator enhancing intrinsic cellular resilience pathways. This aligns with the long-established safety profile of citicoline, even at high doses.

Among the cytoprotective mechanisms stimulated by citicoline, the UPS, and the proteasome in particular, warrant further detailed discussion.

The UPS is the major surveyor of intracellular protein homeostasis [25,43,44]. The proteolytic activity of the 26S holoenzyme on ubiquitylated substrates and the still-debated role of the uncapped 20S in degrading oxidized and unfolded proteins are key for PN balance [45,46,47].

In our previous study, citicoline was uncovered as an allosteric modulator of 20S catalytic activity [24]. While that study provided strong in vitro and cellular evidence for an allosteric structural effect on proteasome composition, this current proteomic study significantly broadens our understanding of citicoline’s influence on UPS configuration. Herein, by analyzing the whole cell lysate (including the nucleus, which is enriched in assembled proteasome particles often excluded from previous analyses due to methodological challenges), we found that citicoline induced the upregulation and intracellular accumulation of 20S proteasome subunits. This suggests that citicoline not only modulates proteasome activity, but also increases the overall cellular capacity for protein degradation, potentially by enhancing the population of proteasome assemblies inside compartments like the nucleus. This evidence strongly suggests that many of the protective effects of the drug are dependent on the multi-faceted modulation of this central pathway of cell homeostasis.

In addition to mechanisms of PN regulation, it is critical to emphasize that citicoline was found to strongly stimulate transcriptional, translational, and mRNA splicing processes, and to promote nucleotide biosynthetic pathways, thereby envisaging a robust anabolic activity. This consideration is further supported by the large number of histone and transcriptional regulators identified among both upregulated and downregulated DEPs. The impressive increase in splicing factors is of particular interest, as this process has been associated with the mechanisms contrasting senescence and aging-related processes, with pharmacological stimulation of splicing factors viewed as a potential senolytic treatment [48,49]. While precise interpretation of citicoline’s global effect on gene expression mechanisms at the level of specific nuclear factors remains challenging and beyond the scope of this study (especially considering potential biases in protein identification reflective of protein accessibility to trypsin digestion), the overall enhancement of transcriptional and translational machinery points to a fundamental cellular revitalization.

A significant novel perspective introduced by the depth of proteome coverage achieved in this study is that citicoline strongly stimulates mitochondrial processes and metabolism. Specifically, our analysis revealed the upregulation of key components of the mitochondrial respiratory chain and oxidative phosphorylation (Cluster 9 of upregulated proteins). This direct enhancement of mitochondrial bioenergetic capacity is vital for the homeostasis of nervous cells and represents a crucial neuroprotective mechanism, given that mitochondrial alteration is considered a main pathogenic mechanism in glaucoma onset and progression [50,51].

Conversely, we also observed a downregulation of mitochondrial ribosomal proteins and enzymes of the tricarboxylic acid (TCA) cycle among the downregulated clusters. This seemingly contradictory finding suggests a complex and dynamic mitochondrial adaptation under citicoline treatment. It might indicate a shift in mitochondrial protein synthesis, perhaps prioritizing nuclear-encoded components over those translated locally within the organelle, or a metabolic reprogramming aimed at optimizing energy efficiency or reducing oxidative stress. This multifaceted modulation, including potential shifts in metabolic flux, underlines citicoline’s sophisticated influence on mitochondrial health. While the precise molecular basis of this complex mitochondrial modulation remains to be fully elucidated and will be matter of future studies, this finding strongly stimulates the design of additional studies to confirm and detail citicoline’s mechanism of action on this organelle, which is central for neuronal vitality. Uncovering these features represents a compelling additional pharmacological rationale for citicoline treatment not only in glaucoma, but also across a broader range of neurodegenerative diseases [50,52,53,54].

Finally, our time-course analysis at 18 h provided crucial insights into the temporal dynamics of citicoline’s effects. The widespread proteomic perturbations observed at 6 h were largely transient, with the majority of proteins returning to levels comparable to untreated cells by 18 h. This suggests an adaptive “hit-and-run” mechanism where citicoline elicits a robust, acute cellular response that primes the cell for improved stress resistance and homeostasis, rather than causing sustained, pervasive alterations. However, our Limma time-group interaction analysis did reveal that a select subset of regulatory proteins maintained significant differential regulation at 18 h, although they did not reach the FDR threshold set. The persistence of these specific changes, particularly in a key transcriptional regulator like ZNF219/MAZ (involved in chromatin organization and gene expression), suggests that while the broad adaptive response is transient, citicoline might induce more prolonged or subtle modifications in specific regulatory nodes that could have lasting impacts on cellular memory or resilience pathways. Furthermore, the very small overlap of overall DEPs at 18 h (only ten upregulated and ten downregulated from the 6 h lists at a relaxed *p*-value) underscores this transient nature. While a more specific dose-dependent effect at 18 h was suggested in earlier drafts, the overarching finding is the return to baseline for most proteins, with only a few specific, critical components showing sustained changes in the interaction model. This transient but impactful proteome remodeling could underpin the long-term clinical benefits observed with citicoline.

This study has several limitations that warrant acknowledgment. First, as an in vitro cell model, our data may not fully reflect the complexities of in vivo biological systems or translate directly to clinical outcomes. Second, the limited sample size of this study constrains the power to draw definitive statistical conclusions. For this reason, we employed the Limma approach, which enhances statistical robustness by borrowing variance information across proteins. Third, and importantly, while our findings introduce a novel working hypothesis regarding citicoline’s pharmacology, further research is needed to elucidate the precise molecular mechanisms underlying the observed in vitro effects. While the citicoline doses and stimulation timing align with the previous literature (including studies using SH-SY5Y cells), the findings may suffer from poor generalizability and be limited to the specific cell strain and working conditions employed. Therefore, future pharmacological and proteomic studies characterized by citicoline-induced proteome perturbations in animal nervous tissue are necessary to provide a more robust proof of concept.

In conclusion, the results obtained provide a more comprehensive and nuanced interpretation of citicoline’s effects on the cell proteome, casting light on the specific pathways and processes (including profound impacts on the UPS, RNA metabolism, and particularly, both upregulated and downregulated aspects of mitochondrial metabolism) previously unassociated with the full pharmacology of this drug. This study significantly fills a knowledge gap regarding its multifaceted mechanisms of action and further stimulates its therapeutic application across a broader range of human diseases beyond its current indications, especially those characterized by proteostasis imbalance and mitochondrial dysfunction, such as glaucoma and other neurodegenerative disorders. Future studies should aim to functionally validate these specific proteomic changes in vivo and explore the long-term physiological consequences of this transient, yet strategically impactful, cellular reprogramming.

## Data Availability

The mass spectrometry proteomics data have been deposited to the ProteomeXchange Consortium via the PRIDE partner repository [31,54]. Project accession: PXD061053; Token: ffp7JqH77Kog.

## Supplementary Materials

The following supporting information can be downloaded at: https://www.mdpi.com/article/10.3390/pharmaceutics18010061/s1, Figure S1: Box and whiskers plot reporting the fold-change of cell count, determined by MTS assay, of SH-SY5Y cells stimulated with 0.1 mM and 1 mM for 24 h; Figure S2: (A) Box and whiskers showing the median and mix max values (including outliers) of log2 transformed protein intensities of each biological replicate before normalization, (B) Density plot showing the log2 transformed intensities before normalization of proteins identified in each biological replicate, (C) Correlogram reporting the Person coefficient of correlation computed for each experimental group vs the others; Figure S3: The Cellular Component (CC) and Molecular Function (MF) charts, derived from GO analysis of clusters identified via PPI analysis (STRING) from the list of proteins upregulated by 1 mM citicoline (vs. untreated cells, 6 h), are presented; Figure S4: The Biological Function (BP), Molecular Function (MF) and Cellular Component (CC) charts, derived from GO analysis of clusters identified as downregulated (1 mM citicoline vs. untreated cells, 6 h) via PPI analysis (STRING), are presented; Figure S5: Volcano plot showing the distribution of upregulated (red) and downregulated (turquoise) proteins in treated (1 mM citicoline) vs untreated (Ctrl) cells after 18 h of stimulation; Figure S6: Uncropped Western blotting filters used for Wb panel of Figure 8; Table S1: Reported in this table are the proteins upregulated by 1 mM citicoline (compared to untreated cells) after 6 h; Table S2: Reported in this table are the proteins downregulated by 1 mM citicoline (compared to untreated cells) after 6 h; Table S3: The table presents clusters of upregulated proteins (1 mM citicoline vs. untreated cells, 6 h), which were identified through PPI analysis in STRING (refer to Supplementary Table S1); Table S4: The table presents clusters of downregulated proteins (1 mM citicoline vs. untreated cells, 6 h), which were identified through PPI analysis in STRING (refer to Supplementary Table S1); Table S5: Reported in this table are the proteins upregulated by 1 mM citicoline (compared to untreated cells) after 18 h of stimulated, with data filtered for p.mod ≤ 0.01; Table S6: Reported in this table are the proteins downregulated by 1 mM citicoline (compared to untreated cells) after 18 h of stimulation, with data filtered for p.mod ≤ 0.01.
