# Supplementary material for: Citicoline Triggers Proteome Remodeling and Proteostatic Adaptation: Evidence from Shotgun Proteomics
Source: Pharmaceutics. 2026 Jan 1;18(1):61. doi: 10.3390/pharmaceutics18010061 (PMC12845156; doi:10.3390/pharmaceutics18010061)
Supplement: Supplementary file 1 [file pharmaceutics-18-00061-s001.zip › pharmaceutics-3910289-supplementary.pdf]

SUPPLEMENTARY FIGURES

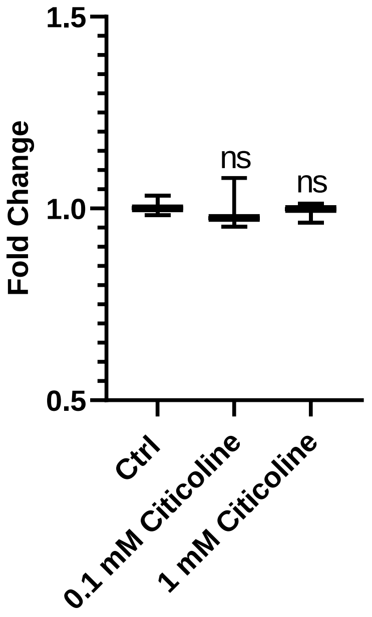

Suppl. Figure S1: Box and whiskers plot reporting the fold-change of cell count, determined by MTS assay, of SH-SY5Y cells stimulated with 0.1 mM and 1 mM for 24h. Data were derived by interpolation of optical density (n=3). Box and whiskers plot showing the fold change calculated for cell count between groups. A nominal value of 1 was attributed to a reference Ctrl sample. Non-parametric Kruskal-Wallis test. Ns=not significant.

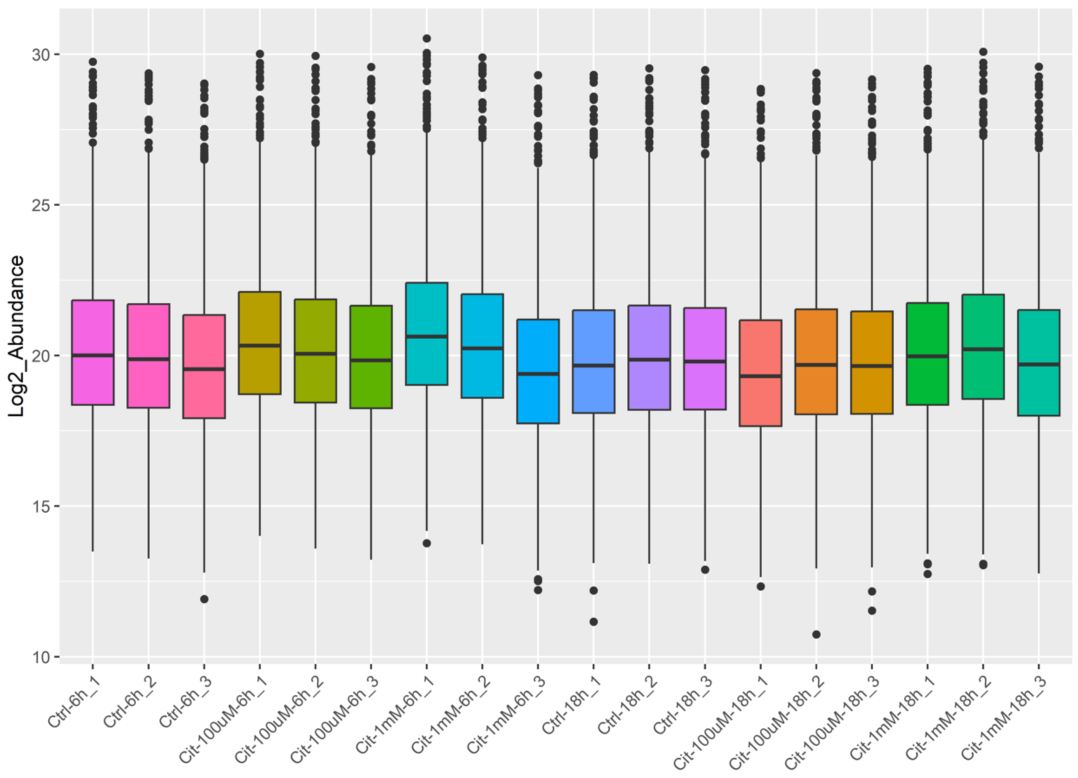

Suppl. Figure S2A: Box and whiskers showing the median and mix max values (including outliers) of log2 transformed protein intensities of each biological replicate before normalization.

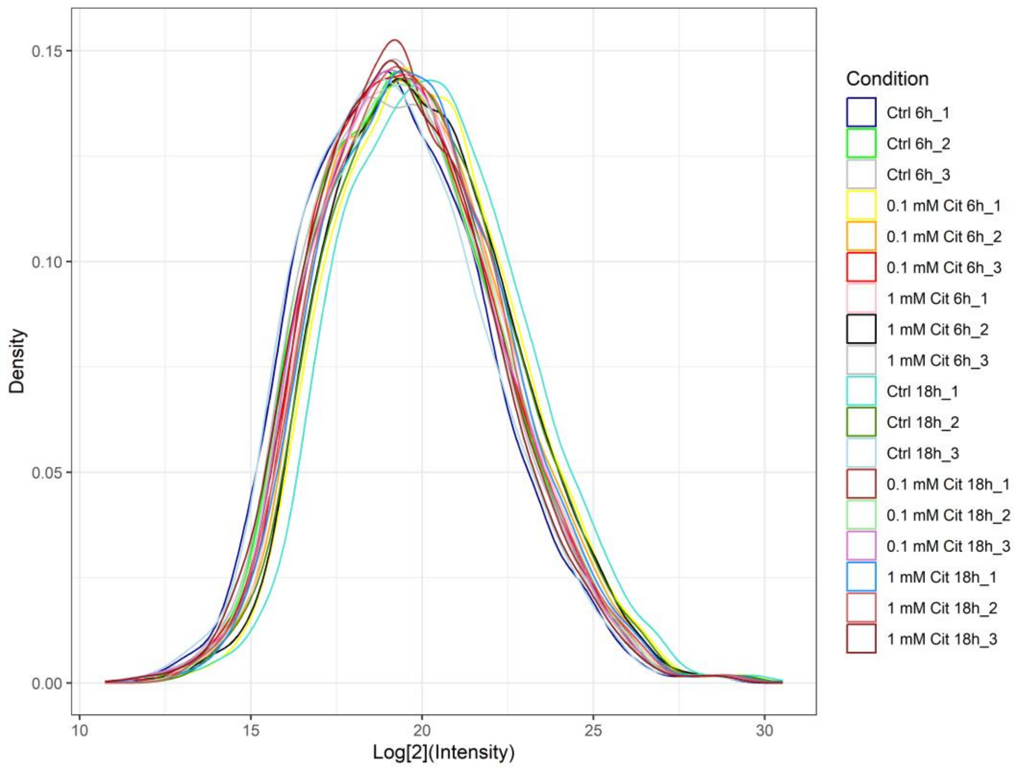

Suppl. Figure S2B: Density plot showing the log2 transformed intensities before normalization of proteins identified in each biological replicate.

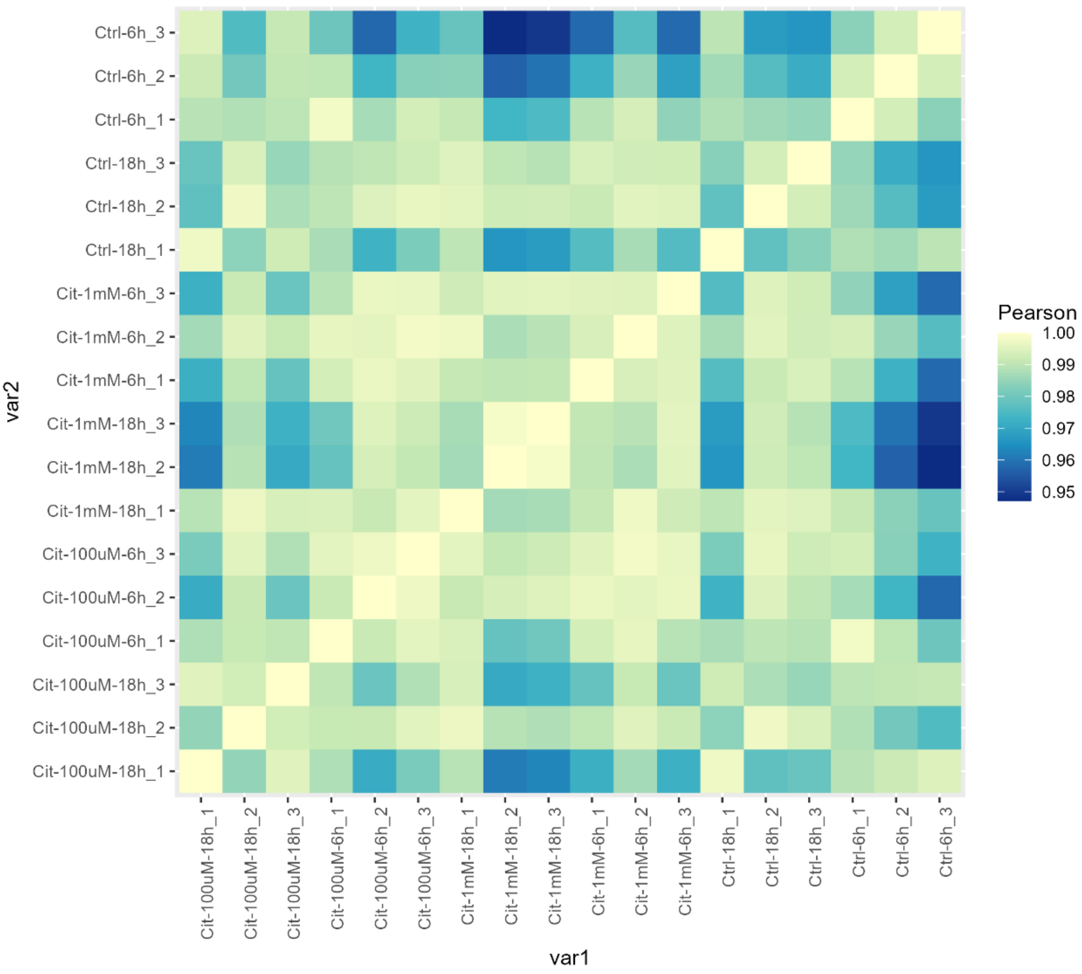

Suppl. Figure S2C: Correlogram reporting the Person coefficient of correlation computed for each experimental group vs the others.

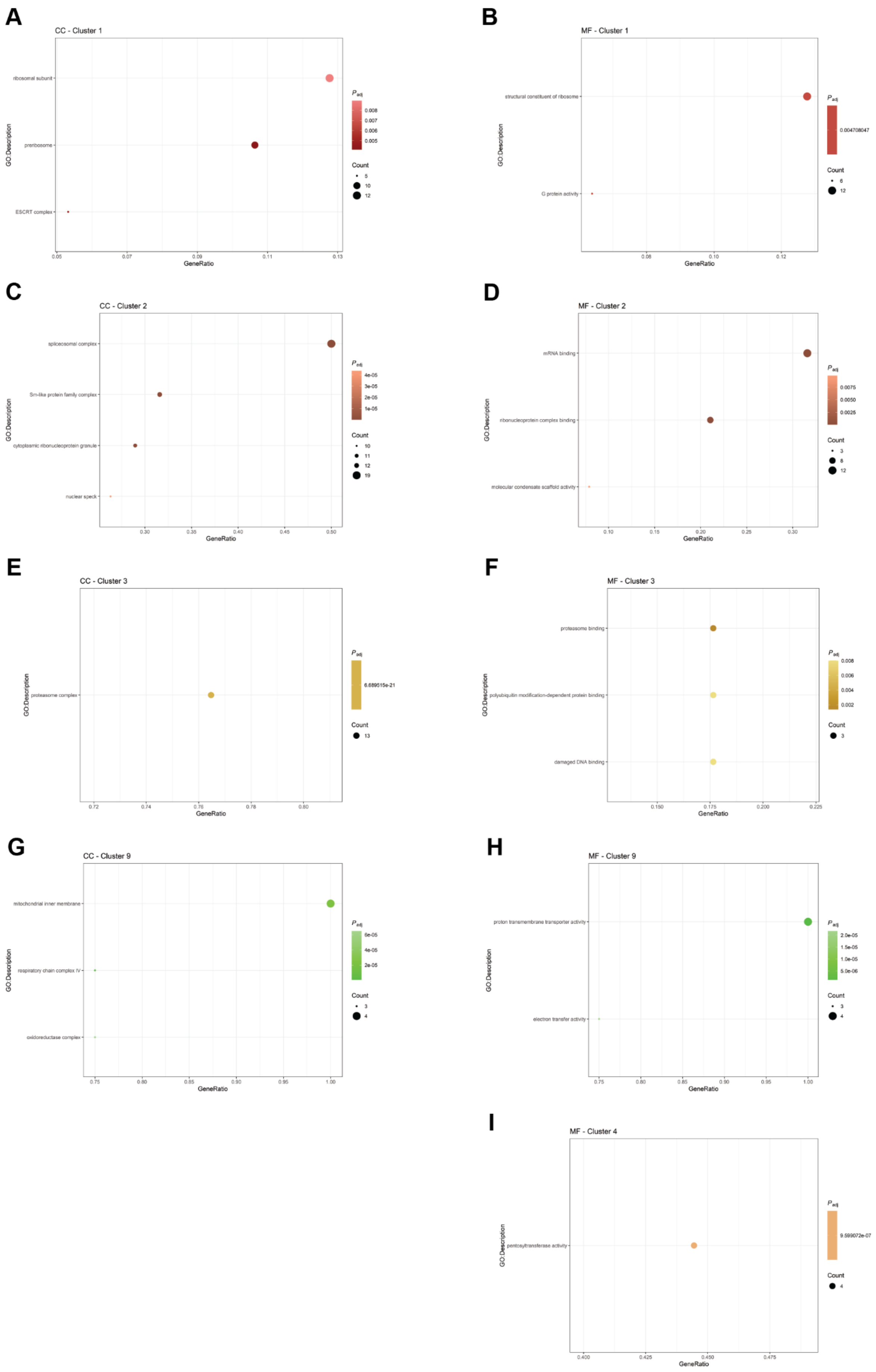
A
CC - Cluster 1
ribosomal subunit
preribosome
ESCRT complex
GO:Description
GeneRatio
P adj
Count
B
MF - Cluster 1
structural constituent of ribosome
G protein activity
0.004708047
C
CC - Cluster 2
spliceosomal complex
Sm-like protein family complex
cytoplasmic ribonucleoprotein granule
nuclear speck
D
MF - Cluster 2
mRNA binding
ribonucleoprotein complex binding
molecular condensate scaffold activity
E
CC - Cluster 3
proteasome complex
6.689515e-21
F
MF - Cluster 3
proteasome binding
polyubiquitin modification-dependent protein binding
damaged DNA binding
G
CC - Cluster 9
mitochondrial inner membrane
respiratory chain complex IV
oxidoreductase complex
H
MF - Cluster 9
proton transmembrane transporter activity
electron transfer activity
I
MF - Cluster 4
pentosyltransferase activity
9.599072e-07

Supplementary Figure S3: The Cellular Component (CC) and Molecular Function (MF) charts, derived from GO analysis of clusters identified via PPI analysis (STRING) from the list of proteins upregulated by 1 mM citicoline (vs. untreated cells, 6h), are presented. Specifically, Panels A and B pertain to Cluster 1; Panels C and D to Cluster 2; and Panels E and F to Cluster 3. Panel G illustrates the Biological Process (BP) of Cluster 4. For clarity and consistency, the color associated with each cluster corresponds to that assigned by the STRING software, as indicated in Supplementary Table 3. Data were filtered using a Benjamini-Hochberg adjusted *p*-value ≤ 0.01. The legend's dots represent the gene count for the specific term identified.

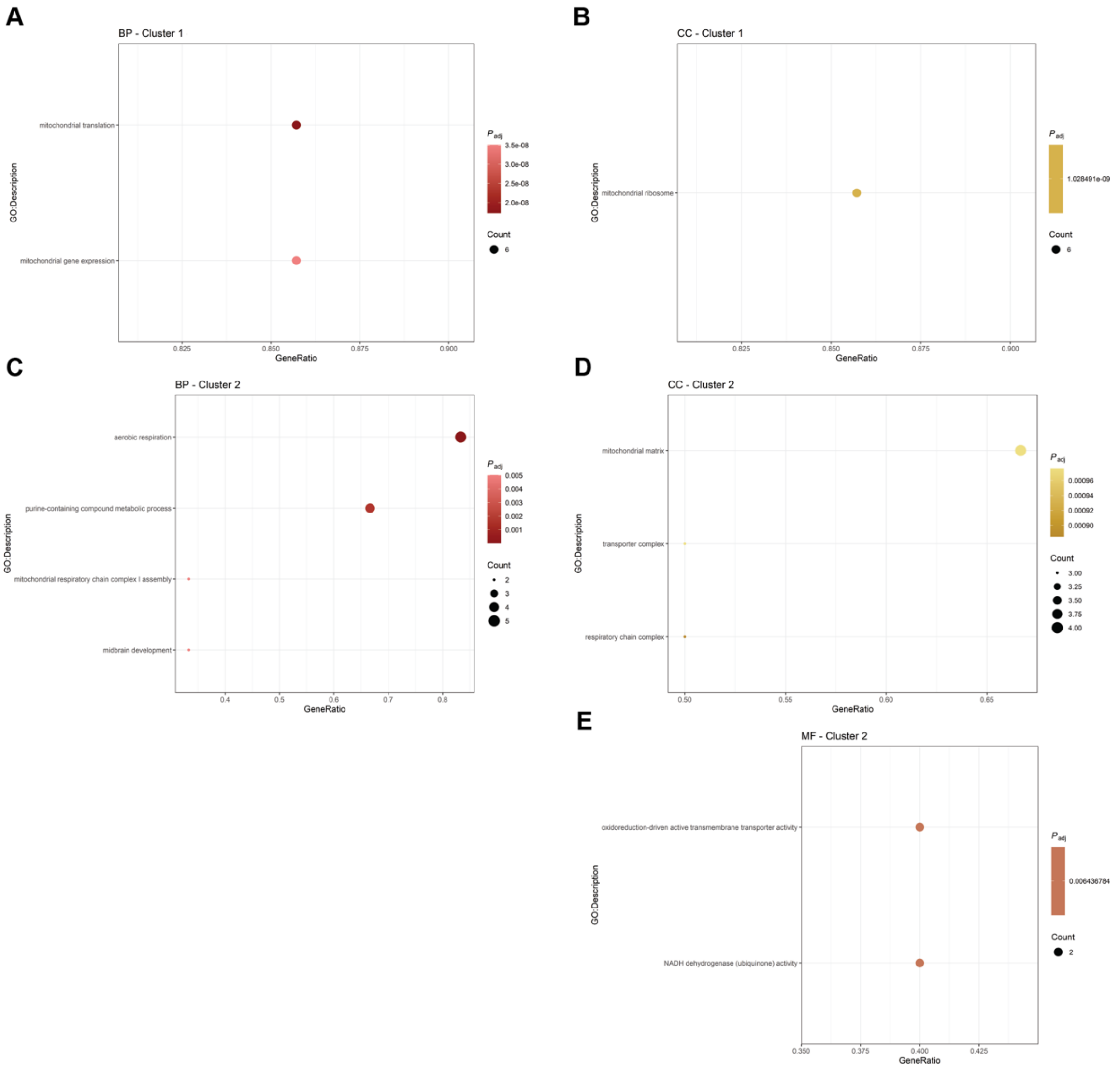

Supplementary Figure S4: The Biological Function (BP), Molecular Function (MF) and Cellular Component (CC) charts, derived from GO analysis of clusters identified as downregulated (1 mM citicoline vs. untreated cells, 6h) via PPI analysis (STRING), are presented.

Specifically, Panels A and B pertain to Cluster 1; Panels C, D and E to Cluster 2. For clarity and consistency, the color associated with each cluster corresponds to that assigned by the STRING software, as indicated in Supplementary Table 3. Data were filtered using a Benjamini-Hochberg adjusted *p*-value ≤ 0.01. The legend's dots represent the gene count for the specific term identified.

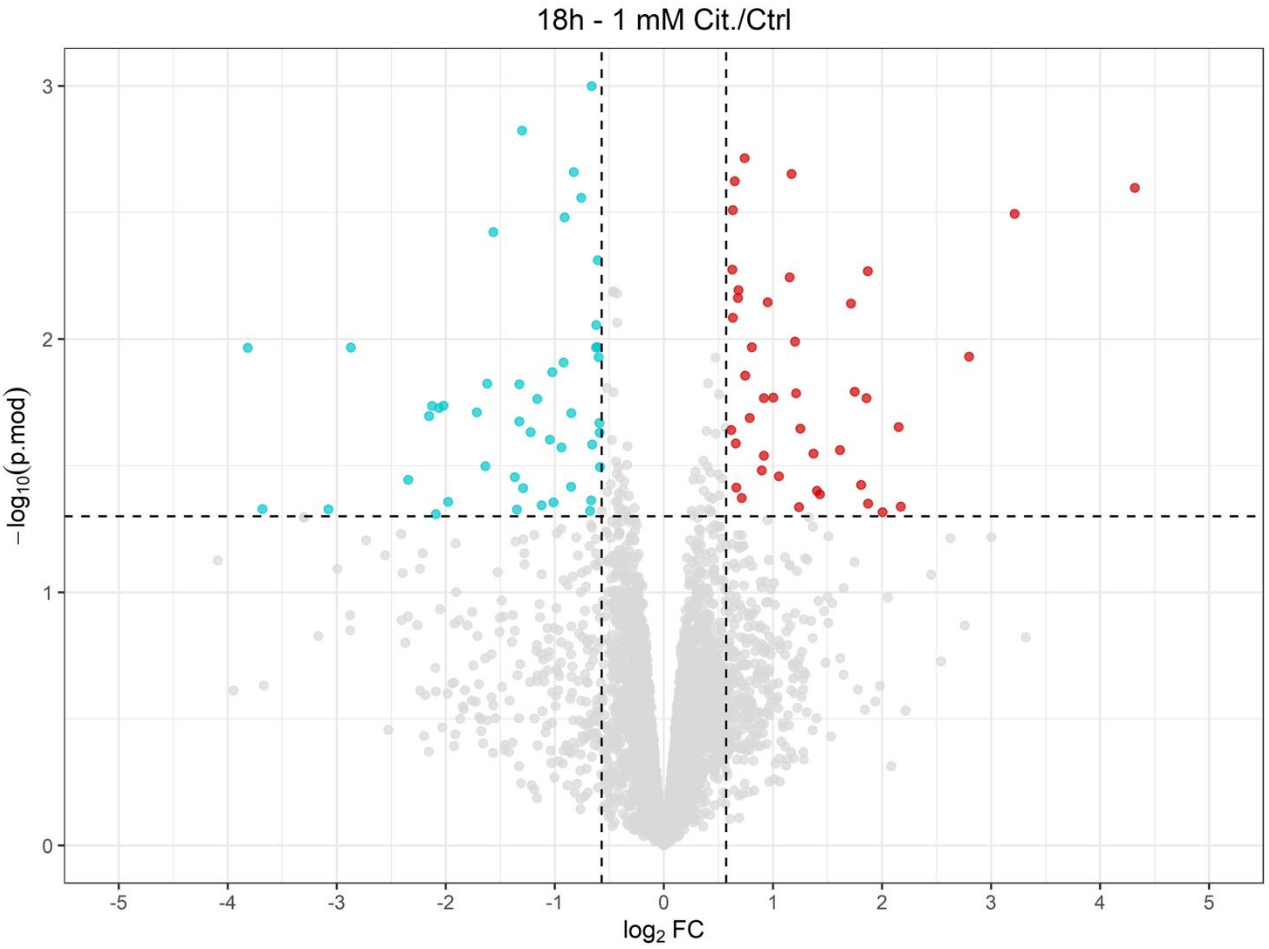

Suppl. Figure S5: Volcano plot showing the distribution of upregulated (red) and downregulated (turquoise) proteins in treated (1 mM citicoline) vs untreated (Ctrl) cells after 18h of stimulation. Dashed lines set the threshold used for labeling proteins as differentially expressed (DEPs). Filters were set for: 0.57≤log_2FC≤0.57 and p≤0.05. This last value is graphed as -log_{10}(p.mod_j) along the y axis.FC:Fold Change.

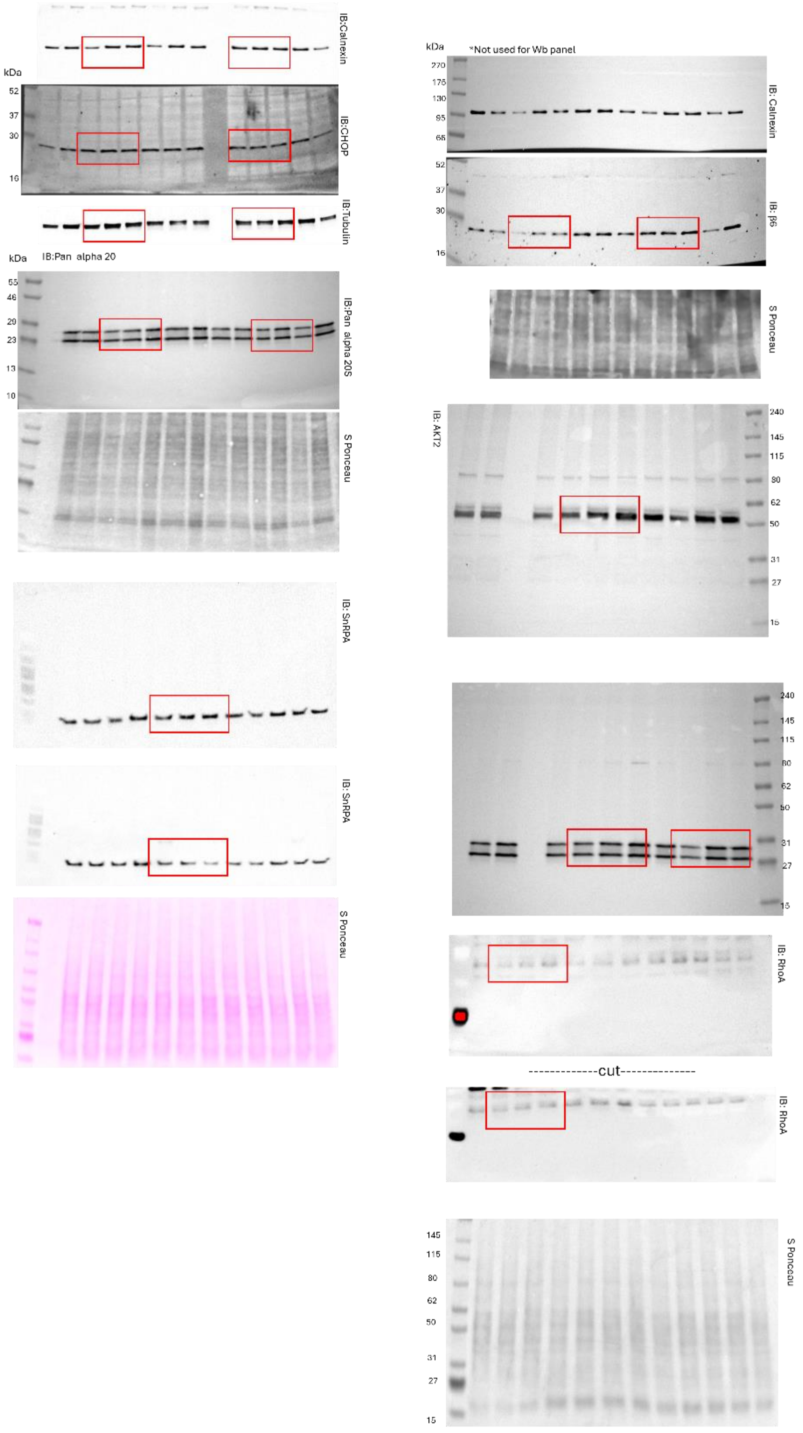

Suppl. Figure S6: Uncropped Western blotting filters used for Wb panel of Figure 8.

SUPPLEMENTARY TABLES

Accession	Description	LogFC	q.mod	Common
**Q9NVH0**	Exonuclease 3'-5' domain-containing protein 2 [OS=Homo sapiens]	4,600355	0,015176	
**O75607**	Nucleoplasmin-3 [OS=Homo sapiens]	4,381182	0,012183	x
**Q8NCF5**	NFATC2-interacting protein [OS=Homo sapiens]	4,130391	0,032327	x
**P13929**	Beta-enolase [OS=Homo sapiens]	3,907201	0,01787	x
**Q9C005**	Protein dpy-30 homolog [OS=Homo sapiens]	3,398399	0,029426	x
**P98179**	RNA-binding protein 3 [OS=Homo sapiens]	3,182229	0,020629	x
**O43156**	TELO2-interacting protein 1 homolog [OS=Homo sapiens]	3,011563	0,006224	x
**Q9NZN3**	EH domain-containing protein 3 [OS=Homo sapiens]	2,910742	0,006224	x
**Q9P2Y4**	Zinc finger protein 219 [OS=Homo sapiens]	2,759812	0,032327	
**Q9BWJ5**	Splicing factor 3B subunit 5 [OS=Homo sapiens]	2,687641	0,039233	
**Q9Y221**	60S ribosome subunit biogenesis protein NIP7 homolog [OS=Homo sapiens]	2,657736	0,021975	x
**P67870**	Casein kinase II subunit beta [OS=Homo sapiens]	2,582722	0,010959	x
**P61224**	Ras-related protein Rap-1b [OS=Homo sapiens]	2,572975	0,01077	x
**P37108**	Signal recognition particle 14 kDa protein [OS=Homo sapiens]	2,55542	0,0349	x
**O14773**	Tripeptidyl-peptidase 1 [OS=Homo sapiens]	2,450363	0,010959	x
**O75635**	Serpin B7 [OS=Homo sapiens]	2,447002	0,032205	x
**P62266**	Small ribosomal subunit protein uS12 [OS=Homo sapiens]	2,359898	0,036217	x
**Q6P158**	Putative ATP-dependent RNA helicase DHX57 [OS=Homo sapiens]	2,340798	0,006224	x
**P49720**	Proteasome subunit beta type-3 [OS=Homo sapiens]	2,260275	0,031215	x

**O15530**	3-phosphoinositide-dependent protein kinase 1 [OS=Homo sapiens]	2,231301	0,006224	x
**P14373**	Zinc finger protein RFP [OS=Homo sapiens]	2,209507	0,050036	
**Q15813**	Tubulin-specific chaperone E [OS=Homo sapiens]	2,187595	0,01077	x
**Q8N6T7**	NAD-dependent protein deacylase sirtuin-6 [OS=Homo sapiens]	2,176706	0,034055	x
**Q15750**	TGF-beta-activated kinase 1 and MAP3K7-binding protein 1 [OS=Homo sapiens]	2,162797	0,015631	
**P82932**	Small ribosomal subunit protein bS6m [OS=Homo sapiens]	2,160572	0,027277	
**P28074**	Proteasome subunit beta type-5 [OS=Homo sapiens]	2,132365	0,015176	x
**Q9H0R6**	Glutamyl-tRNA(Gln) amidotransferase subunit A, mitochondrial [OS=Homo sapiens]	2,108295	0,01077	x
**Q99439**	Calponin-2 [OS=Homo sapiens]	2,07792	0,049996	x
**Q15554**	Telomeric repeat-binding factor 2 [OS=Homo sapiens]	2,027481	0,01006	x
**Q9Y237**	Peptidyl-prolyl cis-trans isomerase NIMA-interacting 4 [OS=Homo sapiens]	2,016671	0,017074	x
**Q15404**	Ras suppressor protein 1 [OS=Homo sapiens]	2,013611	0,029774	x
**P55011**	Solute carrier family 12 member 2 [OS=Homo sapiens]	2,011939	0,035253	x
**Q13867**	Bleomycin hydrolase [OS=Homo sapiens]	2,000649	0,029774	x
**Q2PZI1**	Probable C-mannosyltransferase DPY19L1 [OS=Homo sapiens]	1,993195	0,032205	x
**P12955**	Xaa-Pro dipeptidase [OS=Homo sapiens]	1,974994	0,006224	x
**Q9Y6D5**	Brefeldin A-inhibited guanine nucleotide-exchange protein 2 [OS=Homo sapiens]	1,972531	0,03701	
**Q8TER5**	Rho guanine nucleotide exchange factor 40 [OS=Homo sapiens]	1,955599	0,00866	x
**Q8WUW1**	Protein BRICK1 [OS=Homo sapiens]	1,933748	0,007117	x
**O75530**	Polycomb protein EED [OS=Homo sapiens]	1,930814	0,017113	x
**P43490**	Nicotinamide phosphoribosyltransferase [OS=Homo sapiens]	1,923965	0,014847	x
**Q14004**	Cyclin-dependent kinase 13 [OS=Homo sapiens]	1,923123	0,021767	
**P51114-2**	Isoform 2 of RNA-binding protein FXR1 [OS=Homo sapiens]	1,916706	0,014709	
**P43897**	Elongation factor Ts, mitochondrial [OS=Homo sapiens]	1,890334	0,006224	x
**Q9P0J7**	E3 ubiquitin-protein ligase KCMF1 [OS=Homo sapiens]	1,884361	0,036342	x
**Q6PCB5**	Lysine-specific demethylase RSBN1L [OS=Homo sapiens]	1,876678	0,032123	x
**Q9Y3C8**	Ubiquitin-fold modifier-conjugating enzyme 1 [OS=Homo sapiens]	1,847259	0,014709	x
**Q9H492**	Microtubule-associated proteins 1A/1B light chain 3A [OS=Homo sapiens]	1,8302	0,03953	
**P54725**	UV excision repair protein RAD23 homolog A [OS=Homo sapiens]	1,828788	0,029774	x
**O00422**	Histone deacetylase complex subunit SAP18 [OS=Homo sapiens]	1,823588	0,044898	x
**Q8IV48**	3'-5' exoribonuclease 1 [OS=Homo sapiens]	1,803154	0,029724	

**P00403**	Cytochrome c oxidase subunit 2 [OS=Homo sapiens]	1,788792	0,030874	x
**Q03001**	Dystonin [OS=Homo sapiens]	1,763707	0,00675	x
**P52435**	DNA-directed RNA polymerase II subunit RPB11-a [OS=Homo sapiens]	1,763033	0,010959	x
**Q9NXC5**	GATOR complex protein MIOS [OS=Homo sapiens]	1,745495	0,045199	
**P04350**	Tubulin beta-4A chain [OS=Homo sapiens]	1,744933	0,049996	
**P52888**	Thimet oligopeptidase [OS=Homo sapiens]	1,727512	0,014847	x
**P37802**	Transgelin-2 [OS=Homo sapiens]	1,724634	0,037055	x
**Q9UKN8**	General transcription factor 3C polypeptide 4 [OS=Homo sapiens]	1,721495	0,012817	x
**P49773**	Adenosine 5'-monophosphoramidase HINT1 [OS=Homo sapiens]	1,705205	0,029426	x
**Q12860**	Contactin-1 [OS=Homo sapiens]	1,693342	0,043701	
**Q04637-4**	Isoform C of Eukaryotic translation initiation factor 4 gamma 1 [OS=Homo sapiens]	1,687445	0,027417	x
**P63000**	Ras-related C3 botulinum toxin substrate 1 [OS=Homo sapiens]	1,676476	0,019753	x
**Q6P9B9**	Integrator complex subunit 5 [OS=Homo sapiens]	1,675737	0,020899	x
**Q14376**	UDP-glucose 4-epimerase [OS=Homo sapiens]	1,668127	0,03512	x
**P11233**	Ras-related protein Ral-A [OS=Homo sapiens]	1,666686	0,021073	x
**P20674**	Cytochrome c oxidase subunit 5A, mitochondrial [OS=Homo sapiens]	1,658928	0,014709	x
**O43324**	Eukaryotic translation elongation factor 1 epsilon-1 [OS=Homo sapiens]	1,654415	0,014424	x
**Q8NDT2**	Putative RNA-binding protein 15B [OS=Homo sapiens]	1,63702	0,037744	
**P62269**	Small ribosomal subunit protein uS13 [OS=Homo sapiens]	1,622444	0,031662	x
**Q6GMV3**	Putative peptidyl-tRNA hydrolase PTRHD1 [OS=Homo sapiens]	1,616027	0,042896	
**P54727**	UV excision repair protein RAD23 homolog B [OS=Homo sapiens]	1,589841	0,024171	x
**P09382**	Galectin-1 [OS=Homo sapiens]	1,584804	0,018332	x
**P60981**	Destrin [OS=Homo sapiens]	1,581459	0,019857	x
**P68133**	Actin, alpha skeletal muscle [OS=Homo sapiens]	1,57991	0,016259	x
**Q15022**	Polycomb protein SUZ12 [OS=Homo sapiens]	1,574176	0,014565	x
**Q9H3S7**	Tyrosine-protein phosphatase non-receptor type 23 [OS=Homo sapiens]	1,570987	0,049384	x
**Q9H0H0**	Integrator complex subunit 2 [OS=Homo sapiens]	1,570469	0,014739	x
**Q17RY0**	Cytoplasmic polyadenylation element-binding protein 4 [OS=Homo sapiens]	1,558272	0,006224	x
**Q14781**	Chromobox protein homolog 2 [OS=Homo sapiens]	1,543034	0,01084	x
**Q9HD45**	Transmembrane 9 superfamily member 3 [OS=Homo sapiens]	1,538074	0,00675	x
**Q01081**	Splicing factor U2AF 35 kDa subunit [OS=Homo sapiens]	1,537229	0,007117	x
**Q8IW35**	Centrosomal protein of 97 kDa [OS=Homo sapiens]	1,536586	0,009787	x
**Q9Y2Q5**	Ragulator complex protein LAMTOR2 [OS=Homo sapiens]	1,53014	0,014709	x

**Q15366**	Poly(rC)-binding protein 2 [OS=Homo sapiens]	1,529952	0,031181	x
**Q8IXM2**	Chromatin complexes subunit BAP18 [OS=Homo sapiens]	1,503261	0,010535	x
**P62304**	Small nuclear ribonucleoprotein E [OS=Homo sapiens]	1,495826	0,017756	x
**P24539**	ATP synthase F(0) complex subunit B1, mitochondrial [OS=Homo sapiens]	1,468758	0,028578	
**Q8NHP8**	Putative phospholipase B-like 2 [OS=Homo sapiens]	1,466956	0,030678	x
**P09012**	U1 small nuclear ribonucleoprotein A [OS=Homo sapiens]	1,441052	0,031032	x
**Q9NRX1**	RNA-binding protein PNO1 [OS=Homo sapiens]	1,435643	0,039645	
**O75586**	Mediator of RNA polymerase II transcription subunit 6 [OS=Homo sapiens]	1,427545	0,036311	x
**P39656**	Dolichyl-diphosphooligosaccharide--protein glycosyltransferase 48 kDa subunit [OS=Homo sapiens]	1,421216	0,018682	x
**Q86WJ1**	Chromodomain-helicase-DNA-binding protein 1-like [OS=Homo sapiens]	1,408302	0,012183	x
**Q9Y680**	Peptidyl-prolyl cis-trans isomerase FKBP7 [OS=Homo sapiens]	1,408117	0,012817	x
**O75494**	Serine/arginine-rich splicing factor 10 [OS=Homo sapiens]	1,401935	0,014424	x
**P00505**	Aspartate aminotransferase, mitochondrial [OS=Homo sapiens]	1,396315	0,049661	x
**P25490**	Transcriptional repressor protein YY1 [OS=Homo sapiens]	1,38976	0,026881	x
**Q9P265**	Disco-interacting protein 2 homolog B [OS=Homo sapiens]	1,38264	0,0128	x
**O15145**	Actin-related protein 2/3 complex subunit 3 [OS=Homo sapiens]	1,371242	0,012817	x
**Q8WWN8**	Arf-GAP with Rho-GAP domain, ANK repeat and PH domain-containing protein 3 [OS=Homo sapiens]	1,362582	0,015348	
**A6NHL2**	Tubulin alpha chain-like 3 [OS=Homo sapiens]	1,361581	0,021788	x
**P25325**	3-mercaptopyruvate sulfurtransferase [OS=Homo sapiens]	1,35698	0,02951	x
**Q9NUD5**	Zinc finger CCHC domain-containing protein 3 [OS=Homo sapiens]	1,350514	0,040245	
**Q92609**	TBC1 domain family member 5 [OS=Homo sapiens]	1,347756	0,035357	
**O15173**	Membrane-associated progesterone receptor component 2 [OS=Homo sapiens]	1,347173	0,039239	
**Q9H0U6**	Large ribosomal subunit protein uL18m [OS=Homo sapiens]	1,343246	0,046709	
**Q13509**	Tubulin beta-3 chain [OS=Homo sapiens]	1,33961	0,034911	x
**Q9HB40**	Retinoid-inducible serine carboxypeptidase [OS=Homo sapiens]	1,339572	0,010474	x
**Q9H8Y8**	Golgi reassembly-stacking protein 2 [OS=Homo sapiens]	1,329266	0,014709	x
**P31949**	Protein S100-A11 [OS=Homo sapiens]	1,32598	0,044898	
**Q14978**	Nucleolar and coiled-body phosphoprotein 1 [OS=Homo sapiens]	1,325681	0,014709	x
**O60573**	Eukaryotic translation initiation factor 4E type 2 [OS=Homo sapiens]	1,323199	0,039974	x
**Q9H871**	E3 ubiquitin-protein transferase RMND5A [OS=Homo sapiens]	1,309281	0,037767	

**Q08AM6**	Protein VAC14 homolog [OS=Homo sapiens]	1,294567	0,00675	x
**Q06124**	Tyrosine-protein phosphatase non-receptor type 11 [OS=Homo sapiens]	1,294563	0,033867	x
**P49459**	Ubiquitin-conjugating enzyme E2 A [OS=Homo sapiens]	1,292339	0,010959	x
**Q9UI15**	Transgelin-3 [OS=Homo sapiens]	1,289258	0,010959	x
**Q86WR0**	Coiled-coil domain-containing protein 25 [OS=Homo sapiens]	1,281272	0,010959	
**O95801**	Tetratricopeptide repeat protein 4 [OS=Homo sapiens]	1,280086	0,017173	
**Q5UCC4**	ER membrane protein complex subunit 10 [OS=Homo sapiens]	1,278004	0,00675	x
**P31751**	RAC-beta serine/threonine-protein kinase [OS=Homo sapiens]	1,271907	0,008277	x
**O14964**	Hepatocyte growth factor-regulated tyrosine kinase substrate [OS=Homo sapiens]	1,26791	0,015176	x
**Q6PIS1**	Solute carrier family 23 member 3 [OS=Homo sapiens]	1,259012	0,044724	
**P50416**	Carnitine O-palmitoyltransferase 1, liver isoform [OS=Homo sapiens]	1,257036	0,030874	x
**O43865**	S-adenosylhomocysteine hydrolase-like protein 1 [OS=Homo sapiens]	1,25357	0,032998	
**O00148**	ATP-dependent RNA helicase DDX39A [OS=Homo sapiens]	1,252057	0,014739	x
**P60900**	Proteasome subunit alpha type-6 [OS=Homo sapiens]	1,24092	0,035058	x
**Q96A35**	Large ribosomal subunit protein uL24m [OS=Homo sapiens]	1,238565	0,032516	x
**Q9NRR5**	Ubiquilin-4 [OS=Homo sapiens]	1,237449	0,014739	x
**Q15382**	GTP-binding protein Rheb [OS=Homo sapiens]	1,236587	0,014709	x
**P68402**	Platelet-activating factor acetylhydrolase IB subunit alpha2 [OS=Homo sapiens]	1,22488	0,030717	
**Q96G23**	Ceramide synthase 2 [OS=Homo sapiens]	1,224671	0,037744	x
**Q9NVH1**	DnaJ homolog subfamily C member 11 [OS=Homo sapiens]	1,223664	0,01077	x
**O00743**	Serine/threonine-protein phosphatase 6 catalytic subunit [OS=Homo sapiens]	1,218331	0,029539	x
**Q86U38**	Nucleolar protein 9 [OS=Homo sapiens]	1,21786	0,006698	x
**Q16222**	UDP-N-acetylhexosamine pyrophosphorylase [OS=Homo sapiens]	1,216976	0,014847	x
**Q96SK2**	Transmembrane protein 209 [OS=Homo sapiens]	1,19806	0,037267	
**P61586**	Transforming protein RhoA [OS=Homo sapiens]	1,182642	0,023539	x
**Q9NTJ5**	Phosphatidylinositol-3-phosphatase SAC1 [OS=Homo sapiens]	1,177359	0,031059	x
**P61020**	Ras-related protein Rab-5B [OS=Homo sapiens]	1,156924	0,029774	
**Q9GZQ3**	COMM domain-containing protein 5 [OS=Homo sapiens]	1,152119	0,032327	x
**Q16512**	Serine/threonine-protein kinase N1 [OS=Homo sapiens]	1,149148	0,029774	
**Q9BXI3**	Cytosolic 5'-nucleotidase 1A [OS=Homo sapiens]	1,144558	0,015348	x
**O95376**	E3 ubiquitin-protein ligase ARIH2 [OS=Homo sapiens]	1,133978	0,028894	x
**P28070**	Proteasome subunit beta type-4 [OS=Homo sapiens]	1,132115	0,044493	

**Q9UI10**	Translation initiation factor eIF-2B subunit delta [OS=Homo sapiens]	1,128692	0,00675	x
**P25788**	Proteasome subunit alpha type-3 [OS=Homo sapiens]	1,125038	0,050083	
**Q6UXN9**	WD repeat-containing protein 82 [OS=Homo sapiens]	1,121278	0,021758	x
**P46926**	Glucosamine-6-phosphate isomerase 1 [OS=Homo sapiens]	1,11921	0,011116	x
**Q7Z4V5**	Hepatoma-derived growth factor-related protein 2 [OS=Homo sapiens]	1,118544	0,01787	
**P05026**	Sodium/potassium-transporting ATPase subunit beta-1 [OS=Homo sapiens]	1,114913	0,037912	
**Q8NBL1**	Protein O-glucosyltransferase 1 [OS=Homo sapiens]	1,106024	0,020629	x
**P62910**	Large ribosomal subunit protein eL32 [OS=Homo sapiens]	1,097777	0,014847	x
**P62487**	DNA-directed RNA polymerase II subunit RPB7 [OS=Homo sapiens]	1,089949	0,017113	x
**Q99707**	Methionine synthase [OS=Homo sapiens]	1,088564	0,008793	
**P62879**	Guanine nucleotide-binding protein G(I)/G(S)/G(T) subunit beta-2 [OS=Homo sapiens]	1,083466	0,026304	x
**P62312**	U6 snRNA-associated Sm-like protein LSm6 [OS=Homo sapiens]	1,078056	0,039645	
**P15121**	Aldo-keto reductase family 1 member B1 [OS=Homo sapiens]	1,073033	0,034772	x
**Q2M389**	WASH complex subunit 4 [OS=Homo sapiens]	1,071113	0,015447	x
**P19388**	DNA-directed RNA polymerases I, II, and III subunit RPABC1 [OS=Homo sapiens]	1,066078	0,015176	
**Q05048**	Cleavage stimulation factor subunit 1 [OS=Homo sapiens]	1,060871	0,014709	x
**P84090**	Enhancer of rudimentary homolog [OS=Homo sapiens]	1,057722	0,039645	
**Q6ZNB6**	NF-X1-type zinc finger protein NFXL1 [OS=Homo sapiens]	1,05769	0,037912	
**Q3YEC7**	Rab-like protein 6 [OS=Homo sapiens]	1,056214	0,029539	
**P20618**	Proteasome subunit beta type-1 [OS=Homo sapiens]	1,055958	0,03745	
**Q9BVI4**	Nucleolar complex protein 4 homolog [OS=Homo sapiens]	1,052165	0,039645	x
**P31483**	Cytotoxic granule associated RNA binding protein TIA1 [OS=Homo sapiens]	1,052131	0,04438	
**Q6DD87**	Zinc finger protein 787 [OS=Homo sapiens]	1,050512	0,01787	x
**P53634**	Dipeptidyl peptidase 1 [OS=Homo sapiens]	1,043643	0,014424	x
**Q9UHR6**	Zinc finger HIT domain-containing protein 2 [OS=Homo sapiens]	1,043615	0,04174	x
**Q9Y3E7**	Charged multivesicular body protein 3 [OS=Homo sapiens]	1,042678	0,049614	
**Q6IBS0**	Twinfilin-2 [OS=Homo sapiens]	1,033488	0,014709	x
**P26599-1**	Isoform 2 of Polypyrimidine tract-binding protein 1 [OS=Homo sapiens]	1,028964	0,014424	x
**Q16774**	Guanylate kinase [OS=Homo sapiens]	1,028038	0,047241	x
**Q15018**	BRISC complex subunit Abraxas 2 [OS=Homo sapiens]	1,027714	0,043991	x
**Q9BW27**	Nuclear pore complex protein Nup85 [OS=Homo sapiens]	1,026284	0,014709	x
**P30626**	Sorcin [OS=Homo sapiens]	1,025037	0,032853	x

**Q86WA6**	Valacyclovir hydrolase [OS=Homo sapiens]	1,025034	0,039645	x
**P61371**	Insulin gene enhancer protein ISL-1 [OS=Homo sapiens]	1,019019	0,014847	x
**O60220**	Mitochondrial import inner membrane translocase subunit Tim8 A [OS=Homo sapiens]	1,018848	0,036246	
**Q9NRN7**	L-aminoadipate-semialdehyde dehydrogenase-phosphopantetheinyl transferase [OS=Homo sapiens]	1,013803	0,010668	x
**P12956**	X-ray repair cross-complementing protein 6 [OS=Homo sapiens]	1,012842	0,012817	x
**Q9NPD8**	Ubiquitin-conjugating enzyme E2 T [OS=Homo sapiens]	1,01281	0,029539	x
**P62993**	Growth factor receptor-bound protein 2 [OS=Homo sapiens]	1,008275	0,018428	x
**Q9NQT5**	Exosome complex component RRP40 [OS=Homo sapiens]	1,006777	0,022314	
**Q9UBI1**	COMM domain-containing protein 3 [OS=Homo sapiens]	1,005985	0,048195	x
**Q14746**	Conserved oligomeric Golgi complex subunit 2 [OS=Homo sapiens]	1,005117	0,009787	x
**P01116-2**	Isoform 2B of GTPase KRas [OS=Homo sapiens]	1,003103	0,019697	
**Q9Y3A3**	MOB-like protein phocein [OS=Homo sapiens]	1,00286	0,011116	x
**Q2TAL8**	Transcriptional regulator QRICH1 [OS=Homo sapiens]	1,002355	0,020629	
**P63272**	Transcription elongation factor SPT4 [OS=Homo sapiens]	1,000971	0,044253	
**Q9UMY1**	Nucleolar protein 7 [OS=Homo sapiens]	0,999936	0,031059	x
**Q9BWU0**	Kanadaptin [OS=Homo sapiens]	0,9952	0,014847	x
**Q13148**	TAR DNA-binding protein 43 [OS=Homo sapiens]	0,988423	0,029405	
**P46783**	Small ribosomal subunit protein eS10 [OS=Homo sapiens]	0,986104	0,031998	x
**Q9Y586**	Protein mab-21-like 2 [OS=Homo sapiens]	0,985521	0,048285	
**Q13247**	Serine/arginine-rich splicing factor 6 [OS=Homo sapiens]	0,983076	0,010959	x
**Q92990**	Glomulin [OS=Homo sapiens]	0,98215	0,031059	
**P10768**	S-formylglutathione hydrolase [OS=Homo sapiens]	0,981775	0,034911	x
**P54687**	Branched-chain-amino-acid aminotransferase, cytosolic [OS=Homo sapiens]	0,981625	0,014739	x
**P43363**	Melanoma-associated antigen 10 [OS=Homo sapiens]	0,980274	0,044409	x
**P35080**	Profilin-2 [OS=Homo sapiens]	0,978259	0,029774	x
**Q9ULF5**	Zinc transporter ZIP10 [OS=Homo sapiens]	0,976981	0,050036	
**Q9UHA4**	Ragulator complex protein LAMTOR3 [OS=Homo sapiens]	0,975612	0,009819	x
**P61981**	14-3-3 protein gamma [OS=Homo sapiens]	0,974562	0,017735	
**Q14643**	Inositol 1,4,5-trisphosphate receptor type 1 [OS=Homo sapiens]	0,970076	0,034067	x
**P06730**	Eukaryotic translation initiation factor 4E [OS=Homo sapiens]	0,965789	0,017656	
**Q9UKD2**	mRNA turnover protein 4 homolog [OS=Homo sapiens]	0,965298	0,028879	x

**P12236**	ADP/ATP translocase 3 [OS=Homo sapiens]	0,964566	0,03364	x
**Q9BWW4**	Single-stranded DNA-binding protein 3 [OS=Homo sapiens]	0,96242	0,039077	
**Q6NYC1**	Bifunctional arginine demethylase and lysyl-hydroxylase JMJD6 [OS=Homo sapiens]	0,960855	0,014709	x
**O43447**	Peptidyl-prolyl cis-trans isomerase H [OS=Homo sapiens]	0,95968	0,032327	x
**Q16540**	Large ribosomal subunit protein uL23m [OS=Homo sapiens]	0,953425	0,033535	
**P22061**	Protein-L-isoaspartate(D-aspartate) O-methyltransferase [OS=Homo sapiens]	0,948937	0,019826	x
**P61026**	Ras-related protein Rab-10 [OS=Homo sapiens]	0,94727	0,033535	x
**O43747**	AP-1 complex subunit gamma-1 [OS=Homo sapiens]	0,945888	0,014709	x
**Q9Y478**	5'-AMP-activated protein kinase subunit beta-1 [OS=Homo sapiens]	0,943094	0,029724	
**Q9H3P2**	Negative elongation factor A [OS=Homo sapiens]	0,942624	0,04438	
**P55263**	Adenosine kinase [OS=Homo sapiens]	0,941748	0,037055	x
**Q6PJ69**	E3 ubiquitin-protein ligase TRIM65 [OS=Homo sapiens]	0,936132	0,021517	
**Q6UWP7**	Lysocardiolipin acyltransferase 1 [OS=Homo sapiens]	0,927327	0,029774	x
**P13984**	General transcription factor IIF subunit 2 [OS=Homo sapiens]	0,910517	0,015513	x
**P10619**	Lysosomal protective protein [OS=Homo sapiens]	0,909968	0,014709	x
**Q13315**	Serine-protein kinase ATM [OS=Homo sapiens]	0,909554	0,016141	
**Q96SU4**	Oxysterol-binding protein-related protein 9 [OS=Homo sapiens]	0,909152	0,027745	
**O43281**	Embryonal Fyn-associated substrate [OS=Homo sapiens]	0,906928	0,021975	x
**P14678**	Small nuclear ribonucleoprotein-associated proteins B and B' [OS=Homo sapiens]	0,904578	0,032205	x
**Q9BW85**	Splicing factor YJU2 [OS=Homo sapiens]	0,904298	0,033535	
**P40426**	Pre-B-cell leukemia transcription factor 3 [OS=Homo sapiens]	0,898572	0,021975	
**Q10567-3**	Isoform C of AP-1 complex subunit beta-1 [OS=Homo sapiens]	0,898015	0,010959	x
**P50897**	Palmitoyl-protein thioesterase 1 [OS=Homo sapiens]	0,895817	0,015176	x
**O00767**	Stearoyl-CoA desaturase [OS=Homo sapiens]	0,894325	0,02592	x
**P12814**	Alpha-actinin-1 [OS=Homo sapiens]	0,892179	0,021767	x
**P30086**	Phosphatidylethanolamine-binding protein 1 [OS=Homo sapiens]	0,888837	0,01787	x
**Q9UGP8**	Translocation protein SEC63 homolog [OS=Homo sapiens]	0,886515	0,027959	
**P22626**	Heterogeneous nuclear ribonucleoproteins A2/B1 [OS=Homo sapiens]	0,886131	0,010668	x
**Q15061**	WD repeat-containing protein 43 [OS=Homo sapiens]	0,884898	0,014739	x
**Q9H3N1**	Thioredoxin-related transmembrane protein 1 [OS=Homo sapiens]	0,883971	0,010975	x
**Q9Y2H6**	Fibronectin type-III domain-containing protein 3A [OS=Homo sapiens]	0,883335	0,0364	

**P62841**	Small ribosomal subunit protein uS19 [OS=Homo sapiens]	0,878483	0,014847	
**Q8TC12**	Retinol dehydrogenase 11 [OS=Homo sapiens]	0,872492	0,014709	x
**P62308**	Small nuclear ribonucleoprotein G [OS=Homo sapiens]	0,870718	0,048047	x
**Q96DB5**	Regulator of microtubule dynamics protein 1 [OS=Homo sapiens]	0,869663	0,019697	
**Q9NUJ3**	T-complex protein 11-like protein 1 [OS=Homo sapiens]	0,864089	0,042394	
**Q8IXH7**	Negative elongation factor C/D [OS=Homo sapiens]	0,862945	0,031853	
**Q04760**	Lactoylglutathione lyase [OS=Homo sapiens]	0,862829	0,019753	x
**Q9BUF5**	Tubulin beta-6 chain [OS=Homo sapiens]	0,862729	0,044498	
**Q16186**	Proteasomal ubiquitin receptor ADRM1 [OS=Homo sapiens]	0,86221	0,014739	
**P60174**	Triosephosphate isomerase [OS=Homo sapiens]	0,861331	0,02592	x
**O60888**	Protein CutA [OS=Homo sapiens]	0,855259	0,037744	
**Q13572**	Inositol-tetrakisphosphate 1-kinase [OS=Homo sapiens]	0,854624	0,039814	x
**P25789**	Proteasome subunit alpha type-4 [OS=Homo sapiens]	0,851634	0,02494	
**P60510**	Serine/threonine-protein phosphatase 4 catalytic subunit [OS=Homo sapiens]	0,846152	0,021975	
**O43159**	Ribosomal RNA-processing protein 8 [OS=Homo sapiens]	0,844377	0,032839	
**O95630**	STAM-binding protein [OS=Homo sapiens]	0,843049	0,027479	
**Q7Z4Q2**	HEAT repeat-containing protein 3 [OS=Homo sapiens]	0,841905	0,036191	
**O75884**	Serine hydrolase RBBP9 [OS=Homo sapiens]	0,84166	0,03395	
**Q13283**	Ras GTPase-activating protein-binding protein 1 [OS=Homo sapiens]	0,840929	0,014709	x
**Q9Y3U8**	Large ribosomal subunit protein eL36 [OS=Homo sapiens]	0,840884	0,029426	x
**P07948**	Tyrosine-protein kinase Lyn [OS=Homo sapiens]	0,840395	0,032264	x
**Q9NZL4**	Hsp70-binding protein 1 [OS=Homo sapiens]	0,83787	0,014709	
**P26368**	Splicing factor U2AF 65 kDa subunit [OS=Homo sapiens]	0,837636	0,015176	x
**Q9Y4B6**	DDB1- and CUL4-associated factor 1 [OS=Homo sapiens]	0,825382	0,010959	x
**Q9UKV8**	Protein argonaute-2 [OS=Homo sapiens]	0,821883	0,025758	x
**P14174**	Macrophage migration inhibitory factor [OS=Homo sapiens]	0,820471	0,012168	x
**Q9NRF8**	CTP synthase 2 [OS=Homo sapiens]	0,81746	0,014424	x
**Q9H0E2**	Toll-interacting protein [OS=Homo sapiens]	0,816752	0,01787	x
**Q8N543**	Prolyl 3-hydroxylase OGFOD1 [OS=Homo sapiens]	0,81101	0,02951	
**Q96D46**	60S ribosomal export protein NMD3 [OS=Homo sapiens]	0,81065	0,015176	x
**Q15738**	Sterol-4-alpha-carboxylate 3-dehydrogenase, decarboxylating [OS=Homo sapiens]	0,809723	0,036217	x
**Q9BUN8**	Derlin-1 [OS=Homo sapiens]	0,809219	0,026309	x
**O00625**	Pirin [OS=Homo sapiens]	0,803721	0,015348	x

**P49770**	Translation initiation factor eIF-2B subunit beta [OS=Homo sapiens]	0,802046	0,014709	x
**Q71U36**	Tubulin alpha-1A chain [OS=Homo sapiens]	0,801669	0,03395	
**O75718**	Cartilage-associated protein [OS=Homo sapiens]	0,801624	0,033535	x
**P11279**	Lysosome-associated membrane glycoprotein 1 [OS=Homo sapiens]	0,800711	0,045686	
**P56937**	3-keto-steroid reductase/17-beta-hydroxysteroid dehydrogenase 7 [OS=Homo sapiens]	0,799115	0,04964	
**Q9ULC4**	Malignant T-cell-amplified sequence 1 [OS=Homo sapiens]	0,794681	0,01077	x
**P56730**	Neurotrypsin [OS=Homo sapiens]	0,793366	0,02494	
**P47224**	Guanine nucleotide exchange factor MSS4 [OS=Homo sapiens]	0,792457	0,011635	x
**Q16539**	Mitogen-activated protein kinase 14 [OS=Homo sapiens]	0,790977	0,014847	x
**Q9NVC6**	Mediator of RNA polymerase II transcription subunit 17 [OS=Homo sapiens]	0,789308	0,032205	
**Q15526**	Surfeit locus protein 1 [OS=Homo sapiens]	0,789278	0,014709	
**Q9NR09**	Baculoviral IAP repeat-containing protein 6 [OS=Homo sapiens]	0,785622	0,036191	x
**Q63HM9**	PI-PLC X domain-containing protein 3 [OS=Homo sapiens]	0,783421	0,021455	x
**Q9UHY1**	Nuclear receptor-binding protein [OS=Homo sapiens]	0,780995	0,014847	
**P23771**	Trans-acting T-cell-specific transcription factor GATA-3 [OS=Homo sapiens]	0,780723	0,037055	
**Q9H706**	GRB2-associated and regulator of MAPK protein 1 [OS=Homo sapiens]	0,7757	0,037658	
**Q96FZ7**	Charged multivesicular body protein 6 [OS=Homo sapiens]	0,773183	0,044498	x
**P00387**	NADH-cytochrome b5 reductase 3 [OS=Homo sapiens]	0,769632	0,032806	
**Q9H9H4**	Vacuolar protein sorting-associated protein 37B [OS=Homo sapiens]	0,768265	0,041565	
**P61313**	Large ribosomal subunit protein eL15 [OS=Homo sapiens]	0,766809	0,020899	x
**Q9UET6**	Putative tRNA (cytidine(32)/guanosine(34)-2'-O)-methyltransferase [OS=Homo sapiens]	0,765889	0,021758	
**Q9Y3B2**	Exosome complex component CSL4 [OS=Homo sapiens]	0,762883	0,031854	x
**P30405**	Peptidyl-prolyl cis-trans isomerase F, mitochondrial [OS=Homo sapiens]	0,761884	0,029426	x
**Q5T6V5**	Queuosine 5'-phosphate N-glycosylase/hydrolase [OS=Homo sapiens]	0,761265	0,03728	x
**Q96BR5**	Cytochrome c oxidase assembly factor 7 [OS=Homo sapiens]	0,76124	0,041544	x
**Q5VTL8**	Pre-mRNA-splicing factor 38B [OS=Homo sapiens]	0,759431	0,035253	x
**Q13595**	Transformer-2 protein homolog alpha [OS=Homo sapiens]	0,758637	0,019697	
**Q9BPX5**	Actin-related protein 2/3 complex subunit 5-like protein [OS=Homo sapiens]	0,757531	0,04787	
**Q9BZK7**	F-box-like/WD repeat-containing protein TBL1XR1 [OS=Homo sapiens]	0,755889	0,018502	

**P67775**	Serine/threonine-protein phosphatase 2A catalytic subunit alpha isoform [OS=Homo sapiens]	0,753799	0,015176	x
**Q9Y305**	Acyl-coenzyme A thioesterase 9, mitochondrial [OS=Homo sapiens]	0,75025	0,032806	
**P52298**	Nuclear cap-binding protein subunit 2 [OS=Homo sapiens]	0,749356	0,029426	
**P61970**	Nuclear transport factor 2 [OS=Homo sapiens]	0,748483	0,039645	x
**Q8N1F8**	Serine/threonine-protein kinase 11-interacting protein [OS=Homo sapiens]	0,746659	0,037055	
**O95372**	Acyl-protein thioesterase 2 [OS=Homo sapiens]	0,744708	0,04612	
**Q96RE7**	Nucleus accumbens-associated protein 1 [OS=Homo sapiens]	0,743962	0,014847	
**Q13907**	Isopentenyl-diphosphate Delta-isomerase 1 [OS=Homo sapiens]	0,742315	0,035058	
**Q8TEX9**	Importin-4 [OS=Homo sapiens]	0,742055	0,011954	x
**Q08380**	Galectin-3-binding protein [OS=Homo sapiens]	0,74086	0,036191	
**P00338**	L-lactate dehydrogenase A chain [OS=Homo sapiens]	0,739738	0,010959	x
**O75396**	Vesicle-trafficking protein SEC22b [OS=Homo sapiens]	0,738242	0,037543	
**Q9HC52**	Chromobox protein homolog 8 [OS=Homo sapiens]	0,737943	0,014739	x
**Q9BQ67**	Glutamate-rich WD repeat-containing protein 1 [OS=Homo sapiens]	0,73545	0,014865	x
**Q99816**	Tumor susceptibility gene 101 protein [OS=Homo sapiens]	0,734878	0,01787	x
**P61163**	Alpha-centractin [OS=Homo sapiens]	0,734726	0,039233	
**O43252**	Bifunctional 3'-phosphoadenosine 5'-phosphosulfate synthase 1 [OS=Homo sapiens]	0,730073	0,014336	
**Q9NY27**	Serine/threonine-protein phosphatase 4 regulatory subunit 2 [OS=Homo sapiens]	0,729358	0,027417	
**O43598**	2'-deoxynucleoside 5'-phosphate N-hydrolase 1 [OS=Homo sapiens]	0,728607	0,01214	x
**O43813**	Glutathione S-transferase LANCL1 [OS=Homo sapiens]	0,728416	0,011954	
**P30566**	Adenylosuccinate lyase [OS=Homo sapiens]	0,725512	0,02951	x
**Q16775**	Hydroxyacylglutathione hydrolase, mitochondrial [OS=Homo sapiens]	0,723002	0,028578	x
**Q01968**	Inositol polyphosphate 5-phosphatase OCRL [OS=Homo sapiens]	0,721824	0,036755	
**Q99829**	Copine-1 [OS=Homo sapiens]	0,716433	0,036311	
**Q13885**	Tubulin beta-2A chain [OS=Homo sapiens]	0,715741	0,027203	
**Q9P287**	BRCA2 and CDKN1A-interacting protein [OS=Homo sapiens]	0,714501	0,015176	
**P14854**	Cytochrome c oxidase subunit 6B1 [OS=Homo sapiens]	0,711158	0,048194	
**Q01581**	Hydroxymethylglutaryl-CoA synthase, cytoplasmic [OS=Homo sapiens]	0,711136	0,048463	
**Q9Y2X7**	ARF GTPase-activating protein GIT1 [OS=Homo sapiens]	0,710588	0,036952	
**P57081**	tRNA (guanine-N(7)-)-methyltransferase non-catalytic subunit WDR4 [OS=Homo sapiens]	0,710471	0,017756	
**Q16881**	Thioredoxin reductase 1, cytoplasmic [OS=Homo sapiens]	0,709964	0,021767	

**P07437**	Tubulin beta chain [OS=Homo sapiens]	0,707818	0,029774	
**Q9UBF8**	Phosphatidylinositol 4-kinase beta [OS=Homo sapiens]	0,704927	0,027417	x
**O00330**	Pyruvate dehydrogenase protein X component, mitochondrial [OS=Homo sapiens]	0,703888	0,02592	
**P62318**	Small nuclear ribonucleoprotein Sm D3 [OS=Homo sapiens]	0,703133	0,014847	x
**Q9Y5L0-1**	Isoform 1 of Transportin-3 [OS=Homo sapiens]	0,699785	0,036191	x
**Q6PJG6**	BRCA1-associated ATM activator 1 [OS=Homo sapiens]	0,69751	0,021975	
**Q8NB90**	ATPase family gene 2 protein homolog A [OS=Homo sapiens]	0,695708	0,01772	x
**P26641**	Elongation factor 1-gamma [OS=Homo sapiens]	0,695292	0,029774	x
**P25787**	Proteasome subunit alpha type-2 [OS=Homo sapiens]	0,692579	0,02592	
**P49366**	Deoxyhypusine synthase [OS=Homo sapiens]	0,692215	0,023539	x
**P62314**	Small nuclear ribonucleoprotein Sm D1 [OS=Homo sapiens]	0,689769	0,039645	
**Q92900**	Regulator of nonsense transcripts 1 [OS=Homo sapiens]	0,689523	0,032666	
**Q9GZN8**	Adipose-secreted signaling protein [OS=Homo sapiens]	0,68942	0,035787	
**O43592**	Exportin-T [OS=Homo sapiens]	0,689401	0,014709	
**Q8N6N3**	UPF0690 protein C1orf52 [OS=Homo sapiens]	0,687875	0,020899	x
**Q9H074**	Polyadenylate-binding protein-interacting protein 1 [OS=Homo sapiens]	0,687384	0,013423	
**P37198**	Nuclear pore glycoprotein p62 [OS=Homo sapiens]	0,685321	0,043141	
**Q8IYD1**	Eukaryotic peptide chain release factor GTP-binding subunit ERF3B [OS=Homo sapiens]	0,684514	0,027088	x
**Q9H7N4**	Splicing factor, arginine/serine-rich 19 [OS=Homo sapiens]	0,681873	0,032205	
**Q96IU4**	Putative protein-lysine deacylase ABHD14B [OS=Homo sapiens]	0,680099	0,020899	
**Q9H832**	Ubiquitin-conjugating enzyme E2 Z [OS=Homo sapiens]	0,679504	0,01787	x
**P08579**	U2 small nuclear ribonucleoprotein B'' [OS=Homo sapiens]	0,678857	0,014709	
**Q96AX1**	Vacuolar protein sorting-associated protein 33A [OS=Homo sapiens]	0,678161	0,017113	x
**P22694**	cAMP-dependent protein kinase catalytic subunit beta [OS=Homo sapiens]	0,676747	0,032327	x
**Q86W42**	THO complex subunit 6 homolog [OS=Homo sapiens]	0,675186	0,034055	
**Q96EK6**	Glucosamine 6-phosphate N-acetyltransferase [OS=Homo sapiens]	0,671498	0,012817	
**Q99536**	Synaptic vesicle membrane protein VAT-1 homolog [OS=Homo sapiens]	0,66439	0,021299	
**P55265**	Double-stranded RNA-specific adenosine deaminase [OS=Homo sapiens]	0,663619	0,019941	
**Q9Y6J9**	TAF6-like RNA polymerase II p300/CBP-associated factor-associated factor 65 kDa subunit 6L [OS=Homo sapiens]	0,661586	0,027959	x

**Q13325**	Interferon-induced protein with tetratricopeptide repeats 5 [OS=Homo sapiens]	0,657587	0,049996	
**Q92879**	CUGBP Elav-like family member 1 [OS=Homo sapiens]	0,655036	0,011635	
**P83731**	Large ribosomal subunit protein eL24 [OS=Homo sapiens]	0,65496	0,037543	
**Q9H4A6**	Golgi phosphoprotein 3 [OS=Homo sapiens]	0,653232	0,013423	
**Q9Y5J1**	U3 small nucleolar RNA-associated protein 18 homolog [OS=Homo sapiens]	0,651126	0,021975	
**Q96AG4**	Leucine-rich repeat-containing protein 59 [OS=Homo sapiens]	0,643142	0,048213	
**Q9BTD8**	RNA-binding protein 42 [OS=Homo sapiens]	0,643097	0,014847	
**Q96EP5**	DAZ-associated protein 1 [OS=Homo sapiens]	0,642919	0,01772	
**P60891**	Ribose-phosphate pyrophosphokinase 1 [OS=Homo sapiens]	0,642566	0,025638	
**P07384**	Calpain-1 catalytic subunit [OS=Homo sapiens]	0,642446	0,017074	
**Q9BWD1**	Acetyl-CoA acetyltransferase, cytosolic [OS=Homo sapiens]	0,642148	0,02592	x
**P11940**	Polyadenylate-binding protein 1 [OS=Homo sapiens]	0,641694	0,014847	
**P04899**	Guanine nucleotide-binding protein G(i) subunit alpha-2 [OS=Homo sapiens]	0,640225	0,02494	
**Q9NY93**	Probable ATP-dependent RNA helicase DDX56 [OS=Homo sapiens]	0,637596	0,032205	
**Q9GZR2**	RNA exonuclease 4 [OS=Homo sapiens]	0,636853	0,027745	x
**Q13310**	Polyadenylate-binding protein 4 [OS=Homo sapiens]	0,636163	0,014739	
**Q9HBM1**	Kinetochore protein Spc25 [OS=Homo sapiens]	0,636073	0,050036	
**Q99470**	Stromal cell-derived factor 2 [OS=Homo sapiens]	0,634545	0,021975	
**O95104**	SR-related and CTD-associated factor 4 [OS=Homo sapiens]	0,633678	0,019598	
**Q15102**	Platelet-activating factor acetylhydrolase IB subunit alpha1 [OS=Homo sapiens]	0,632181	0,019697	
**Q15796**	Mothers against decapentaplegic homolog 2 [OS=Homo sapiens]	0,626917	0,049424	x
**Q02809**	Procollagen-lysine,2-oxoglutarate 5-dioxygenase 1 [OS=Homo sapiens]	0,626569	0,033202	x
**P00568**	Adenylate kinase isoenzyme 1 [OS=Homo sapiens]	0,623706	0,032839	x
**P49721**	Proteasome subunit beta type-2 [OS=Homo sapiens]	0,621239	0,020692	
**Q99719**	Septin-5 [OS=Homo sapiens]	0,614094	0,01787	
**Q969X6**	U3 small nucleolar RNA-associated protein 4 homolog [OS=Homo sapiens]	0,614028	0,050036	
**P00492**	Hypoxanthine-guanine phosphoribosyltransferase [OS=Homo sapiens]	0,609647	0,014227	
**Q7Z5L9**	Interferon regulatory factor 2-binding protein 2 [OS=Homo sapiens]	0,607289	0,032205	
**Q08945**	FACT complex subunit SSRP1 [OS=Homo sapiens]	0,603961	0,027745	
**Q96A65**	Exocyst complex component 4 [OS=Homo sapiens]	0,60391	0,02113	x
**Q9UIG0**	Tyrosine-protein kinase BAZ1B [OS=Homo sapiens]	0,601716	0,014709	

**P30519**	Heme oxygenase 2 [OS=Homo sapiens]	0,597671	0,029426	
**Q9Y617**	Phosphoserine aminotransferase [OS=Homo sapiens]	0,596951	0,02777	
**P60763**	Ras-related C3 botulinum toxin substrate 3 [OS=Homo sapiens]	0,595428	0,02494	
**Q9H944**	Mediator of RNA polymerase II transcription subunit 20 [OS=Homo sapiens]	0,593465	0,028774	
**Q9P000**	COMM domain-containing protein 9 [OS=Homo sapiens]	0,593253	0,021767	x
**P00491**	Purine nucleoside phosphorylase [OS=Homo sapiens]	0,592179	0,039645	
**Q96PU8**	KH domain-containing RNA-binding protein QKI [OS=Homo sapiens]	0,585831	0,049301	
**Q8TAF3**	WD repeat-containing protein 48 [OS=Homo sapiens]	0,582876	0,02113	
**O00764**	Pyridoxal kinase [OS=Homo sapiens]	0,58265	0,043757	
**P27824**	Calnexin [OS=Homo sapiens]	0,579846	0,031059	
**Q9Y5N6**	Origin recognition complex subunit 6 [OS=Homo sapiens]	0,57921	0,037658	x
**Q8WVK2**	U4/U6.U5 small nuclear ribonucleoprotein 27 kDa protein [OS=Homo sapiens]	0,578185	0,035787	
**O75367**	Core histone macro-H2A.1 [OS=Homo sapiens]	0,577955	0,017074	
**P68036**	Ubiquitin-conjugating enzyme E2 L3 [OS=Homo sapiens]	0,575731	0,014709	
**Q49AR2**	UPF0489 protein C5orf22 [OS=Homo sapiens]	0,575297	0,039873	
**P46736**	Lys-63-specific deubiquitinase BRCC36 [OS=Homo sapiens]	0,574254	0,02951	
**P35222**	Catenin beta-1 [OS=Homo sapiens]	0,573906	0,039645	
**Q68EM7**	Rho GTPase-activating protein 17 [OS=Homo sapiens]	0,571184	0,031811	
**Q9H2M9**	Rab3 GTPase-activating protein non-catalytic subunit [OS=Homo sapiens]	0,570282	0,043141	x
**P38919**	Eukaryotic initiation factor 4A-III [OS=Homo sapiens]	0,5702	0,044409	

Supplementary Table S1: Reported in this table are the proteins upregulated by 1 mM citicoline (compared to untreated cells) after 6 hours. For each entry, the Uniprot Accession number, protein description, log2FC, and FDR (*q.mod*) from the Limma test are provided. Column labeled as Common identifies (where “x” is present) proteins found upregulated also in the presence of 0.1 mM citicoline.

Accession	Description	LogFC	q.mod	Common
**Q6P4R8**	Nuclear factor related to kappa-B-binding protein [OS=Homo sapiens]	-5,08599	0,048583	
**Q5VZM2**	Ras-related GTP-binding protein B [OS=Homo sapiens]	-4,32163	0,043617	x
**P12235**	ADP/ATP translocase 1 [OS=Homo sapiens]	-4,11598	0,019753	
**O14949**	Cytochrome b-c1 complex subunit 8 [OS=Homo sapiens]	-3,95342	0,010959	x
**Q96DX5**	Ankyrin repeat and SOCS box protein 9 [OS=Homo sapiens]	-3,73494	0,00675	x
**Q96GN5**	Cell division cycle-associated 7-like protein [OS=Homo sapiens]	-3,32197	0,018153	
**Q9NQS1**	Cell death regulator Aven [OS=Homo sapiens]	-3,23175	0,013423	x
**Q9BVW5**	TIMELESS-interacting protein [OS=Homo sapiens]	-3,21373	0,01772	x
**P02656**	Apolipoprotein C-III [OS=Homo sapiens]	-3,21086	0,015176	x
**Q9UHD2**	Serine/threonine-protein kinase TBK1 [OS=Homo sapiens]	-3,1849	0,031059	x
**Q8IXI2**	Mitochondrial Rho GTPase 1 [OS=Homo sapiens]	-3,11339	0,019387	x
**Q9HBH5**	Retinol dehydrogenase 14 [OS=Homo sapiens]	-3,04263	0,006224	x
**Q12981-1**	Isoform 3 of Vesicle transport protein SEC20 [OS=Homo sapiens]	-2,9092	0,010959	x
**Q99880**	Histone H2B type 1-L [OS=Homo sapiens]	-2,84449	0,014336	
**Q9GZN1**	Actin-related protein 6 [OS=Homo sapiens]	-2,83898	0,015302	
**Q05084**	Islet cell autoantigen 1 [OS=Homo sapiens]	-2,82443	0,019621	
**Q9UIJ7**	GTP:AMP phosphotransferase AK3, mitochondrial [OS=Homo sapiens]	-2,81914	0,035293	

**Q5JPH6**	Probable glutamate--tRNA ligase, mitochondrial [OS=Homo sapiens]	-2,7456	0,049301	x
**Q9NVR2**	Integrator complex subunit 10 [OS=Homo sapiens]	- 2,57763	0,014739	
**Q5VWZ2**	Lysophospholipase-like protein 1 [OS=Homo sapiens]	- 2,49943	0,048937	x
**Q6ZN04**	RNA-binding protein MEX3B [OS=Homo sapiens]	- 2,49581	0,016641	
**Q14978-3**	Isoform 3 of Nucleolar and coiled-body phosphoprotein 1 [OS=Homo sapiens]	- 2,48385	0,031059	x
**Q15043**	Metal cation symporter ZIP14 [OS=Homo sapiens]	- 2,47244	0,006224	
**Q8IUR0**	Trafficking protein particle complex subunit 5 [OS=Homo sapiens]	- 2,41251	0,039645	
**Q8NI60**	Atypical kinase COQ8A, mitochondrial [OS=Homo sapiens]	- 2,33583	0,046897	
**Q7L0J3**	Synaptic vesicle glycoprotein 2A [OS=Homo sapiens]	- 2,33448	0,019387	x
**P82914**	Small ribosomal subunit protein uS15m [OS=Homo sapiens]	- 2,32561	0,049796	
**P82673**	Small ribosomal subunit protein mS35 [OS=Homo sapiens]	- 2,26066	0,006224	x
**Q9BSF4**	Mitochondrial import inner membrane translocase subunit Tim29 [OS=Homo sapiens]	- 2,22777	0,034911	x
**P78318**	Immunoglobulin-binding protein 1 [OS=Homo sapiens]	- 2,19066	0,015176	x
**Q13686**	Nucleic acid dioxygenase ALKBH1 [OS=Homo sapiens]	- 2,17261	0,01077	x
**Q8WV22**	Non-structural maintenance of chromosomes element 1 homolog [OS=Homo sapiens]	- 2,09887	0,018428	x
**Q8NBN7**	Retinol dehydrogenase 13 [OS=Homo sapiens]	- 2,08333	0,032327	x
**P35610**	Sterol O-acyltransferase 1 [OS=Homo sapiens]	- 2,06445	0,010668	x
**Q9P0P0**	E3 ubiquitin-protein ligase RNF181 [OS=Homo sapiens]	- 2,04901	0,03666	
**Q8N9N5**	Protein BANP [OS=Homo sapiens]	- 2,04199	0,006224	
**Q8NHG8**	E3 ubiquitin-protein ligase ZNRF2 [OS=Homo sapiens]	- 2,04014	0,011217	x
**Q8NBF6**	Late secretory pathway protein AVL9 homolog [OS=Homo sapiens]	- 2,02022	0,036854	
**Q9BYB4**	Guanine nucleotide-binding protein subunit beta-like protein 1 [OS=Homo sapiens]	- 1,97458	0,01006	x
**Q9BT25**	HAUS augmin-like complex subunit 8 [OS=Homo sapiens]	- 1,96609	0,014739	x
**P24385**	G1/S-specific cyclin-D1 [OS=Homo sapiens]	- 1,87324	0,010959	x
**Q8N8A6**	ATP-dependent RNA helicase DDX51 [OS=Homo sapiens]	- 1,83889	0,036952	x
**Q13641**	Trophoblast glycoprotein [OS=Homo sapiens]	- 1,79688	0,014709	x
**P21579**	Synaptotagmin-1 [OS=Homo sapiens]	-1,7822	0,014709	x
**P43694**	Transcription factor GATA-4 [OS=Homo sapiens]	- 1,74743	0,032327	
**Q9H920**	E3 ubiquitin ligase RNF121 [OS=Homo sapiens]	- 1,74425	0,046518	x
**P23511**	Nuclear transcription factor Y subunit alpha [OS=Homo sapiens]	- 1,70224	0,021176	x

**P46108-2**	Isoform Crk-I of Adapter molecule crk [OS=Homo sapiens]	-1,6672	0,020225	x
**O96005**	Putative lipid scramblase CLPTM1 [OS=Homo sapiens]	-1,64785	0,045753	
**P50750**	Cyclin-dependent kinase 9 [OS=Homo sapiens]	-1,64594	0,015176	x
**Q8IYI6**	Exocyst complex component 8 [OS=Homo sapiens]	-1,60472	0,029426	
**Q9BUA3**	Spindlin interactor and repressor of chromatin-binding protein [OS=Homo sapiens]	-1,52062	0,006224	x
**Q14562**	ATP-dependent RNA helicase DHX8 [OS=Homo sapiens]	-1,50705	0,016737	x
**Q96IW2**	SH2 domain-containing adapter protein D [OS=Homo sapiens]	-1,4914	0,01077	x
**O94964**	Protein SOGA1 [OS=Homo sapiens]	-1,47076	0,012817	x
**Q96CS2**	HAUS augmin-like complex subunit 1 [OS=Homo sapiens]	-1,45551	0,018997	x
**Q96I51**	RCC1-like G exchanging factor-like protein [OS=Homo sapiens]	-1,43958	0,032205	
**Q7LGA3**	Heparan sulfate 2-O-sulfotransferase 1 [OS=Homo sapiens]	-1,36513	0,048595	
**Q15345**	Leucine-rich repeat-containing protein 41 [OS=Homo sapiens]	-1,35775	0,009243	x
**Q86X55**	Histone-arginine methyltransferase CARM1 [OS=Homo sapiens]	-1,34645	0,014739	x
**Q96DG6**	Carboxymethylenebutenolidase homolog [OS=Homo sapiens]	-1,33411	0,010959	x
**Q9GZY8**	Mitochondrial fission factor [OS=Homo sapiens]	-1,32465	0,013423	x
**Q15643**	Thyroid receptor-interacting protein 11 [OS=Homo sapiens]	-1,32018	0,032806	x
**Q3KQV9**	UDP-N-acetylhexosamine pyrophosphorylase-like protein 1 [OS=Homo sapiens]	-1,31905	0,015578	x
**P50583**	Bis(5'-nucleosyl)-tetraphosphatase [asymmetrical] [OS=Homo sapiens]	-1,30028	0,025758	x
**Q9HAV7**	GrpE protein homolog 1, mitochondrial [OS=Homo sapiens]	-1,2989	0,014739	x
**Q9Y663**	Heparan sulfate glucosamine 3-O-sulfotransferase 3A1 [OS=Homo sapiens]	-1,28494	0,039233	
**Q0VGL1**	Ragulator complex protein LAMTOR4 [OS=Homo sapiens]	-1,2799	0,006224	x
**Q9H3H9**	Transcription elongation factor A protein-like 2 [OS=Homo sapiens]	-1,27442	0,016535	x
**Q68D10**	Protein SPT2 homolog [OS=Homo sapiens]	-1,25773	0,027479	
**Q15031**	Leucine--tRNA ligase, mitochondrial [OS=Homo sapiens]	-1,24142	0,038833	
**Q99615**	DnaJ homolog subfamily C member 7 [OS=Homo sapiens]	-1,22921	0,027745	x
**Q8N668**	COMM domain-containing protein 1 [OS=Homo sapiens]	-1,21465	0,046026	
**Q9UKG1**	DCC-interacting protein 13-alpha [OS=Homo sapiens]	-1,20131	0,017074	x
**Q9NYB0**	Telomeric repeat-binding factor 2-interacting protein 1 [OS=Homo sapiens]	-1,18446	0,010959	
**P49356**	Protein farnesyltransferase subunit beta [OS=Homo sapiens]	-1,16925	0,01772	
**O60936**	Nucleolar protein 3 [OS=Homo sapiens]	-1,15846	0,02494	

**Q9NVZ3**	Adaptin ear-binding coat-associated protein 2 [OS=Homo sapiens]	-1,13251	0,006698	x
**Q9ULP9**	TBC1 domain family member 24 [OS=Homo sapiens]	-1,13001	0,02951	
**Q9H7X7**	Intraflagellar transport protein 22 homolog [OS=Homo sapiens]	-1,11978	0,029774	x
**Q969X5**	Endoplasmic reticulum-Golgi intermediate compartment protein 1 [OS=Homo sapiens]	-1,11005	0,021299	x
**Q6ZSR9**	Uncharacterized protein FLJ45252 [OS=Homo sapiens]	-1,08607	0,02592	
**Q86VM9**	Zinc finger CCCH domain-containing protein 18 [OS=Homo sapiens]	-1,0839	0,020899	x
**Q9Y6Y0**	Influenza virus NS1A-binding protein [OS=Homo sapiens]	-1,079	0,046352	x
**Q5JRX3**	Presequence protease, mitochondrial [OS=Homo sapiens]	-1,05037	0,029426	x
**P61599**	N-alpha-acetyltransferase 20 [OS=Homo sapiens]	-1,04561	0,025663	
**Q86UE4**	Protein LYRIC [OS=Homo sapiens]	-1,04023	0,018682	x
**Q6ZUT1**	Uncharacterized protein NKAPD1 [OS=Homo sapiens]	-1,0288	0,019753	x
**Q68CQ4**	U3 small nucleolar RNA-associated protein 25 homolog [OS=Homo sapiens]	-1,01035	0,017074	
**O15155**	BET1 homolog [OS=Homo sapiens]	-1,00304	0,02065	x
**Q96P70**	Importin-9 [OS=Homo sapiens]	-1,00085	0,029426	x
**Q13503**	Mediator of RNA polymerase II transcription subunit 21 [OS=Homo sapiens]	-0,98729	0,044903	
**Q9H6V9**	Lipid droplet-associated hydrolase [OS=Homo sapiens]	-0,9839	0,017656	x
**P22466**	Galanin peptides [OS=Homo sapiens]	-0,9784	0,015176	x
**Q9H900**	Protein zwilch homolog [OS=Homo sapiens]	-0,97649	0,041101	x
**P62714**	Serine/threonine-protein phosphatase 2A catalytic subunit beta isoform [OS=Homo sapiens]	-0,96588	0,009819	
**Q96AY4**	Tetratricopeptide repeat protein 28 [OS=Homo sapiens]	-0,96305	0,019753	x
**Q9GZZ1**	N-alpha-acetyltransferase 50 [OS=Homo sapiens]	-0,94402	0,019697	x
**Q8IY37**	Probable ATP-dependent RNA helicase DHX37 [OS=Homo sapiens]	-0,93321	0,031936	x
**P12036**	Neurofilament heavy polypeptide [OS=Homo sapiens]	-0,92588	0,030964	x
**Q5JTZ9**	Alanine--tRNA ligase, mitochondrial [OS=Homo sapiens]	-0,91897	0,014709	x
**Q8N6M0**	Deubiquitinase OTUD6B [OS=Homo sapiens]	-0,91175	0,048047	
**Q8NB46**	Serine/threonine-protein phosphatase 6 regulatory ankyrin repeat subunit C [OS=Homo sapiens]	-0,9069	0,03728	
**P51649**	Succinate-semialdehyde dehydrogenase, mitochondrial [OS=Homo sapiens]	-0,90517	0,040886	x
**Q16740**	ATP-dependent Clp protease proteolytic subunit, mitochondrial [OS=Homo sapiens]	-0,89557	0,02494	x

**O75879**	Glutamyl-tRNA(Gln) amidotransferase subunit B, mitochondrial [OS=Homo sapiens]	-0,89523	0,044253	
**Q13136**	Liprin-alpha-1 [OS=Homo sapiens]	-0,89482	0,010668	x
**Q96EK5**	KIF-binding protein [OS=Homo sapiens]	-0,89273	0,017995	
**O75616**	GTPase Era, mitochondrial [OS=Homo sapiens]	-0,88968	0,037109	
**Q96CB9**	5-methylcytosine rRNA methyltransferase NSUN4 [OS=Homo sapiens]	-0,88687	0,014229	
**O95926**	Pre-mRNA-splicing factor SYF2 [OS=Homo sapiens]	-0,88529	0,014847	x
**Q8IUD2**	ELKS/Rab6-interacting/CAST family member 1 [OS=Homo sapiens]	-0,87727	0,031561	
**O43615**	Mitochondrial import inner membrane translocase subunit TIM44 [OS=Homo sapiens]	-0,87307	0,046616	
**P22681**	E3 ubiquitin-protein ligase CBL [OS=Homo sapiens]	-0,86612	0,02951	
**Q5UIP0**	Telomere-associated protein RIF1 [OS=Homo sapiens]	-0,86261	0,015835	x
**P63027**	Vesicle-associated membrane protein 2 [OS=Homo sapiens]	-0,8577	0,021299	
**Q96HE7**	ERO1-like protein alpha [OS=Homo sapiens]	-0,8507	0,021767	x
**Q99549**	M-phase phosphoprotein 8 [OS=Homo sapiens]	-0,84305	0,037267	
**Q567U6**	Coiled-coil domain-containing protein 93 [OS=Homo sapiens]	-0,84267	0,044253	x
**Q92481**	Transcription factor AP-2-beta [OS=Homo sapiens]	-0,83433	0,029426	x
**Q13394**	Putative nucleotidyltransferase MAB21L1 [OS=Homo sapiens]	-0,83053	0,021975	
**Q6N069**	N-alpha-acetyltransferase 16, NatA auxiliary subunit [OS=Homo sapiens]	-0,82325	0,01901	x
**Q8NDX6**	Zinc finger protein 740 [OS=Homo sapiens]	-0,82247	0,014847	x
**Q32P41**	tRNA (guanine(37)-N1)-methyltransferase [OS=Homo sapiens]	-0,81894	0,011635	
**O15061**	Synemin [OS=Homo sapiens]	-0,81551	0,019857	
**Q9H9L3**	Interferon-stimulated 20 kDa exonuclease-like 2 [OS=Homo sapiens]	-0,8123	0,035511	
**Q7Z2W4**	Zinc finger CCCH-type antiviral protein 1 [OS=Homo sapiens]	-0,8113	0,015176	x
**Q9H2F5**	Enhancer of polycomb homolog 1 [OS=Homo sapiens]	-0,81039	0,041101	
**Q13405**	Large ribosomal subunit protein mL49 [OS=Homo sapiens]	-0,8102	0,01901	x
**Q9Y4X5**	E3 ubiquitin-protein ligase ARIH1 [OS=Homo sapiens]	-0,80985	0,036282	x
**P51452**	Dual specificity protein phosphatase 3 [OS=Homo sapiens]	-0,80626	0,013342	x
**Q86YR5**	G-protein-signaling modulator 1 [OS=Homo sapiens]	-0,80335	0,03953	
**Q9Y5S2**	Serine/threonine-protein kinase MRCK beta [OS=Homo sapiens]	-0,79803	0,01772	
**P30084**	Enoyl-CoA hydratase, mitochondrial [OS=Homo sapiens]	-0,78108	0,019472	

**P78362**	SRSF protein kinase 2 [OS=Homo sapiens]	-0,7758	0,014805	
**Q9Y6I3**	Epsin-1 [OS=Homo sapiens]	-0,77491	0,019673	x
**Q9UBP0**	Spastin [OS=Homo sapiens]	-0,77042	0,046026	
**Q9Y676**	Small ribosomal subunit protein mS40 [OS=Homo sapiens]	-0,75637	0,029173	
**Q14657**	EKC/KEOPS complex subunit LAGE3 [OS=Homo sapiens]	-0,75236	0,047241	x
**P53367**	Arfaptin-1 [OS=Homo sapiens]	-0,75042	0,034055	
**Q9UJZ1**	Stomatin-like protein 2, mitochondrial [OS=Homo sapiens]	-0,74861	0,032327	
**Q9BYD1**	Large ribosomal subunit protein uL13m [OS=Homo sapiens]	-0,74853	0,050036	
**Q7Z4G1**	COMM domain-containing protein 6 [OS=Homo sapiens]	-0,7354	0,032839	x
**P00367**	Glutamate dehydrogenase 1, mitochondrial [OS=Homo sapiens]	-0,72649	0,019941	x
**Q8TAT6**	Nuclear protein localization protein 4 homolog [OS=Homo sapiens]	-0,72636	0,030164	x
**Q9UBQ6**	Exostosin-like 2 [OS=Homo sapiens]	-0,72499	0,027479	x
**Q7L5D6**	Golgi to ER traffic protein 4 homolog [OS=Homo sapiens]	-0,72242	0,04849	
**P04062**	Lysosomal acid glucosylceramidase [OS=Homo sapiens]	-0,72001	0,037055	
**Q9BS26**	Endoplasmic reticulum resident protein 44 [OS=Homo sapiens]	-0,71455	0,029426	
**Q86X76**	Deaminated glutathione amidase [OS=Homo sapiens]	-0,7031	0,014847	
**Q9Y6A5**	Transforming acidic coiled-coil-containing protein 3 [OS=Homo sapiens]	-0,6998	0,01787	x
**Q9Y6M9**	NADH dehydrogenase [ubiquinone] 1 beta subcomplex subunit 9 [OS=Homo sapiens]	-0,69927	0,017074	
**P53597**	Succinate--CoA ligase [ADP/GDP-forming] subunit alpha, mitochondrial [OS=Homo sapiens]	-0,69811	0,029426	
**P0DPB6**	DNA-directed RNA polymerases I and III subunit RPAC2 [OS=Homo sapiens]	-0,69683	0,039645	
**Q96F63**	Coiled-coil domain-containing protein 97 [OS=Homo sapiens]	-0,69585	0,050036	
**P60484**	Phosphatidylinositol 3,4,5-trisphosphate 3-phosphatase and dual-specificity protein phosphatase PTEN [OS=Homo sapiens]	-0,69046	0,029426	
**P22033**	Methylmalonyl-CoA mutase, mitochondrial [OS=Homo sapiens]	-0,68223	0,023509	
**P43155**	Carnitine O-acetyltransferase [OS=Homo sapiens]	-0,67626	0,046717	
**O00139**	Kinesin-like protein KIF2A [OS=Homo sapiens]	-0,67408	0,04521	
**Q96RS6**	NudC domain-containing protein 1 [OS=Homo sapiens]	-0,67118	0,044498	
**Q14671**	Pumilio homolog 1 [OS=Homo sapiens]	-0,66257	0,042394	
**Q9UBS4**	DnaJ homolog subfamily B member 11 [OS=Homo sapiens]	-0,66227	0,035979	x
**P40425**	Pre-B-cell leukemia transcription factor 2 [OS=Homo sapiens]	-0,66116	0,037867	x

**Q9Y285**	Phenylalanine--tRNA ligase alpha subunit [OS=Homo sapiens]	-0,65689	0,011954	
**Q9Y2E8**	Sodium/hydrogen exchanger 8 [OS=Homo sapiens]	-0,65638	0,027959	
**Q86YP4**	Transcriptional repressor p66-alpha [OS=Homo sapiens]	-0,65618	0,027959	
**Q07021**	Complement component 1 Q subcomponent-binding protein, mitochondrial [OS=Homo sapiens]	-0,6527	0,042924	x
**Q09666**	Neuroblast differentiation-associated protein AHNAK [OS=Homo sapiens]	-0,64932	0,04	
**Q01658**	Protein Dr1 [OS=Homo sapiens]	-0,64731	0,018732	x
**Q8IVL6**	Prolyl 3-hydroxylase 3 [OS=Homo sapiens]	-0,64486	0,034652	
**Q13207**	T-box transcription factor TBX2 [OS=Homo sapiens]	-0,63877	0,046518	x
**P29084**	Transcription initiation factor IIE subunit beta [OS=Homo sapiens]	-0,63828	0,02494	
**Q9H1A4**	Anaphase-promoting complex subunit 1 [OS=Homo sapiens]	-0,63807	0,031736	
**Q9UBW8**	COP9 signalosome complex subunit 7a [OS=Homo sapiens]	-0,63663	0,039645	
**Q9UQR0**	Sex comb on midleg-like protein 2 [OS=Homo sapiens]	-0,62864	0,039233	x
**P27816**	Microtubule-associated protein 4 [OS=Homo sapiens]	-0,6286	0,020852	
**Q9Y5K6**	CD2-associated protein [OS=Homo sapiens]	-0,62518	0,019826	
**Q92665**	Small ribosomal subunit protein mS31 [OS=Homo sapiens]	-0,62085	0,029426	x
**Q14527**	Helicase-like transcription factor [OS=Homo sapiens]	-0,61823	0,01772	
**O60885**	Bromodomain-containing protein 4 [OS=Homo sapiens]	-0,61762	0,031059	x
**Q96SI9**	Spermatid perinuclear RNA-binding protein [OS=Homo sapiens]	-0,61387	0,029724	
**P26885**	Peptidyl-prolyl cis-trans isomerase FKBP2 [OS=Homo sapiens]	-0,61009	0,031059	
**Q9P2E9**	Ribosome-binding protein 1 [OS=Homo sapiens]	-0,60192	0,03512	
**P82909**	Alpha-ketoglutarate dehydrogenase component 4 [OS=Homo sapiens]	-0,60144	0,024309	
**O75489**	NADH dehydrogenase [ubiquinone] iron-sulfur protein 3, mitochondrial [OS=Homo sapiens]	-0,60108	0,046245	x
**Q8NEZ5**	F-box only protein 22 [OS=Homo sapiens]	-0,59548	0,046245	
**Q6SZW1**	NAD(+) hydrolase SARM1 [OS=Homo sapiens]	-0,59298	0,029584	
**Q7Z7N9**	Transmembrane protein 179B [OS=Homo sapiens]	-0,59296	0,046245	x
**Q8WXF7**	Atlastin-1 [OS=Homo sapiens]	-0,59139	0,031059	
**Q1KMD3**	Heterogeneous nuclear ribonucleoprotein U-like protein 2 [OS=Homo sapiens]	-0,58989	0,04202	
**Q9Y448**	Small kinetochore-associated protein [OS=Homo sapiens]	-0,58267	0,03953	
**Q99798**	Aconitate hydratase, mitochondrial [OS=Homo sapiens]	-0,58264	0,034067	

**P38117**	Electron transfer flavoprotein subunit beta [OS=Homo sapiens]	-0,57369	0,049582	

Supplementary Table S2: Reported in this table are the proteins downregulated by 1 mM citicoline (compared to untreated cells) after 6 hours. For each entry, the Uniprot Accession number, protein description, log2FC, and FDR (*q.mod*) from the Limma test are provided. Column labeled as Common identifies (where "x" is present) proteins found downregulated also in the presence of 0.1 mM citicoline.

Clustering Method	Cluster Number	Cluster Color	Hex Color	Hex Color	Protein Name	Protein Identifier	Protein Description
kmeans	1	Red	#ff0000	94	ACTA1	9606.ENSP00000355645	Actin, alpha skeletal muscle, intermediate form; Actins are highly conserved proteins that are involved in various types of cell motility and are ubiquitously expressed in all eukaryotic cells; Belongs to the actin family.
kmeans	1	Red	#ff0000	94	AHCYL1	9606.ENSP00000358814	S-adenosylhomocysteine hydrolase-like protein 1; Multifaceted cellular regulator which coordinates several essential cellular functions including regulation of epithelial HCO3(-) and fluid secretion, mRNA processing and DNA replication. Regulates ITPR1 sensitivity to inositol 1,4,5-trisphosphate competing for the common binding site and acting as endogenous 'pseudoligand' whose inhibitory activity can be modulated by its phosphorylation status. In the pancreatic and salivary ducts, at resting state, attenuates inositol 1,4,5-trisphosphate-induced calcium release by interacting with ITP [...]
kmeans	1	Red	#ff0000	94	AKT2	9606.ENSP00000375892	RAC-beta serine/threonine-protein kinase; AKT2 is one of 3 closely related serine/threonine-protein kinases (AKT1, AKT2 and AKT3) called the AKT kinase, and which regulate many processes including metabolism, proliferation, cell survival, growth and angiogenesis. This is mediated through serine and/or threonine phosphorylation of a range of downstream substrates. Over 100

							substrate candidates have been reported so far, but for most of them, no isoform specificity has been reported. AKT is responsible of the regulation of glucose uptake by mediating insulin-induced translocation of the [...]
kmeans	1	Red	#ff0000	94	ARAP3	9606.ENSP00000239440	Arf-GAP with Rho-GAP domain, ANK repeat and PH domain-containing protein 3; Phosphatidylinositol 3,4,5-trisphosphate-dependent GTPase-activating protein that modulates actin cytoskeleton remodeling by regulating ARF and RHO family members. Is activated by phosphatidylinositol 3,4,5-trisphosphate (PtdIns(3,4,5)P3) binding. Can be activated by phosphatidylinositol 3,4-bisphosphate (PtdIns(3,4,5)P2) binding, albeit with lower efficiency. Acts on ARF6, RAC1, RHOA and CDC42. Plays a role in the internalization of anthrax toxin.
kmeans	1	Red	#ff0000	94	ARPC3	9606.ENSP00000228825	Actin-related protein 2/3 complex subunit 3; Component of the Arp2/3 complex, a multiprotein complex that mediates actin polymerization upon stimulation by nucleation-promoting factor (NPF). The Arp2/3 complex mediates the formation of branched actin networks in the cytoplasm, providing the force for cell motility. In addition to its role in the cytoplasmic cytoskeleton, the Arp2/3 complex also promotes actin polymerization in the nucleus, thereby regulating gene transcription and repair of damaged DNA. The Arp2/3 complex promotes homologous recombination (HR) repair in response to DNA [...]
kmeans	1	Red	#ff0000	94	ARPC5L	9606.ENSP00000345361	Actin-related protein 2/3 complex subunit 5-like protein; May function as component of the Arp2/3 complex which is involved in regulation of actin polymerization and together with an activating nucleation-promoting factor (NPF) mediates the formation of branched actin networks; Belongs to the ARPC5 family.
kmeans	1	Red	#ff0000	94	BCAT1	9606.ENSP00000443459	Branched-chain-amino-acid aminotransferase, cytosolic; Catalyzes the first reaction in the catabolism of the essential branched chain amino acids leucine, isoleucine, and valine.
kmeans	1	Red	#ff0000	94	BRK1	9606.ENSP00000432472	Protein BRICK1; Involved in regulation of actin and microtubule organization. Part of a WAVE complex that activates the Arp2/3 complex. As component of the WAVE1 complex, required for BDNF-NTRK2 endocytic trafficking and signaling from early endosomes (By similarity).

kmeans	1	Red	#ff0000	94	CHMP3	9606.ENSP00000263856	Charged multivesicular body protein 3; Probable core component of the endosomal sorting required for transport complex III (ESCRT-III) which is involved in multivesicular bodies (MVBs) formation and sorting of endosomal cargo proteins into MVBs. MVBs contain intraluminal vesicles (ILVs) that are generated by invagination and scission from the limiting membrane of the endosome and mostly are delivered to lysosomes enabling degradation of membrane proteins, such as stimulated growth factor receptors, lysosomal enzymes and lipids. The MVB pathway appears to require the sequential function [...]
kmeans	1	Red	#ff0000	94	CHMP6	9606.ENSP00000317468	Charged multivesicular body protein 6; Probable core component of the endosomal sorting required for transport complex III (ESCRT-III) which is involved in multivesicular bodies (MVBs) formation and sorting of endosomal cargo proteins into MVBs. MVBs contain intraluminal vesicles (ILVs) that are generated by invagination and scission from the limiting membrane of the endosome and mostly are delivered to lysosomes enabling degradation of membrane proteins, such as stimulated growth factor receptors, lysosomal enzymes and lipids. The MVB pathway appears to require the sequential function [...]
kmeans	1	Red	#ff0000	94	CSNK2B	9606.ENSP00000365042	Casein kinase II subunit beta; Participates in Wnt signaling (By similarity). Plays a complex role in regulating the basal catalytic activity of the alpha subunit; Belongs to the casein kinase 2 subunit beta family.
kmeans	1	Red	#ff0000	94	CTNNB1	9606.ENSP00000495360	Catenin beta-1; Key downstream component of the canonical Wnt signaling pathway. In the absence of Wnt, forms a complex with AXIN1, AXIN2, APC, CSNK1A1 and GSK3B that promotes phosphorylation on N-terminal Ser and Thr residues and ubiquitination of CTNNB1 via BTRC and its subsequent degradation by the proteasome. In the presence of Wnt ligand, CTNNB1 is not ubiquitinated and accumulates in the nucleus, where it acts as a coactivator for transcription factors of the TCF/LEF family, leading to activate Wnt responsive genes. Involved in the regulation of cell adhesion, as component of an [...]
kmeans	1	Red	#ff0000	94	DDX56	9606.ENSP00000258772	Probable ATP-dependent RNA helicase DDX56; May play a role in later stages of the processing of the pre- ribosomal particles leading to mature 60S ribosomal subunits. Has intrinsic ATPase activity; Belongs to the DEAD box helicase family. DDX56/DBP9 subfamily.
kmeans	1	Red	#ff0000	94	EEF1G	9606.ENSP00000331901	Elongation factor 1-gamma; Probably plays a role in anchoring the complex to other cellular components.

kmeans	1	Red	#ff0000	94	ENO3	9606.ENSP00000324105	Beta-enolase; Appears to have a function in striated muscle development and regeneration; Belongs to the enolase family.
kmeans	1	Red	#ff0000	94	EXOC4	9606.ENSP00000253861	Exocyst complex component 4; Component of the exocyst complex involved in the docking of exocytic vesicles with fusion sites on the plasma membrane.
kmeans	1	Red	#ff0000	94	EXOSC1	9606.ENSP00000359939	Exosome complex component CSL4; Non-catalytic component of the RNA exosome complex which has 3'->5' exoribonuclease activity and participates in a multitude of cellular RNA processing and degradation events. In the nucleus, the RNA exosome complex is involved in proper maturation of stable RNA species such as rRNA, snRNA and snoRNA, in the elimination of RNA processing by-products and non-coding 'pervasive' transcripts, such as antisense RNA species and promoter-upstream transcripts (PROMPTs), and of mRNAs with processing defects, thereby limiting or excluding their export to the cytop [...]
kmeans	1	Red	#ff0000	94	EXOSC3	9606.ENSP00000323046	Exosome complex component RRP40; Non-catalytic component of the RNA exosome complex which has 3'->5' exoribonuclease activity and participates in a multitude of cellular RNA processing and degradation events. In the nucleus, the RNA exosome complex is involved in proper maturation of stable RNA species such as rRNA, snRNA and snoRNA, in the elimination of RNA processing by-products and non-coding 'pervasive' transcripts, such as antisense RNA species and promoter-upstream transcripts (PROMPTs), and of mRNAs with processing defects, thereby limiting or excluding their export to the cyto [...]
kmeans	1	Red	#ff0000	94	GAREM1	9606.ENSP00000269209	GRB2-associated and regulator of MAPK protein 1; [Isoform 1]: Acts as an adapter protein that plays a role in intracellular signaling cascades triggered either by the cell surface activated epidermal growth factor receptor and/or cytoplasmic protein tyrosine kinases. Promotes activation of the MAPK/ERK signaling pathway. Plays a role in the regulation of cell proliferation. Belongs to the GAREM family.
kmeans	1	Red	#ff0000	94	GOT2	9606.ENSP00000245206	Aspartate aminotransferase, mitochondrial; Catalyzes the irreversible transamination of the L-tryptophan metabolite L-kynurenine to form kynurenic acid (KA). Plays a key role in amino acid metabolism. Important for metabolite exchange between mitochondria and cytosol. Facilitates cellular uptake of long-chain free fatty acids; Belongs to the class-I

							pyridoxal-phosphate-dependent aminotransferase family.
kmeans	1	Red	#ff0000	94	GRB2	9606.ENSP00000376345	Growth factor receptor-bound protein 2; Adapter protein that provides a critical link between cell surface growth factor receptors and the Ras signaling pathway; Belongs to the GRB2/sem-5/DRK family.
kmeans	1	Red	#ff0000	94	GRWD1	9606.ENSP00000253237	Glutamate-rich WD repeat-containing protein 1; Histone binding-protein that regulates chromatin dynamics and minichromosome maintenance (MCM) loading at replication origins, possibly by promoting chromatin openness.
kmeans	1	Red	#ff0000	94	GTF2F2	9606.ENSP00000340823	General transcription factor IIF subunit 2; TFIIF is a general transcription initiation factor that binds to RNA polymerase II and helps to recruit it to the initiation complex in collaboration with TFIIB. It promotes transcription elongation. This subunit shows ATP-dependent DNA-helicase activity.
kmeans	1	Red	#ff0000	94	HGS	9606.ENSP00000331201	Hepatocyte growth factor-regulated tyrosine kinase substrate; Involved in intracellular signal transduction mediated by cytokines and growth factors. When associated with STAM, it suppresses DNA signaling upon stimulation by IL-2 and GM-CSF. Could be a direct effector of PI3-kinase in vesicular pathway via early endosomes and may regulate trafficking to early and late endosomes by recruiting clathrin. May concentrate ubiquitinated receptors within clathrin-coated regions. Involved in down-regulation of receptor tyrosine kinase via multivesicular body (MVBs) when complexed with STAM (E [...]
kmeans	1	Red	#ff0000	94	HSPBP1	9606.ENSP00000255631	Hsp70-binding protein 1; Inhibits HSPA1A chaperone activity by changing the conformation of the ATP-binding domain of HSPA1A and interfering with ATP binding. Interferes with ubiquitination mediated by STUB1 and inhibits chaperone-assisted degradation of immature CFTR.
kmeans	1	Red	#ff0000	94	INTS2	9606.ENSP00000414237	Integrator complex subunit 2; Component of the Integrator (INT) complex, a complex involved in the small nuclear RNAs (snRNA) U1 and U2 transcription and in their 3'-box-dependent processing. The Integrator complex is associated with the C-terminal domain (CTD) of RNA polymerase II largest subunit (POLR2A) and is recruited to the U1 and U2 snRNAs genes (Probable). Mediates recruitment of cytoplasmic dynein to the nuclear envelope, probably as component of the INT complex.

kmeans	1	Red	#ff0000	94	INTS5	9606.ENSP00000327889	Integrator complex subunit 5; Component of the Integrator (INT) complex, a complex involved in the small nuclear RNAs (snRNA) U1 and U2 transcription and in their 3'-box-dependent processing. The Integrator complex is associated with the C-terminal domain (CTD) of RNA polymerase II largest subunit (POLR2A) and is recruited to the U1 and U2 snRNAs genes (Probable). Mediates recruitment of cytoplasmic dynein to the nuclear envelope, probably as component of the INT complex.
kmeans	1	Red	#ff0000	94	ITPR1	9606.ENSP00000306253	Inositol 1,4,5-trisphosphate receptor type 1; Intracellular channel that mediates calcium release from the endoplasmic reticulum following stimulation by inositol 1,4,5-trisphosphate. Involved in the regulation of epithelial secretion of electrolytes and fluid through the interaction with AHCYL1 (By similarity). Plays a role in ER stress-induced apoptosis. Cytoplasmic calcium released from the ER triggers apoptosis by the activation of CaM kinase II, eventually leading to the activation of downstream apoptosis pathways (By similarity).
kmeans	1	Red	#ff0000	94	LDHA	9606.ENSP00000445175	Lactate dehydrogenase A; Belongs to the LDH/MDH superfamily. LDH family.
kmeans	1	Red	#ff0000	94	MAPK14	9606.ENSP00000229795	Mitogen-activated protein kinase 14; Serine/threonine kinase which acts as an essential component of the MAP kinase signal transduction pathway. MAPK14 is one of the four p38 MAPKs which play an important role in the cascades of cellular responses evoked by extracellular stimuli such as proinflammatory cytokines or physical stress leading to direct activation of transcription factors. Accordingly, p38 MAPKs phosphorylate a broad range of proteins and it has been estimated that they may have approximately 200 to 300 substrates each. Some of the targets are downstream kinases which are a [...]
kmeans	1	Red	#ff0000	94	MED17	9606.ENSP00000251871	Mediator of RNA polymerase II transcription subunit 17; Component of the Mediator complex, a coactivator involved in the regulated transcription of nearly all RNA polymerase II-dependent genes. Mediator functions as a bridge to convey information from gene- specific regulatory proteins to the basal RNA polymerase II transcription machinery. Mediator is recruited to promoters by direct interactions with regulatory proteins and serves as a scaffold for the assembly of a functional preinitiation complex with RNA polymerase II and the general transcription factors.

kmeans	1	Red	#ff0000	94	MED20	9606.ENSP00000265350	Mediator of RNA polymerase II transcription subunit 20; Component of the Mediator complex, a coactivator involved in the regulated transcription of nearly all RNA polymerase II-dependent genes. Mediator functions as a bridge to convey information from gene- specific regulatory proteins to the basal RNA polymerase II transcription machinery. Mediator is recruited to promoters by direct interactions with regulatory proteins and serves as a scaffold for the assembly of a functional preinitiation complex with RNA polymerase II and the general transcription factors.
kmeans	1	Red	#ff0000	94	MED6	9606.ENSP00000481920	Mediator of RNA polymerase II transcription subunit 6; Component of the Mediator complex, a coactivator involved in the regulated transcription of nearly all RNA polymerase II-dependent genes. Mediator functions as a bridge to convey information from gene- specific regulatory proteins to the basal RNA polymerase II transcription machinery. Mediator is recruited to promoters by direct interactions with regulatory proteins and serves as a scaffold for the assembly of a functional preinitiation complex with RNA polymerase II and the general transcription factors.
kmeans	1	Red	#ff0000	94	MIF	9606.ENSP00000215754	Macrophage migration inhibitory factor; Pro-inflammatory cytokine. Involved in the innate immune response to bacterial pathogens. The expression of MIF at sites of inflammation suggests a role as mediator in regulating the function of macrophages in host defense. Counteracts the anti-inflammatory activity of glucocorticoids. Has phenylpyruvate tautomerase and dopachrome tautomerase activity (in vitro), but the physiological substrate is not known. It is not clear whether the tautomerase activity has any physiological relevance, and whether it is important for cytokine activity.
kmeans	1	Red	#ff0000	94	MOB4	9606.ENSP00000315702	MOB-like protein phocein; May play a role in membrane trafficking, specifically in membrane budding reactions; Belongs to the MOB1/phocein family.
kmeans	1	Red	#ff0000	94	MPST	9606.ENSP00000411719	3-mercaptopyruvate sulfurtransferase; Transfer of a sulfur ion to cyanide or to other thiol compounds. Also has weak rhodanese activity. Detoxifies cyanide and is required for thiosulfate biosynthesis. Acts as an antioxidant. In combination with cysteine aminotransferase (CAT), contributes to the catabolism of cysteine and is an important producer of hydrogen sulfide in the brain, retina and vascular endothelial cells. Hydrogen sulfide H(2)S is an important synaptic modulator, signaling molecule, smooth

							muscle contractor and neuroprotectant. Its production by the 3MST/CAT pathway is re [...]
kmeans	1	Red	#ff0000	94	MRPL18	9606.ENSP00000356001	39S ribosomal protein L18, mitochondrial; Together with thiosulfate sulfurtransferase (TST), acts as a mitochondrial import factor for the cytosolic 5S rRNA. The precursor form shows RNA chaperone activity; is able to fold the 5S rRNA into an import-competent conformation that is recognized by rhodanese (TST). Both the cytoplasmic and mitochondrial forms are able to bind to the helix IV-loop D in the gamma domain of the 5S rRNA.
kmeans	1	Red	#ff0000	94	MRPL23	9606.ENSP00000380466	Mitochondrial ribosomal protein L23.
kmeans	1	Red	#ff0000	94	MRPL24	9606.ENSP00000354525	Mitochondrial ribosomal protein L24; Belongs to the universal ribosomal protein uL24 family.
kmeans	1	Red	#ff0000	94	MRPS6	9606.ENSP00000382250	Mitochondrial ribosomal protein S6.
kmeans	1	Red	#ff0000	94	MRTO4	9606.ENSP00000364320	mRNA turnover protein 4 homolog; Component of the ribosome assembly machinery. Nuclear paralog of the ribosomal protein P0, it binds pre-60S subunits at an early stage of assembly in the nucleolus, and is replaced by P0 in cytoplasmic pre-60S subunits and mature 80S ribosomes.
kmeans	1	Red	#ff0000	94	MTR	9606.ENSP00000355536	Methionine synthase; Catalyzes the transfer of a methyl group from methyl-cobalamin to homocysteine, yielding enzyme-bound cob(I)alamin and methionine. Subsequently, remethylates the cofactor using methyltetrahydrofolate (By similarity); Belongs to the vitamin-B12 dependent methionine synthase family.
kmeans	1	Red	#ff0000	94	NELFA	9606.ENSP00000445757	Negative elongation factor A; Essential component of the NELF complex, a complex that negatively regulates the elongation of transcription by RNA polymerase II. The NELF complex, which acts via an association with the DSIF complex and causes transcriptional pausing, is counteracted by the P- TEFb kinase complex; Belongs to the NELF-A family.
kmeans	1	Red	#ff0000	94	NELFCD	9606.ENSP00000473290	Negative elongation factor C/D; Essential component of the NELF complex, a complex that negatively regulates the elongation of transcription by RNA polymerase II. The NELF complex, which acts via an association with the DSIF complex and causes transcriptional pausing, is counteracted by the P-TEFb kinase complex. Belongs to the NELF-D family.

kmeans	1	Red	#ff0000	94	NIP7	9606.ENSP00000254940	60S ribosome subunit biogenesis protein NIP7 homolog; Required for proper 34S pre-rRNA processing and 60S ribosome subunit assembly; Belongs to the NIP7 family.
kmeans	1	Red	#ff0000	94	NMD3	9606.ENSP00000417559	60S ribosomal export protein NMD3; Acts as an adapter for the XPO1/CRM1-mediated export of the 60S ribosomal subunit.
kmeans	1	Red	#ff0000	94	NOC4L	9606.ENSP00000328854	Nucleolar complex associated 4 homolog; Belongs to the CBF/MAK21 family.
kmeans	1	Red	#ff0000	94	NOL7	9606.ENSP00000405674	Nucleolar protein 7.
kmeans	1	Red	#ff0000	94	NOP9	9606.ENSP00000267425	NOP9 nucleolar protein.
kmeans	1	Red	#ff0000	94	PDPK1	9606.ENSP00000344220	3-phosphoinositide-dependent protein kinase 1; Serine/threonine kinase which acts as a master kinase, phosphorylating and activating a subgroup of the AGC family of protein kinases. Its targets include: protein kinase B (PKB/AKT1, PKB/AKT2, PKB/AKT3), p70 ribosomal protein S6 kinase (RPS6KB1), p90 ribosomal protein S6 kinase (RPS6KA1, RPS6KA2 and RPS6KA3), cyclic AMP-dependent protein kinase (PRKACA), protein kinase C (PRKCD and PRKCZ), serum and glucocorticoid-inducible kinase (SGK1, SGK2 and SGK3), p21-activated kinase-1 (PAK1), protein kinase PKN (PKN1 and PKN2). Plays a central rol [...]
kmeans	1	Red	#ff0000	94	PKN1	9606.ENSP00000343325	Serine/threonine-protein kinase N1; PKC-related serine/threonine-protein kinase involved in various processes such as regulation of the intermediate filaments of the actin cytoskeleton, cell migration, tumor cell invasion and transcription regulation. Part of a signaling cascade that begins with the activation of the adrenergic receptor ADRA1B and leads to the activation of MAPK14. Regulates the cytoskeletal network by phosphorylating proteins such as VIM and neurofilament proteins NEFH, NEFL and NEFM, leading to inhibit their polymerization. Phosphorylates 'Ser-575', 'Ser-637' and 'Se [...]
kmeans	1	Red	#ff0000	94	PNO1	9606.ENSP00000263657	RNA-binding protein PNO1; Positively regulates dimethylation of two adjacent adenosines in the loop of a conserved hairpin near the 3'-end of 18S rRNA.
kmeans	1	Red	#ff0000	94	POLR2E	9606.ENSP00000478303	DNA-directed RNA polymerases I, II, and III subunit RPABC1; DNA-dependent RNA polymerase catalyzes the transcription of DNA into RNA using the four ribonucleoside triphosphates as substrates. Common component of RNA polymerases I, II and III which synthesize ribosomal RNA precursors, mRNA precursors and many functional non- coding RNAs, and small RNAs, such as 5S rRNA and tRNAs, respectively. Pol II is the central component of the basal RNA polymerase II transcription machinery. Pols are composed of mobile elements that move relative to

							each other. In Pol II, POLR2E/RPB5 is part of the [...]
kmeans	1	Red	#ff0000	94	POLR2G	9606.ENSP00000301788	DNA-directed RNA polymerase II subunit RPB7; DNA-dependent RNA polymerase catalyzes the transcription of DNA into RNA using the four ribonucleoside triphosphates as substrates. Component of RNA polymerase II which synthesizes mRNA precursors and many functional non-coding RNAs. Pol II is the central component of the basal RNA polymerase II transcription machinery. It is composed of mobile elements that move relative to each other. RPB7 is part of a subcomplex with RPB4 that binds to a pocket formed by RPB1, RPB2 and RPB6 at the base of the clamp element. The RBP4-RPB7 subcomplex seems [...]
kmeans	1	Red	#ff0000	94	POLR2J	9606.ENSP00000292614	DNA-directed RNA polymerase II subunit RPB11-a; DNA-dependent RNA polymerase catalyzes the transcription of DNA into RNA using the four ribonucleoside triphosphates as substrates. Component of RNA polymerase II which synthesizes mRNA precursors and many functional non-coding RNAs. Pol II is the central component of the basal RNA polymerase II transcription machinery. It is composed of mobile elements that move relative to each other. RPB11 is part of the core element with the central large cleft (By similarity).
kmeans	1	Red	#ff0000	94	PPIF	9606.ENSP00000225174	Peptidyl-prolyl cis-trans isomerase F, mitochondrial; PPIase that catalyzes the cis-trans isomerization of proline imidic peptide bonds in oligopeptides and may therefore assist protein folding. Involved in regulation of the mitochondrial permeability transition pore (mPTP). It is proposed that its association with the mPTP is masking a binding site for inhibiting inorganic phosphate (Pi) and promotes the open probability of the mPTP leading to apoptosis or necrosis; the requirement of the PPIase activity for this function is debated. In cooperation with mitochondrial TP53 is involved [...]

kmeans	1	Red	#ff0000	94	PPP2CA	9606.ENSP00000418447	Serine/threonine-protein phosphatase 2A catalytic subunit alpha isoform; PP2A is the major phosphatase for microtubule-associated proteins (MAPs). PP2A can modulate the activity of phosphorylase B kinase casein kinase 2, mitogen-stimulated S6 kinase, and MAP-2 kinase. Cooperates with SGO2 to protect centromeric cohesin from separase- mediated cleavage in oocytes specifically during meiosis I (By similarity). Can dephosphorylate SV40 large T antigen and p53/TP53. Activates RAF1 by dephosphorylating it at 'Ser-259'.
kmeans	1	Red	#ff0000	94	PPP4C	9606.ENSP00000279387	Serine/threonine-protein phosphatase 4 catalytic subunit; Protein phosphatase that is involved in many processes such as microtubule organization at centrosomes, maturation of spliceosomal snRNPs, apoptosis, DNA repair, tumor necrosis factor (TNF)-alpha signaling, activation of c-Jun N-terminal kinase MAPK8, regulation of histone acetylation, DNA damage checkpoint signaling, NF-kappa-B activation and cell migration. The PPP4C-PPP4R1 PP4 complex may play a role in dephosphorylation and regulation of HDAC3. The PPP4C-PPP4R2- PPP4R3A PP4 complex specifically dephosphorylates H2AX phosphor [...]
kmeans	1	Red	#ff0000	94	PPP4R2	9606.ENSP00000349124	Serine/threonine-protein phosphatase 4 regulatory subunit 2; Regulatory subunit of serine/threonine-protein phosphatase 4 (PP4). May regulate the activity of PPP4C at centrosomal microtubule organizing centers. Its interaction with the SMN complex leads to enhance the temporal localization of snRNPs, suggesting a role of PPP4C in maturation of spliceosomal snRNPs. The PPP4C-PPP4R2-PPP4R3A PP4 complex specifically dephosphorylates H2AX phosphorylated on 'Ser-140' (gamma-H2AX) generated during DNA replication and required for DNA double strand break repair. Mediates RPA2 dephosphorylatio [...]
kmeans	1	Red	#ff0000	94	PRKACB	9606.ENSP00000359719	cAMP-dependent protein kinase catalytic subunit beta; Mediates cAMP-dependent signaling triggered by receptor binding to GPCRs. PKA activation regulates diverse cellular processes such as cell proliferation, the cell cycle, differentiation and regulation of microtubule dynamics, chromatin condensation and decondensation, nuclear envelope disassembly and reassembly, as well as regulation of intracellular transport mechanisms and ion flux. Regulates the abundance of compartmentalized pools of its regulatory subunits through phosphorylation of PJA2 which binds and ubiquitinates these subu [...]

kmeans	1	Red	#ff0000	94	PTPN11	9606.ENSP00000489597	Tyrosine-protein phosphatase non-receptor type 11; Acts downstream of various receptor and cytoplasmic protein tyrosine kinases to participate in the signal transduction from the cell surface to the nucleus. Positively regulates MAPK signal transduction pathway. Dephosphorylates GAB1, ARHGAP35 and EGFR. Dephosphorylates ROCK2 at 'Tyr-722' resulting in stimulatation of its RhoA binding activity. Dephosphorylates CDC73.
kmeans	1	Red	#ff0000	94	PTPN23	9606.ENSP00000265562	Tyrosine-protein phosphatase non-receptor type 23; Plays a role in sorting of endocytic ubiquitinated cargos into multivesicular bodies (MVBs) via its interaction with the ESCRT-I complex (endosomal sorting complex required for transport I), and possibly also other ESCRT complexes. May act as a negative regulator of Ras-mediated mitogenic activity. Plays a role in ciliogenesis.
kmeans	1	Red	#ff0000	94	QKI	9606.ENSP00000355094	Protein quaking; RNA-binding protein that plays a central role in myelinization. Binds to the 5'-NACUAAY-N(1,20)-UAAY- 3' RNA core sequence. Regulates target mRNA stability. In addition, acts by regulating pre-mRNA splicing, mRNA export and protein translation. Required to protect and promote stability of mRNAs such as MBP and CDKN1B. Regulator of oligodendrocyte differentiation and maturation in the brain that may play a role in myelin and oligodendrocyte dysfunction in schizophrenia. Participates in mRNA transport by regulating the nuclear export of MBP mRNA. Also involved in regulat [...]
kmeans	1	Red	#ff0000	94	RAB10	9606.ENSP00000264710	Ras-related protein Rab-10; The small GTPases Rab are key regulators of intracellular membrane trafficking, from the formation of transport vesicles to their fusion with membranes. Rabs cycle between an inactive GDP-bound form and an active GTP-bound form that is able to recruit to membranes different set of downstream effectors directly responsible for vesicle formation, movement, tethering and fusion. That Rab is mainly involved in the biosynthetic transport of proteins from the Golgi to the plasma membrane. Regulates, for instance, SLC2A4/GLUT4 glucose transporter-enriched vesicles [...]
kmeans	1	Red	#ff0000	94	RABIF	9606.ENSP00000356231	Guanine nucleotide exchange factor MSS4; Guanine-nucleotide-releasing protein that acts on members of the SEC4/YPT1/RAB subfamily. Stimulates GDP release from both YPT1 and RAB3A, but is less active on these proteins than on the SEC4 protein. Might play a general role in vesicular transport; Belongs to the DSS4/MSS4 family.

kmeans	1	Red	#ff0000	94	RAC1	9606.ENSP00000348461	Ras-related C3 botulinum toxin substrate 1; Plasma membrane-associated small GTPase which cycles between active GTP-bound and inactive GDP-bound states. In its active state, binds to a variety of effector proteins to regulate cellular responses such as secretory processes, phagocytosis of apoptotic cells, epithelial cell polarization, neurons adhesion, migration and differentiation, and growth-factor induced formation of membrane ruffles. Rac1 p21/rho GDI heterodimer is the active component of the cytosolic factor sigma 1, which is involved in stimulation of the NADPH oxidase activity [...]
kmeans	1	Red	#ff0000	94	RAC3	9606.ENSP00000304283	Ras-related C3 botulinum toxin substrate 3; Plasma membrane-associated small GTPase which cycles between an active GTP-bound and inactive GDP-bound state. In active state binds to a variety of effector proteins to regulate cellular responses, such as cell spreading and the formation of actin-based protusions including lamellipodia and membrane ruffles. Promotes cell adhesion and spreading on fibrinogen in a CIB1 and alpha-IIb/beta3 integrin-mediated manner.
kmeans	1	Red	#ff0000	94	RALA	9606.ENSP00000005257	Ras-related protein Ral-A; Multifunctional GTPase involved in a variety of cellular processes including gene expression, cell migration, cell proliferation, oncogenic transformation and membrane trafficking. Accomplishes its multiple functions by interacting with distinct downstream effectors. Acts as a GTP sensor for GTP-dependent exocytosis of dense core vesicles. The RALA-exocyst complex regulates integrin-dependent membrane raft exocytosis and growth signaling. Key regulator of LPAR1 signaling and competes with GRK2 for binding to LPAR1 thus affecting the signaling properties of t [...]
kmeans	1	Red	#ff0000	94	RAP1B	9606.ENSP00000250559	Ras-related protein Rap-1b; GTP-binding protein that possesses intrinsic GTPase activity. Contributes to the polarizing activity of KRIT1 and CDH5 in the establishment and maintenance of correct endothelial cell polarity and vascular lumen. Required for the localization of phosphorylated PRKCZ, PARD3 and TIAM1 to the cell junction. Plays a role in the establishment of basal endothelial barrier function; Belongs to the small GTPase superfamily. Ras family.

kmeans	1	Red	#ff0000	94	RHOA	9606.ENSP00000400175	Transforming protein RhoA; Small GTPase which cycles between an active GTP-bound and an inactive GDP-bound state. Mainly associated with cytoskeleton organization, in active state binds to a variety of effector proteins to regulate cellular responses such cytoskeletal dynamics, cell migration and cell cycle. Regulates a signal transduction pathway linking plasma membrane receptors to the assembly of focal adhesions and actin stress fibers. Involved in a microtubule-dependent signal that is required for the myosin contractile ring formation during cell cycle cytokinesis. Plays an essent [...]
kmeans	1	Red	#ff0000	94	RPL15	9606.ENSP00000309334	Ribosomal protein L15.
kmeans	1	Red	#ff0000	94	RPL24	9606.ENSP00000377640	Ribosomal protein L24; Belongs to the eukaryotic ribosomal protein eL24 family.
kmeans	1	Red	#ff0000	94	RPL32	9606.ENSP00000416429	Ribosomal protein L32; Belongs to the eukaryotic ribosomal protein eL32 family.
kmeans	1	Red	#ff0000	94	RPL36	9606.ENSP00000464342	60S ribosomal protein L36; Component of the large ribosomal subunit.
kmeans	1	Red	#ff0000	94	RPS10	9606.ENSP00000481646	40S ribosomal protein S10; Component of the 40S ribosomal subunit; Belongs to the eukaryotic ribosomal protein eS10 family.
kmeans	1	Red	#ff0000	94	RPS15	9606.ENSP00000466010	Ribosomal protein S15.
kmeans	1	Red	#ff0000	94	RPS18	9606.ENSP00000393241	40S ribosomal protein S18; Located at the top of the head of the 40S subunit, it contacts several helices of the 18S rRNA.
kmeans	1	Red	#ff0000	94	RPS23	9606.ENSP00000296674	40S ribosomal protein S23; Component of the ribosome, a large ribonucleoprotein complex responsible for the synthesis of proteins in the cell. The small ribosomal subunit (SSU) binds messenger RNAs (mRNAs) and translates the encoded message by selecting cognate aminoacyl-transfer RNA (tRNA) molecules. The large subunit (LSU) contains the ribosomal catalytic site termed the peptidyl transferase center (PTC), which catalyzes the formation of peptide bonds, thereby polymerizing the amino acids delivered by tRNAs into a polypeptide chain. The nascent polypeptides leave the ribosome through [...]
kmeans	1	Red	#ff0000	94	SLC12A2	9606.ENSP00000262461	Solute carrier family 12 member 2; Electrically silent transporter system. Mediates sodium and chloride reabsorption. Plays a vital role in the regulation of ionic balance and cell volume; Belongs to the SLC12A transporter family.
kmeans	1	Red	#ff0000	94	SLC25A6	9606.ENSP00000370808	ADP/ATP translocase 3, N-terminally processed; Catalyzes the exchange of cytoplasmic ADP with mitochondrial ATP across the mitochondrial inner membrane. May participate in the formation of the permeability transition pore complex (PTPC)

							responsible for the release of mitochondrial products that triggers apoptosis.
kmeans	1	Red	#ff0000	94	SMAD2	9606.ENSP00000262160	Mothers against decapentaplegic homolog 2; Receptor-regulated SMAD (R-SMAD) that is an intracellular signal transducer and transcriptional modulator activated by TGF-beta (transforming growth factor) and activin type 1 receptor kinases. Binds the TRE element in the promoter region of many genes that are regulated by TGF-beta and, on formation of the SMAD2/SMAD4 complex, activates transcription. May act as a tumor suppressor in colorectal carcinoma. Positively regulates PDPK1 kinase activity by stimulating its dissociation from the 14-3-3 protein YWHAQ which acts as a negative regulator.
kmeans	1	Red	#ff0000	94	SRP14	9606.ENSP00000267884	Signal recognition particle 14 kDa protein; Signal-recognition-particle assembly has a crucial role in targeting secretory proteins to the rough endoplasmic reticulum membrane. SRP9 together with SRP14 and the Alu portion of the SRP RNA, constitutes the elongation arrest domain of SRP. The complex of SRP9 and SRP14 is required for SRP RNA binding.
kmeans	1	Red	#ff0000	94	SSRP1	9606.ENSP00000278412	FACT complex subunit SSRP1; Component of the FACT complex, a general chromatin factor that acts to reorganize nucleosomes. The FACT complex is involved in multiple processes that require DNA as a template such as mRNA elongation, DNA replication and DNA repair. During transcription elongation the FACT complex acts as a histone chaperone that both destabilizes and restores nucleosomal structure. It facilitates the passage of RNA polymerase II and transcription by promoting the dissociation of one histone H2A-H2B dimer from the nucleosome, then subsequently promotes the reestablishment o [...]
kmeans	1	Red	#ff0000	94	STAMBP	9606.ENSP00000377633	STAM-binding protein; Zinc metalloprotease that specifically cleaves 'Lys-63'- linked polyubiquitin chains. Does not cleave 'Lys-48'-linked polyubiquitin chains (By similarity). Plays a role in signal transduction for cell growth and MYC induction mediated by IL-2 and GM- CSF. Potentiates BMP (bone morphogenetic protein) signaling by antagonizing the inhibitory action of SMAD6 and SMAD7. Has a key role in regulation of cell surface receptor-mediated endocytosis and ubiquitin-dependent sorting of receptors to lysosomes. Endosomal

							localization of STAMBP is required for efficient EGFR deg [...]
kmeans	1	Red	#ff0000	94	SUPT4H1	9606.ENSP00000225504	Transcription elongation factor SPT4; Component of the DRB sensitivity-inducing factor complex (DSIF complex), which regulates mRNA processing and transcription elongation by RNA polymerase II. DSIF positively regulates mRNA capping by stimulating the mRNA guanylyltransferase activity of RNGTT/CAP1A. DSIF also acts cooperatively with the negative elongation factor complex (NELF complex) to enhance transcriptional pausing at sites proximal to the promoter. Transcriptional pausing may facilitate the assembly of an elongation competent RNA polymerase II complex. DSIF and NELF promote paus [...]
kmeans	1	Red	#ff0000	94	TAB1	9606.ENSP00000216160	TGF-beta-activated kinase 1 and MAP3K7-binding protein 1; May be an important signaling intermediate between TGFB receptors and MAP3K7/TAK1. May play an important role in mammalian embryogenesis.
kmeans	1	Red	#ff0000	94	TBL1XR1	9606.ENSP00000405574	F-box-like/WD repeat-containing protein TBL1XR1; F-box-like protein involved in the recruitment of the ubiquitin/19S proteasome complex to nuclear receptor-regulated transcription units. Plays an essential role in transcription activation mediated by nuclear receptors. Probably acts as integral component of the N-Cor corepressor complex that mediates the recruitment of the 19S proteasome complex, leading to the subsequent proteasomal degradation of N-Cor complex, thereby allowing cofactor exchange, and transcription activation; Belongs to the WD repeat EBI family.
kmeans	1	Red	#ff0000	94	TPI1	9606.ENSP00000229270	Triosephosphate isomerase; Triosephosphate isomerase is an extremely efficient metabolic enzyme that catalyzes the interconversion between dihydroxyacetone phosphate (DHAP) and D-glyceraldehyde-3-phosphate (G3P) in glycolysis and gluconeogenesis.
kmeans	1	Red	#ff0000	94	TSFM	9606.ENSP00000313877	Elongation factor Ts, mitochondrial; Associates with the EF-Tu.GDP complex and induces the exchange of GDP to GTP. It remains bound to the aminoacyl-tRNA.EF- Tu.GTP complex up to the GTP hydrolysis stage on the ribosome. Belongs to the EF-Ts family.

kmeans	1	Red	#ff0000	94	TSG101	9606.ENSP00000251968	Tumor susceptibility gene 101 protein; Component of the ESCRT-I complex, a regulator of vesicular trafficking process. Binds to ubiquitinated cargo proteins and is required for the sorting of endocytic ubiquitinated cargos into multivesicular bodies (MVBs). Mediates the association between the ESCRT-0 and ESCRT-I complex. Required for completion of cytokinesis; the function requires CEP55. May be involved in cell growth and differentiation. Acts as a negative growth regulator. Involved in the budding of many viruses through an interaction with viral proteins that contain a late-budding [...]
kmeans	1	Red	#ff0000	94	UTP18	9606.ENSP00000225298	U3 small nucleolar RNA-associated protein 18 homolog; Involved in nucleolar processing of pre-18S ribosomal RNA. Belongs to the WD repeat UTP18 family.
kmeans	1	Red	#ff0000	94	UTP4	9606.ENSP00000327179	U3 small nucleolar RNA-associated protein 4 homolog; Ribosome biogenesis factor. Involved in nucleolar processing of pre-18S ribosomal RNA. Involved in small subunit (SSU) pre-rRNA processing at sites A', A0, 1 and 2b. Required for optimal pre- ribosomal RNA transcription by RNA polymerase. May be a transcriptional regulator. Acts as a positive regulator of HIVEP1 which specifically binds to the DNA sequence 5'-GGGACTTTCC-3' found in enhancer elements of numerous viral promoters such as those of HIV-1, SV40, or CMV.
kmeans	1	Red	#ff0000	94	VPS37B	9606.ENSP00000267202	Vacuolar protein sorting-associated protein 37B; Component of the ESCRT-I complex, a regulator of vesicular trafficking process. Required for the sorting of endocytic ubiquitinated cargos into multivesicular bodies. May be involved in cell growth and differentiation.
kmeans	1	Red	#ff0000	94	WDR43	9606.ENSP00000384302	WD repeat-containing protein 43; Ribosome biogenesis factor that coordinates hyperactive transcription and ribogenesis. Involved in nucleolar processing of pre-18S ribosomal RNA. Required for optimal pre-ribosomal RNA transcription by RNA polymerase I. Essential for stem cell pluripotency and embryonic development. In the nucleoplasm, recruited by promoter-associated/nascent transcripts and transcription to active promoters where it facilitates releases of elongation factor P-TEFb and paused RNA polymerase II to allow transcription elongation and maintain high-level expression of its t [...]

kmeans	2	Salmon	#ff8a65	39	ADAR	9606.ENSP00000357459	Double-stranded RNA-specific adenosine deaminase; Catalyzes the hydrolytic deamination of adenosine to inosine in double-stranded RNA (dsRNA) referred to as A-to-I RNA editing. This may affect gene expression and function in a number of ways that include mRNA translation by changing codons and hence the amino acid sequence of proteins; pre-mRNA splicing by altering splice site recognition sequences; RNA stability by changing sequences involved in nuclease recognition; genetic stability in the case of RNA virus genomes by changing sequences during viral RNA replication; and RNA structur [...]
kmeans	2	Salmon	#ff8a65	39	ago-02	9606.ENSP00000220592	Protein argonaute-2; Required for RNA-mediated gene silencing (RNAi) by the RNA- induced silencing complex (RISC). The 'minimal RISC' appears to include AGO2 bound to a short guide RNA such as a microRNA (miRNA) or short interfering RNA (siRNA). These guide RNAs direct RISC to complementary mRNAs that are targets for RISC-mediated gene silencing. The precise mechanism of gene silencing depends on the degree of complementarity between the miRNA or siRNA and its target. Binding of RISC to a perfectly complementary mRNA generally results in silencing due to endonucleolytic cleavage of the [...]
kmeans	2	Salmon	#ff8a65	39	EIF4A3	9606.ENSP00000497641	Eukaryotic initiation factor 4A-III, N-terminally processed; ATP-dependent RNA helicase. Involved in pre-mRNA splicing as component of the spliceosome. Core component of the splicing-dependent multiprotein exon junction complex (EJC) deposited at splice junctions on mRNAs. The EJC is a dynamic structure consisting of core proteins and several peripheral nuclear and cytoplasmic associated factors that join the complex only transiently either during EJC assembly or during subsequent mRNA metabolism. The EJC marks the position of the exon-exon junction in the mature mRNA for the gene expr [...]
kmeans	2	Salmon	#ff8a65	39	EIF4E	9606.ENSP00000425561	Eukaryotic translation initiation factor 4E; Recognizes and binds the 7-methylguanosine-containing mRNA cap during an early step in the initiation of protein synthesis and facilitates ribosome binding by inducing the unwinding of the mRNAs secondary structures. Component of the CYFIP1-EIF4E-FMR1 complex which binds to the mRNA cap and mediates translational repression. In the CYFIP1-EIF4E-FMR1 complex this subunit mediates the binding to the mRNA cap.

kmeans	2	Salmon	#ff8a65	39	EIF4E2	9606.ENSP00000258416	Eukaryotic translation initiation factor 4E type 2; Recognizes and binds the 7-methylguanosine-containing mRNA cap during an early step in the initiation. Acts as a repressor of translation initiation. In contrast to EIF4E, it is unable to bind eIF4G (EIF4G1, EIF4G2 or EIF4G3), suggesting that it acts by competing with EIF4E and block assembly of eIF4F at the cap (By similarity).
kmeans	2	Salmon	#ff8a65	39	ERH	9606.ENSP00000451080	Enhancer of rudimentary homolog; May have a role in the cell cycle; Belongs to the E(R) family.
kmeans	2	Salmon	#ff8a65	39	G3BP1	9606.ENSP00000377681	Ras GTPase-activating protein-binding protein 1; ATP- and magnesium-dependent helicase that plays an essential role in innate immunity. Participates in the DNA- triggered cGAS/STING pathway by promoting the DNA binding and activation of CGAS. Enhances also DDX58-induced type I interferon production probably by helping DDX58 at sensing pathogenic RNA. In addition, plays an essential role in stress granule formation. Unwinds preferentially partial DNA and RNA duplexes having a 17 bp annealed portion and either a hanging 3' tail or hanging tails at both 5'- and 3'-ends. Unwinds DNA/DNA, R [...]
kmeans	2	Salmon	#ff8a65	39	GSPT2	9606.ENSP00000341247	Eukaryotic peptide chain release factor GTP-binding subunit ERF3B; Involved in translation termination in response to the termination codons UAA, UAG and UGA. May play a role as a potent stimulator of the release factor activity of ETF1. Exhibits GTPase activity, which is ribosome- and ETF1-dependent. May play a role in cell cycle progression. Component of the transient SURF complex which recruits UPF1 to stalled ribosomes in the context of nonsense-mediated decay (NMD) of mRNAs containing premature stop codons.
kmeans	2	Salmon	#ff8a65	39	HNRNPA2B1	9606.ENSP00000346694	Heterogeneous nuclear ribonucleoproteins A2/B1; Heterogeneous nuclear ribonucleoprotein (hnRNP) that associates with nascent pre-mRNAs, packaging them into hnRNP particles. The hnRNP particle arrangement on nascent hnRNA is non-random and sequence-dependent and serves to condense and stabilize the transcripts and minimize tangling and knotting. Packaging plays a role in various processes such as transcription, pre-mRNA processing, RNA nuclear export, subcellular location, mRNA translation and stability of mature mRNAs. Forms hnRNP particles with at least 20 other different hnRNP and he [...]

kmeans	2	Salmon	#ff8a65	39	JMJD6	9606.ENSP00000394085	Bifunctional arginine demethylase and lysyl-hydroxylase JMJD6; Dioxygenase that can both act as a arginine demethylase and a lysyl-hydroxylase. Acts as a lysyl-hydroxylase that catalyzes 5-hydroxylation on specific lysine residues of target proteins such as U2AF2/U2AF65 and LUC7L2. Regulates RNA splicing by mediating 5-hydroxylation of U2AF2/U2AF65, affecting the pre-mRNA splicing activity of U2AF2/U2AF65. Hydroxylates its own N-terminus, which is required for homooligomerization. In addition to peptidyl-lysine 5-dioxygenase activity, may act as an RNA hydroxylase, as suggested by its [...]
kmeans	2	Salmon	#ff8a65	39	6 LSM	9606.ENSP00000296581	U6 snRNA-associated Sm-like protein LSm6; Plays role in pre-mRNA splicing as component of the U4/U6-U5 tri-snRNP complex that is involved in spliceosome assembly, and as component of the precatalytic spliceosome (spliceosome B complex). The heptameric LSM2-8 complex binds specifically to the 3'-terminal U-tract of U6 snRNA. Component of LSm protein complexes, which are involved in RNA processing and may function in a chaperone-like manner, facilitating the efficient association of RNA processing factors with their substrates. Component of the cytoplasmic LSM1-LSM7 complex, which is tho [...]
kmeans	2	Salmon	#ff8a65	39	NCBP2	9606.ENSP00000326806	Nuclear cap-binding protein subunit 2; Component of the cap-binding complex (CBC), which binds co- transcriptionally to the 5' cap of pre-mRNAs and is involved in various processes such as pre-mRNA splicing, translation regulation, nonsense- mediated mRNA decay, RNA-mediated gene silencing (RNAi) by microRNAs (miRNAs) and mRNA export. The CBC complex is involved in mRNA export from the nucleus via its interaction with ALYREF/THOC4/ALY, leading to the recruitment of the mRNA export machinery to the 5' end of mRNA and to mRNA export in a 5' to 3' direction through the nuclear pore. The C [...]
kmeans	2	Salmon	#ff8a65	39	PABPC1	9606.ENSP00000313007	Polyadenylate-binding protein 1; Binds the poly(A) tail of mRNA, including that of its own transcript, and regulates processes of mRNA metabolism such as pre-mRNA splicing and mRNA stability. Its function in translational initiation regulation can either be enhanced by PAIP1 or repressed by PAIP2. Can probably bind to cytoplasmic RNA sequences other than poly(A) in vivo. Involved in translationally coupled mRNA turnover. Implicated with other RNA-binding proteins in the cytoplasmic deadenylation/translational and decay interplay of the FOS mRNA mediated by the major coding-region deter [...]

kmeans	2	Salmon	#ff8a65	39	PABPC4	9606.ENSP00000361949	Polyadenylate-binding protein 4; Binds the poly(A) tail of mRNA. May be involved in cytoplasmic regulatory processes of mRNA metabolism. Can probably bind to cytoplasmic RNA sequences other than poly(A) in vivo (By similarity).
kmeans	2	Salmon	#ff8a65	39	PAIP1	9606.ENSP00000302768	Polyadenylate-binding protein-interacting protein 1; Acts as a coactivator in the regulation of translation initiation of poly(A)-containing mRNAs. Its stimulatory activity on translation is mediated via its action on PABPC1. Competes with PAIP2 for binding to PABPC1. Its association with EIF4A and PABPC1 may potentiate contacts between mRNA termini. May also be involved in translationally coupled mRNA turnover. Implicated with other RNA- binding proteins in the cytoplasmic deadenylation/translational and decay interplay of the FOS mRNA mediated by the major coding-region determinant o [...]
kmeans	2	Salmon	#ff8a65	39	PAPSS1	9606.ENSP00000265174	Bifunctional 3'-phosphoadenosine 5'-phosphosulfate synthase 1; Bifunctional enzyme with both ATP sulfurylase and APS kinase activity, which mediates two steps in the sulfate activation pathway. The first step is the transfer of a sulfate group to ATP to yield adenosine 5'-phosphosulfate (APS), and the second step is the transfer of a phosphate group from ATP to APS yielding 3'-phosphoadenylylsulfate (PAPS: activated sulfate donor used by sulfotransferase). In mammals, PAPS is the sole source of sulfate; APS appears to be only an intermediate in the sulfate-activation pathway. Required [...]
kmeans	2	Salmon	#ff8a65	39	PCBP2	9606.ENSP00000352438	Poly(rC)-binding protein 2; Single-stranded nucleic acid binding protein that binds preferentially to oligo dC. Major cellular poly(rC)-binding protein. Binds also poly(rU). Negatively regulates cellular antiviral responses mediated by MAVS signaling. It acts as an adapter between MAVS and the E3 ubiquitin ligase ITCH, therefore triggering MAVS ubiquitination and degradation.
kmeans	2	Salmon	#ff8a65	39	PPIH	9606.ENSP00000306614	Peptidyl-prolyl cis-trans isomerase H; PPIase that catalyzes the cis-trans isomerization of proline imidic peptide bonds in oligopeptides and may therefore assist protein folding. Participates in pre-mRNA splicing. May play a role in the assembly of the U4/U5/U6 tri-snRNP complex, one of the building blocks of the spliceosome. May act as a chaperone.
kmeans	2	Salmon	#ff8a65	39	RBM42	9606.ENSP00000262633	RNA-binding protein 42; Binds (via the RRM domain) to the 3'-untranslated region (UTR) of CDKN1A mRNA.

kmeans	2	Salmon	#ff8a65	39	SAP18	9606.ENSP00000481842	Histone deacetylase complex subunit SAP18; Component of the SIN3-repressing complex. Enhances the ability of SIN3-HDAC1-mediated transcriptional repression. When tethered to the promoter, it can direct the formation of a repressive complex to core histone proteins. Auxiliary component of the splicing- dependent multiprotein exon junction complex (EJC) deposited at splice junction on mRNAs. The EJC is a dynamic structure consisting of core proteins and several peripheral nuclear and cytoplasmic associated factors that join the complex only transiently either during EJC assembly or durin [...]
kmeans	2	Salmon	#ff8a65	39	SF3B5	9606.ENSP00000356541	Splicing factor 3B subunit 5; Involved in pre-mRNA splicing as a component of the splicing factor SF3B complex, a constituent of the spliceosome. SF3B complex is required for 'A' complex assembly formed by the stable binding of U2 snRNP to the branchpoint sequence (BPS) in pre-mRNA. Sequence independent binding of SF3A/SF3B complex upstream of the branch site is essential, it may anchor U2 snRNP to the pre-mRNA.
kmeans	2	Salmon	#ff8a65	39	SNRNP27	9606.ENSP00000244227	U4/U6.U5 small nuclear ribonucleoprotein 27 kDa protein; May play a role in mRNA splicing.
kmeans	2	Salmon	#ff8a65	39	SNRPA	9606.ENSP00000243563	U1 small nuclear ribonucleoprotein A; Component of the spliceosomal U1 snRNP, which is essential for recognition of the pre-mRNA 5' splice-site and the subsequent assembly of the spliceosome. U1 snRNP is the first snRNP to interact with pre-mRNA. This interaction is required for the subsequent binding of U2 snRNP and the U4/U6/U5 tri-snRNP. SNRPA binds stem loop II of U1 snRNA. In a snRNP-free form (SF-A) may be involved in coupled pre-mRNA splicing and polyadenylation process. May bind preferentially to the 5'-UGCAC-3' motif on RNAs; Belongs to the RRM U1 A/B'' family.
kmeans	2	Salmon	#ff8a65	39	SNRPB	9606.ENSP00000412566	Small nuclear ribonucleoprotein-associated proteins B and B; Plays role in pre-mRNA splicing as core component of the SMN- Sm complex that mediates spliceosomal snRNP assembly and as component of the spliceosomal U1, U2, U4 and U5 small nuclear ribonucleoproteins (snRNPs), the building blocks of the spliceosome. Component of both the pre-catalytic spliceosome B complex and activated spliceosome C complexes. Is also a component of the minor U12 spliceosome. As part of the U7 snRNP it is involved in histone pre-mRNA 3'-end processing.

kmeans	2	Salmon	#ff8a65	39	SNRPB2	9606.ENSP00000246071	U2 small nuclear ribonucleoprotein B'; Involved in pre-mRNA splicing as component of the spliceosome. Associated with sn-RNP U2, where it contributes to the binding of stem loop IV of U2 snRNA.
kmeans	2	Salmon	#ff8a65	39	SNRPD1	9606.ENSP00000300413	Small nuclear ribonucleoprotein Sm D1; Plays role in pre-mRNA splicing as core component of the SMN- Sm complex that mediates spliceosomal snRNP assembly and as component of the spliceosomal U1, U2, U4 and U5 small nuclear ribonucleoproteins (snRNPs), the building blocks of the spliceosome. Component of both the pre-catalytic spliceosome B complex and activated spliceosome C complexes. Is also a component of the minor U12 spliceosome. May act as a charged protein scaffold to promote snRNP assembly or strengthen snRNP-snRNP interactions through non-specific electrostatic contacts with R [...]
kmeans	2	Salmon	#ff8a65	39	SNRPD3	9606.ENSP00000215829	Small nuclear ribonucleoprotein Sm D3; Plays role in pre-mRNA splicing as core component of the SMN- Sm complex that mediates spliceosomal snRNP assembly and as component of the spliceosomal U1, U2, U4 and U5 small nuclear ribonucleoproteins (snRNPs), the building blocks of the spliceosome. Component of both the pre-catalytic spliceosome B complex and activated spliceosome C complexes. Is also a component of the minor U12 spliceosome. As part of the U7 snRNP it is involved in histone pre-mRNA 3'-end processing (By similarity).
kmeans	2	Salmon	#ff8a65	39	SNRPE	9606.ENSP00000400591	Small nuclear ribonucleoprotein E; Plays role in pre-mRNA splicing as core component of the SMN- Sm complex that mediates spliceosomal snRNP assembly and as component of the spliceosomal U1, U2, U4 and U5 small nuclear ribonucleoproteins (snRNPs), the building blocks of the spliceosome. Component of both the pre-catalytic spliceosome B complex and activated spliceosome C complexes. Is also a component of the minor U12 spliceosome. As part of the U7 snRNP it is involved in histone 3'-end processing. May indirectly play a role in hair development.
kmeans	2	Salmon	#ff8a65	39	SNRPG	9606.ENSP00000393388	Small nuclear ribonucleoprotein G; Plays role in pre-mRNA splicing as core component of the SMN- Sm complex that mediates spliceosomal snRNP assembly and as component of the spliceosomal U1, U2, U4 and U5 small nuclear ribonucleoproteins (snRNPs), the building blocks of the spliceosome. Component of both the pre-catalytic spliceosome B complex and activated spliceosome C complexes. Is also a component of the minor U12 spliceosome. As part of the U7 snRNP it is involved in histone 3'-end processing.

kmeans	2	Salmon	#ff8a65	39	SRSF10	9606.ENSP00000420195	Serine/arginine-rich splicing factor 10; Splicing factor that in its dephosphorylated form acts as a general repressor of pre-mRNA splicing. Seems to interfere with the U1 snRNP 5'-splice recognition of SNRNP70. Required for splicing repression in M-phase cells and after heat shock. Also acts as a splicing factor that specifically promotes exon skipping during alternative splicing. Interaction with YTHDC1, a RNA-binding protein that recognizes and binds N6-methyladenosine (m6A)-containing RNAs, prevents SRSF10 from binding to its mRNA-binding sites close to m6A-containing regions, lead [...]
kmeans	2	Salmon	#ff8a65	39	SRSF6	9606.ENSP00000244020	Serine/arginine-rich splicing factor 6; Plays a role in constitutive splicing and modulates the selection of alternative splice sites. Plays a role in the alternative splicing of MAPT/Tau exon 10. Binds to alternative exons of TNC pre- mRNA and promotes the expression of alternatively spliced TNC. Plays a role in wound healing and in the regulation of keratinocyte differentiation and proliferation via its role in alternative splicing. Belongs to the splicing factor SR family.
kmeans	2	Salmon	#ff8a65	39	TAF6L	9606.ENSP00000294168	TAF6-like RNA polymerase II p300/CBP-associated factor-associated factor 65 kDa subunit 6L; Functions as a component of the PCAF complex. The PCAF complex is capable of efficiently acetylating histones in a nucleosomal context. The PCAF complex could be considered as the human version of the yeast SAGA complex (Probable). With TAF5L, acts as an epigenetic regulator essential for somatic reprogramming. Regulates target genes through H3K9ac deposition and MYC recruitment which trigger MYC regulatory network to orchestrate gene expression programs to control embryonic stem cell state. Fun [...]
kmeans	2	Salmon	#ff8a65	39	TARDBP	9606.ENSP00000240185	TAR DNA-binding protein 43; RNA-binding protein that is involved in various steps of RNA biogenesis and processing. Preferentially binds, via its two RNA recognition motifs RRM1 and RRM2, to GU-repeats on RNA molecules predominantly localized within long introns and in the 3'UTR of mRNAs. In turn, regulates the splicing of many non-coding and protein-coding RNAs including proteins involved in neuronal survival, as well as mRNAs that encode proteins relevant for neurodegenerative diseases. Plays a role in maintaining mitochondrial homeostasis by regulating the processing of mitochondria [...]

kmeans	2	Salmon	#ff8a65	39	TIA1	9606.ENSP00000401371	Nucleolysin TIA-1 isoform p40; Involved in alternative pre-RNA splicing and regulation of mRNA translation by binding to AU-rich elements (AREs) located in mRNA 3' untranslated regions (3' UTRs). Possesses nucleolytic activity against cytotoxic lymphocyte target cells. May be involved in apoptosis.
kmeans	2	Salmon	#ff8a65	39	TRA2A	9606.ENSP00000297071	Transformer-2 protein homolog alpha; Sequence-specific RNA-binding protein which participates in the control of pre-mRNA splicing.
kmeans	2	Salmon	#ff8a65	39	U2AF1	9606.ENSP00000291552	Splicing factor U2AF 35 kDa subunit; Plays a critical role in both constitutive and enhancer- dependent splicing by mediating protein-protein interactions and protein-RNA interactions required for accurate 3'-splice site selection. Recruits U2 snRNP to the branch point. Directly mediates interactions between U2AF2 and proteins bound to the enhancers and thus may function as a bridge between U2AF2 and the enhancer complex to recruit it to the adjacent intron.
kmeans	2	Salmon	#ff8a65	39	U2AF2	9606.ENSP00000307863	Splicing factor U2AF 65 kDa subunit; Plays a role in pre-mRNA splicing and 3'-end processing. By recruiting PRPF19 and the PRP19C/Prp19 complex/NTC/Nineteen complex to the RNA polymerase II C-terminal domain (CTD), and thereby pre-mRNA, may couple transcription to splicing. Induces cardiac troponin-T (TNNT2) pre-mRNA exon inclusion in muscle. Regulates the TNNT2 exon 5 inclusion through competition with MBNL1. Binds preferentially to a single-stranded structure within the polypyrimidine tract of TNNT2 intron 4 during spliceosome assembly. Required for the export of mRNA out of the nucl [...]
kmeans	2	Salmon	#ff8a65	39	UPF1	9606.ENSP00000470142	Regulator of nonsense transcripts 1; RNA-dependent helicase and ATPase required for nonsense- mediated decay (NMD) of mRNAs containing premature stop codons. Is recruited to mRNAs upon translation termination and undergoes a cycle of phosphorylation and dephosphorylation; its phosphorylation appears to be a key step in NMD. Recruited by release factors to stalled ribosomes together with the SMG1C protein kinase complex to form the transient SURF (SMG1-UPF1-eRF1-eRF3) complex. In EJC-dependent NMD, the SURF complex associates with the exon junction complex (EJC) (located 50-55 or more n [...]

kmeans	2	Salmon	#ff8a65	39	YJU2	9606.ENSP00000262962	Splicing factor YJU2; Part of the spliceosome which catalyzes two sequential transesterification reactions, first the excision of the non-coding intron from pre-mRNA and then the ligation of the coding exons to form the mature mRNA. Plays a role in stabilizing the structure of the spliceosome catalytic core and docking of the branch helix into the active site, producing 5'-exon and lariat intron-3'-intermediates (By similarity). May protect cells from TP53-dependent apoptosis upon dsDNA break damage through association with PRP19-CD5L complex ; Belongs to the CWC16 family. YJU2 subfamily.
kmeans	3	Dark Golden Rod	#b25500	17	ADRM1	9606.ENSP00000478877	Proteasomal ubiquitin receptor ADRM1; Component of the 26S proteasome, a multiprotein complex involved in the ATP-dependent degradation of ubiquitinated proteins. This complex plays a key role in the maintenance of protein homeostasis by removing misfolded or damaged proteins, which could impair cellular functions, and by removing proteins whose functions are no longer required. Therefore, the proteasome participates in numerous cellular processes, including cell cycle progression, apoptosis, or DNA damage repair. Within the complex, functions as a proteasomal ubiquitin receptor. Engag [...]
kmeans	3	Dark Golden Rod	#b25500	17	ATM	9606.ENSP00000278616	Serine-protein kinase ATM; Serine/threonine protein kinase which activates checkpoint signaling upon double strand breaks (DSBs), apoptosis and genotoxic stresses such as ionizing ultraviolet A light (UVA), thereby acting as a DNA damage sensor. Recognizes the substrate consensus sequence [ST]-Q. Phosphorylates 'Ser-139' of histone variant H2AX at double strand breaks (DSBs), thereby regulating DNA damage response mechanism. Also plays a role in pre-B cell allelic exclusion, a process leading to expression of a single immunoglobulin heavy chain allele to enforce clonality and monospec [...]
kmeans	3	Dark Golden Rod	#b25500	17	CHD1L	9606.ENSP00000358262	Chromodomain-helicase-DNA-binding protein 1-like; DNA helicase which plays a role in chromatin-remodeling following DNA damage. Targeted to sites of DNA damage through interaction with poly(ADP-ribose) and functions to regulate chromatin during DNA repair. Able to catalyze nucleosome sliding in an ATP-dependent manner. Helicase activity is strongly stimulated upon poly(ADP-ribose)-binding; Belongs to the SNF2/RAD54 helicase family.

kmeans	3	Dark Golden Rod	#b25500	17	PSMA2	9606.ENSP00000223321	Proteasome subunit alpha type-2; Component of the 20S core proteasome complex involved in the proteolytic degradation of most intracellular proteins. This complex plays numerous essential roles within the cell by associating with different regulatory particles. Associated with two 19S regulatory particles, forms the 26S proteasome and thus participates in the ATP- dependent degradation of ubiquitinated proteins. The 26S proteasome plays a key role in the maintenance of protein homeostasis by removing misfolded or damaged proteins that could impair cellular functions, and by removing pr [...]
kmeans	3	Dark Golden Rod	#b25500	17	PSMA3	9606.ENSP00000216455	Proteasome subunit alpha type-3; Component of the 20S core proteasome complex involved in the proteolytic degradation of most intracellular proteins. This complex plays numerous essential roles within the cell by associating with different regulatory particles. Associated with two 19S regulatory particles, forms the 26S proteasome and thus participates in the ATP- dependent degradation of ubiquitinated proteins. The 26S proteasome plays a key role in the maintenance of protein homeostasis by removing misfolded or damaged proteins that could impair cellular functions, and by removing pr [...]
kmeans	3	Dark Golden Rod	#b25500	17	PSMA4	9606.ENSP00000044462	Proteasome subunit alpha type-4; Component of the 20S core proteasome complex involved in the proteolytic degradation of most intracellular proteins. This complex plays numerous essential roles within the cell by associating with different regulatory particles. Associated with two 19S regulatory particles, forms the 26S proteasome and thus participates in the ATP- dependent degradation of ubiquitinated proteins. The 26S proteasome plays a key role in the maintenance of protein homeostasis by removing misfolded or damaged proteins that could impair cellular functions, and by removing pr [...]
kmeans	3	Dark Golden Rod	#b25500	17	PSMA6	9606.ENSP00000261479	Proteasome subunit alpha type-6; Component of the 20S core proteasome complex involved in the proteolytic degradation of most intracellular proteins. This complex plays numerous essential roles within the cell by associating with different regulatory particles. Associated with two 19S regulatory particles, forms the 26S proteasome and thus participates in the ATP- dependent degradation of ubiquitinated proteins. The 26S proteasome plays a key role in the maintenance of protein homeostasis by removing misfolded or damaged proteins that could impair cellular functions, and by removing pr [...]

kmeans	3	Dark Golden Rod	#b25500	17	PSMB1	9606.ENSP00000262193	Proteasome subunit beta type-1; Component of the 20S core proteasome complex involved in the proteolytic degradation of most intracellular proteins. This complex plays numerous essential roles within the cell by associating with different regulatory particles. Associated with two 19S regulatory particles, forms the 26S proteasome and thus participates in the ATP- dependent degradation of ubiquitinated proteins. The 26S proteasome plays a key role in the maintenance of protein homeostasis by removing misfolded or damaged proteins that could impair cellular functions, and by removing pro [...]
kmeans	3	Dark Golden Rod	#b25500	17	PSMB2	9606.ENSP00000362334	Proteasome subunit beta type-2; Component of the 20S core proteasome complex involved in the proteolytic degradation of most intracellular proteins. This complex plays numerous essential roles within the cell by associating with different regulatory particles. Associated with two 19S regulatory particles, forms the 26S proteasome and thus participates in the ATP- dependent degradation of ubiquitinated proteins. The 26S proteasome plays a key role in the maintenance of protein homeostasis by removing misfolded or damaged proteins that could impair cellular functions, and by removing pro [...]
kmeans	3	Dark Golden Rod	#b25500	17	PSMB3	9606.ENSP00000483688	Proteasome subunit beta type-3; Component of the 20S core proteasome complex involved in the proteolytic degradation of most intracellular proteins. This complex plays numerous essential roles within the cell by associating with different regulatory particles. Associated with two 19S regulatory particles, forms the 26S proteasome and thus participates in the ATP- dependent degradation of ubiquitinated proteins. The 26S proteasome plays a key role in the maintenance of protein homeostasis by removing misfolded or damaged proteins that could impair cellular functions, and by removing pro [...]
kmeans	3	Dark Golden Rod	#b25500	17	PSMB4	9606.ENSP00000290541	Proteasome subunit beta type-4; Component of the 20S core proteasome complex involved in the proteolytic degradation of most intracellular proteins. This complex plays numerous essential roles within the cell by associating with different regulatory particles. Associated with two 19S regulatory particles, forms the 26S proteasome and thus participates in the ATP- dependent degradation of ubiquitinated proteins. The 26S proteasome plays a key role in the maintenance of protein homeostasis by removing misfolded or damaged proteins that could impair cellular functions, and by removing pro [...]

kmeans	3	Dark Golden Rod	#b25500	17	PSMB5	9606.ENSP00000355325	Proteasome subunit beta type-5; Component of the 20S core proteasome complex involved in the proteolytic degradation of most intracellular proteins. This complex plays numerous essential roles within the cell by associating with different regulatory particles. Associated with two 19S regulatory particles, forms the 26S proteasome and thus participates in the ATP- dependent degradation of ubiquitinated proteins. The 26S proteasome plays a key role in the maintenance of protein homeostasis by removing misfolded or damaged proteins that could impair cellular functions, and by removing pro [...]
kmeans	3	Dark Golden Rod	#b25500	17	RAD23A	9606.ENSP00000467024	UV excision repair protein RAD23 homolog A; Multiubiquitin chain receptor involved in modulation of proteasomal degradation. Binds to 'Lys-48'-linked polyubiquitin chains in a length-dependent manner and with a lower affinity to 'Lys-63'- linked polyubiquitin chains. Proposed to be capable to bind simultaneously to the 26S proteasome and to polyubiquitinated substrates and to deliver ubiquitinated proteins to the proteasome. (Microbial infection) Involved in Vpr-dependent replication of HIV-1 in non-proliferating cells and primary macrophages. Required for the association of HIV-1 Vpr [...]
kmeans	3	Dark Golden Rod	#b25500	17	RAD23B	9606.ENSP00000350708	UV excision repair protein RAD23 homolog B; Multiubiquitin chain receptor involved in modulation of proteasomal degradation. Binds to polyubiquitin chains. Proposed to be capable to bind simultaneously to the 26S proteasome and to polyubiquitinated substrates and to deliver ubiquitinated proteins to the proteasome. May play a role in endoplasmic reticulum-associated degradation (ERAD) of misfolded glycoproteins by association with PNGase and delivering deglycosylated proteins to the proteasome. The XPC complex is proposed to represent the first factor bound at the sites of DNA damage a [...]
kmeans	3	Dark Golden Rod	#b25500	17	TERF2	9606.ENSP00000254942	Telomeric repeat-binding factor 2; Binds the telomeric double-stranded 5'-TTAGGG-3' repeat and plays a central role in telomere maintenance and protection against end-to-end fusion of chromosomes. In addition to its telomeric DNA- binding role, required to recruit a number of factors and enzymes required for telomere protection, including the shelterin complex, TERF2IP/RAP1 and DCLRE1B/Apollo. Component of the shelterin complex (telosome) that is involved in the regulation of telomere length and protection. Shelterin associates with arrays of double-

							stranded 5'- TTAGGG-3' repeats added [...]
kmeans	3	Dark Golden Rod	#b25500	17	UBQLN4	9606.ENSP00000357292	Ubiquilin-4; Regulator of protein degradation that mediates the proteasomal targeting of misfolded, mislocalized or accumulated proteins. Acts by binding polyubiquitin chains of target proteins via its UBA domain and by interacting with subunits of the proteasome via its ubiquitin-like domain. Key regulator of DNA repair that represses homologous recombination repair: in response to DNA damage, recruited to sites of DNA damage following phosphorylation by ATM and acts by binding and removing ubiquitinated MRE11 from damaged chromatin, leading to MRE11 degradation by the proteasome. MRE [...]
kmeans	3	Dark Golden Rod	#b25500	17	XRCC6	9606.ENSP00000352257	X-ray repair cross-complementing protein 6; Single-stranded DNA-dependent ATP-dependent helicase. Has a role in chromosome translocation. The DNA helicase II complex binds preferentially to fork-like ends of double-stranded DNA in a cell cycle-dependent manner. It works in the 3'-5' direction. Binding to DNA may be mediated by XRCC6. Involved in DNA non-homologous end joining (NHEJ) required for double-strand break repair and V(D)J recombination. The XRCC5/6 dimer acts as regulatory subunit of the DNA-dependent protein kinase complex DNA-PK by increasing the affinity of the catalytic s [...]
kmeans	4	Sandy Brown	#ffd465	9	ADK	9606.ENSP00000443965	Adenosine kinase; ATP dependent phosphorylation of adenosine and other related nucleoside analogs to monophosphate derivatives. Serves as a potential regulator of concentrations of extracellular adenosine and intracellular adenine nucleotides; Belongs to the carbohydrate kinase PfkB family.
kmeans	4	Sandy Brown	#ffd465	9	ADSL	9606.ENSP00000485525	Adenylosuccinate lyase; Catalyzes two non-sequential steps in de novo AMP synthesis: converts (S)-2-(5-amino-1-(5-phospho-D-ribosyl)imidazole-4-carboxamido)succinate (SAICAR) to fumarate plus 5-amino-1-(5-phospho-D- ribosyl)imidazole-4-carboxamide, and thereby also contributes to de novo IMP synthesis, and converts succinyladenosine monophosphate (SAMP) to AMP and fumarate.

kmeans	4	Sandy Brown	#ffd465	9	AK1	9606.ENSP00000223836	Adenylate kinase isoenzyme 1; Catalyzes the reversible transfer of the terminal phosphate group between ATP and AMP. Also displays broad nucleoside diphosphate kinase activity. Plays an important role in cellular energy homeostasis and in adenine nucleotide metabolism.
kmeans	4	Sandy Brown	#ffd465	9	GUK1	9606.ENSP00000355689	Guanylate kinase; Catalyzes the phosphorylation of GMP to GDP. Essential enzyme for recycling GMP and indirectly, cyclic GMP (cGMP). Involved in the cGMP metabolism in photoreceptors (By similarity). It may also have a role in the survival and growth progression of some tumors. In addition to its physiological role, GUK1 is essential for convert prodrugs used for the treatment of cancers and viral infections into their pharmacologically active metabolites, most notably acyclovir, ganciclovir, and 6-thioguanine and its closely related analog 6-mercaptopurine. Belongs to the guanylate ki [...]
kmeans	4	Sandy Brown	#ffd465	9	HPRT1	9606.ENSP00000298556	Hypoxanthine-guanine phosphoribosyltransferase; Converts guanine to guanosine monophosphate, and hypoxanthine to inosine monophosphate. Transfers the 5-phosphoribosyl group from 5-phosphoribosylpyrophosphate onto the purine. Plays a central role in the generation of purine nucleotides through the purine salvage pathway.
kmeans	4	Sandy Brown	#ffd465	9	NAMPT	9606.ENSP00000222553	Nicotinamide phosphoribosyltransferase; Catalyzes the condensation of nicotinamide with 5- phosphoribosyl-1-pyrophosphate to yield nicotinamide mononucleotide, an intermediate in the biosynthesis of NAD. It is the rate limiting component in the mammalian NAD biosynthesis pathway. The secreted form behaves both as a cytokine with immunomodulating properties and an adipokine with anti-diabetic properties, it has no enzymatic activity, partly because of lack of activation by ATP, which has a low level in extracellular space and plasma. Plays a role in the modulation of circadian clock fun [...]
kmeans	4	Sandy Brown	#ffd465	9	NT5C1A	9606.ENSP00000235628	Cytosolic 5'-nucleotidase 1A; Dephosphorylates the 5' and 2'(3')-phosphates of deoxyribonucleotides and has a broad substrate specificity. Helps to regulate adenosine levels in heart during ischemia and hypoxia.
kmeans	4	Sandy Brown	#ffd465	9	PNP	9606.ENSP00000354532	Purine nucleoside phosphorylase; The purine nucleoside phosphorylases catalyze the phosphorolytic breakdown of the N-glycosidic bond in the beta-(deoxy)ribonucleoside molecules, with the formation of the corresponding free purine bases and pentose-1-phosphate.

kmeans	4	Sandy Brown	#ffd465	9	SIRT6	9606.ENSP00000337332	NAD-dependent protein deacetylase sirtuin-6; NAD-dependent protein deacetylase. Has deacetylase activity towards histone H3K9Ac and H3K56Ac. Modulates acetylation of histone H3 in telomeric chromatin during the S-phase of the cell cycle. Deacetylates histone H3K9Ac at NF-kappa-B target promoters and may down-regulate the expression of a subset of NF-kappa-B target genes. Acts as a corepressor of the transcription factor HIF1A to control the expression of multiple glycolytic genes to regulate glucose homeostasis. Required for genomic stability. Regulates the production of TNF protein. H [...]
kmeans	5	Brown	#b2ab00	7	TUBA1A	9606.ENSP00000439020	Detyrosinated tubulin alpha-1A chain; Tubulin is the major constituent of microtubules. It binds two moles of GTP, one at an exchangeable site on the beta chain and one at a non-exchangeable site on the alpha chain.
kmeans	5	Brown	#b2ab00	7	TUBAL3	9606.ENSP00000369784	Tubulin alpha chain-like 3; Tubulin is the major constituent of microtubules. It binds two moles of GTP, one at an exchangeable site on the beta chain and one at a non-exchangeable site on the alpha chain (By similarity). Belongs to the tubulin family.
kmeans	5	Brown	#b2ab00	7	TUBB	9606.ENSP00000339001	Tubulin beta chain; Tubulin is the major constituent of microtubules. It binds two moles of GTP, one at an exchangeable site on the beta chain and one at a non-exchangeable site on the alpha chain.
kmeans	5	Brown	#b2ab00	7	TUBB2A	9606.ENSP00000369703	Tubulin beta-2A chain; Tubulin is the major constituent of microtubules. It binds two moles of GTP, one at an exchangeable site on the beta chain and one at a non-exchangeable site on the alpha chain (By similarity).
kmeans	5	Brown	#b2ab00	7	TUBB3	9606.ENSP00000320295	Tubulin beta-3 chain; Tubulin is the major constituent of microtubules. It binds two moles of GTP, one at an exchangeable site on the beta chain and one at a non-exchangeable site on the alpha chain. TUBB3 plays a critical role in proper axon guidance and mantainance. Binding of NTN1/Netrin-1 to its receptor UNC5C might cause dissociation of UNC5C from polymerized TUBB3 in microtubules and thereby lead to increased microtubule dynamics and axon repulsion. Plays a role in dorsal root ganglion axon projection towards the spinal cord.
kmeans	5	Brown	#b2ab00	7	TUBB4A	9606.ENSP00000264071	Tubulin beta-4A chain; Tubulin is the major constituent of microtubules. It binds two moles of GTP, one at an exchangeable site on the beta chain and one at a non-exchangeable site on the alpha chain.

kmeans	5	Brown	#b2ab00	7	TUBB6	9606.ENSP00000318697	Tubulin beta-6 chain; Tubulin is the major constituent of microtubules. It binds two moles of GTP, one at an exchangeable site on the beta chain and one at a non-exchangeable site on the alpha chain. Belongs to the tubulin family.
kmeans	6	Yellow	#e0ff65	7	ABRAXAS2	9606.ENSP00000298492	BRISC complex subunit Abraxas 2; Component of the BRISC complex, a multiprotein complex that specifically cleaves 'Lys-63'-linked polyubiquitin, leaving the last ubiquitin chain attached to its substrates. May act as a central scaffold protein that assembles the various components of the BRISC complex and retains them in the cytoplasm. Plays a role in regulating the onset of apoptosis via its role in modulating 'Lys-63'-linked ubiquitination of target proteins (By similarity). Required for normal mitotic spindle assembly and microtubule attachment to kinetochores via its role in deubiq [...]
kmeans	6	Yellow	#e0ff65	7	BRCC3	9606.ENSP00000358474	Lys-63-specific deubiquitinase BRCC36; Metalloprotease that specifically cleaves 'Lys-63'-linked polyubiquitin chains. Does not have activity toward 'Lys- 48'-linked polyubiquitin chains. Component of the BRCA1-A complex, a complex that specifically recognizes 'Lys-63'-linked ubiquitinated histones H2A and H2AX at DNA lesions sites, leading to target the BRCA1-BARD1 heterodimer to sites of DNA damage at double-strand breaks (DSBs). In the BRCA1-A complex, it specifically removes 'Lys-63'-linked ubiquitin on histones H2A and H2AX, antagonizing the RNF8-dependent ubiquitination at double [...]
kmeans	6	Yellow	#e0ff65	7	CBX2	9606.ENSP00000308750	Chromobox protein homolog 2; Component of a Polycomb group (PcG) multiprotein PRC1-like complex, a complex class required to maintain the transcriptionally repressive state of many genes, including Hox genes, throughout development. PcG PRC1 complex acts via chromatin remodeling and modification of histones; it mediates monoubiquitination of histone H2A 'Lys-119', rendering chromatin heritably changed in its expressibility. Binds to histone H3 trimethylated at 'Lys-9' (H3K9me3) or at 'Lys-27' (H3K27me3) (By similarity). Plays a role in the lineage differentiation of the germ layers in [...]
kmeans	6	Yellow	#e0ff65	7	CBX8	9606.ENSP00000269385	Chromobox protein homolog 8; Component of a Polycomb group (PcG) multiprotein PRC1-like complex, a complex class required to maintain the transcriptionally repressive state of many genes, including Hox genes, throughout development. PcG PRC1 complex acts via chromatin remodeling and modification of histones; it mediates monoubiquitination of

							histone H2A 'Lys-119', rendering chromatin heritably changed in its expressibility.
kmeans	6	Yellow	#e0ff65	7	EED	9606.ENSP00000338186	Polycomb protein EED; Polycomb group (PcG) protein. Component of the PRC2/EED-EZH2 complex, which methylates 'Lys-9' and 'Lys-27' of histone H3, leading to transcriptional repression of the affected target gene. Also recognizes 'Lys-26' trimethylated histone H1 with the effect of inhibiting PRC2 complex methyltransferase activity on nucleosomal histone H3 'Lys-27', whereas H3 'Lys-27' recognition has the opposite effect, enabling the propagation of this repressive mark. The PRC2/EED- EZH2 complex may also serve as a recruiting platform for DNA methyltransferases, thereby linking two ep [...]
kmeans	6	Yellow	#e0ff65	7	SUZ12	9606.ENSP00000316578	Polycomb protein SUZ12; Polycomb group (PcG) protein. Component of the PRC2/EED-EZH2 complex, which methylates 'Lys-9' (H3K9me) and 'Lys-27' (H3K27me) of histone H3, leading to transcriptional repression of the affected target gene. The PRC2/EED-EZH2 complex may also serve as a recruiting platform for DNA methyltransferases, thereby linking two epigenetic repression systems. Genes repressed by the PRC2/EED-EZH2 complex include HOXC8, HOXA9, MYT1 and CDKN2A.
kmeans	6	Yellow	#e0ff65	7	YY1	9606.ENSP00000262238	Transcriptional repressor protein YY1; Multifunctional transcription factor that exhibits positive and negative control on a large number of cellular and viral genes by binding to sites overlapping the transcription start site. Binds to the consensus sequence 5'-CCGCCATNTT-3'; some genes have been shown to contain a longer binding motif allowing enhanced binding; the initial CG dinucleotide can be methylated greatly reducing the binding affinity. The effect on transcription regulation is depending upon the context in which it binds and diverse mechanisms of action include direct activa [...]
kmeans	7	Olive Drab	#63b200	5	ACAT2	9606.ENSP00000356015	Acetyl-CoA acetyltransferase, cytosolic; Involved in the biosynthetic pathway of cholesterol.
kmeans	7	Olive Drab	#63b200	5	HMGCS1	9606.ENSP00000322706	Hydroxymethylglutaryl-CoA synthase, cytoplasmic; This enzyme condenses acetyl-CoA with acetoacetyl-CoA to form HMG-CoA, which is the substrate for HMG-CoA reductase.
kmeans	7	Olive Drab	#63b200	5	HSD17B7	9606.ENSP00000254521	3-keto-steroid reductase; Responsible for the reduction of the keto group on the C-3 of sterols; Belongs to the short-

							chain dehydrogenases/reductases (SDR) family. ERG27 subfamily.
kmeans	7	Olive Drab	#63b200	5	IDI1	9606.ENSP00000370748	Isopentenyl-diphosphate Delta-isomerase 1; Catalyzes the 1,3-allylic rearrangement of the homoallylic substrate isopentenyl (IPP) to its highly electrophilic allylic isomer, dimethylallyl diphosphate (DMAPP).
kmeans	7	Olive Drab	#63b200	5	NSDHL	9606.ENSP00000359297	Sterol-4-alpha-carboxylate 3-dehydrogenase, decarboxylating; Involved in the sequential removal of two C-4 methyl groups in post-squalene cholesterol biosynthesis. Belongs to the 3-beta-HSD family.
kmeans	8	Green	#96ff65	4	C17orf49	9606.ENSP00000448598	Chromatin complexes subunit BAP18; Component of chromatin complexes such as the MLL1/MLL and NURF complexes.
kmeans	8	Green	#96ff65	4	DPY30	9606.ENSP00000345837	Protein dpy-30 homolog; As part of the MLL1/MLL complex, involved in the methylation of histone H3 at 'Lys-4', particularly trimethylation. Histone H3 'Lys- 4' methylation represents a specific tag for epigenetic transcriptional activation. May play some role in histone H3 acetylation. In a teratocarcinoma cell, plays a crucial role in retinoic acid-induced differentiation along the neural lineage, regulating gene induction and H3 'Lys-4' methylation at key developmental loci. May also play an indirect or direct role in endosomal transport. Belongs to the dpy-30 family.
kmeans	8	Green	#96ff65	4	TWF2	9606.ENSP00000303908	Twinfilin-2; Actin-binding protein involved in motile and morphological processes. Inhibits actin polymerization, likely by sequestering G- actin. By capping the barbed ends of filaments, it also regulates motility. Seems to play an important role in clathrin-mediated endocytosis and distribution of endocytic organelles. May play a role in regulating the mature length of the middle and short rows of stereocilia (By similarity); Belongs to the actin-binding proteins ADF family. Twinfilin subfamily.
kmeans	8	Green	#96ff65	4	WDR82	9606.ENSP00000296490	WD repeat-containing protein 82; Regulatory component of the SET1 complex implicated in the tethering of this complex to transcriptional start sites of active genes. Facilitates histone H3 'Lys-4' methylation via recruitment of the SETD1A or SETD1B to the 'Ser-5' phosphorylated C-terminal domain (CTD) of RNA polymerase II large subunit (POLR2A). Component of PTW/PP1 phosphatase complex, which plays a role in the control of chromatin structure and cell cycle progression during the transition from mitosis into interphase.

kmeans	9	Green 2	#0eb200	4	ATP5PB	9606.ENSP00000358737	ATP synthase F(0) complex subunit B1, mitochondrial; Mitochondrial membrane ATP synthase (F(1)F(0) ATP synthase or Complex V) produces ATP from ADP in the presence of a proton gradient across the membrane which is generated by electron transport complexes of the respiratory chain. F-type ATPases consist of two structural domains, F(1) - containing the extramembraneous catalytic core, and F(0) - containing the membrane proton channel, linked together by a central stalk and a peripheral stalk. During catalysis, ATP synthesis in the catalytic domain of F(1) is coupled via a rotary mechani [...]
kmeans	9	Green 2	#0eb200	4	COX5A	9606.ENSP00000317780	Cytochrome c oxidase subunit 5A, mitochondrial; Component of the cytochrome c oxidase, the last enzyme in the mitochondrial electron transport chain which drives oxidative phosphorylation. The respiratory chain contains 3 multisubunit complexes succinate dehydrogenase (complex II, CII), ubiquinol- cytochrome c oxidoreductase (cytochrome b-c1 complex, complex III, CIII) and cytochrome c oxidase (complex IV, CIV), that cooperate to transfer electrons derived from NADH and succinate to molecular oxygen, creating an electrochemical gradient over the inner membrane that drives transmembrane [...]
kmeans	9	Green 2	#0eb200	4	COX6B1	9606.ENSP00000466818	Cytochrome c oxidase subunit 6B1; Component of the cytochrome c oxidase, the last enzyme in the mitochondrial electron transport chain which drives oxidative phosphorylation. The respiratory chain contains 3 multisubunit complexes succinate dehydrogenase (complex II, CII), ubiquinol- cytochrome c oxidoreductase (cytochrome b-c1 complex, complex III, CIII) and cytochrome c oxidase (complex IV, CIV), that cooperate to transfer electrons derived from NADH and succinate to molecular oxygen, creating an electrochemical gradient over the inner membrane that drives transmembrane transport and [...]
kmeans	9	Green 2	#0eb200	4	MT-CO2	9606.ENSP00000354876	Cytochrome c oxidase subunit 2; Component of the cytochrome c oxidase, the last enzyme in the mitochondrial electron transport chain which drives oxidative phosphorylation. The respiratory chain contains 3 multisubunit complexes succinate dehydrogenase (complex II, CII), ubiquinol- cytochrome c oxidoreductase (cytochrome b-c1 complex, complex III, CIII) and cytochrome c oxidase (complex IV, CIV), that cooperate to transfer electrons derived from NADH and succinate to molecular oxygen, creating an

							electrochemical gradient over the inner membrane that drives transmembrane transport and t [...]
kmeans	10	Light Green	#65ff7e	3	NUP62	9606.ENSP00000471191	Nuclear pore glycoprotein p62; Essential component of the nuclear pore complex. The N-terminal is probably involved in nucleocytoplasmic transport. The C-terminal is involved in protein-protein interaction probably via coiled-coil formation, promotes its association with centrosomes and may function in anchorage of p62 to the pore complex. Plays a role in mitotic cell cycle progression by regulating centrosome segregation, centriole maturation and spindle orientation. It might be involved in protein recruitment to the centrosome after nuclear breakdown.
kmeans	10	Light Green	#65ff7e	3	NUP85	9606.ENSP00000245544	Nuclear pore complex protein Nup85; Essential component of the nuclear pore complex (NPC) that seems to be required for NPC assembly and maintenance. As part of the NPC Nup107-160 subcomplex plays a role in RNA export and in tethering NUP96/Nup98 and NUP153 to the nucleus. The Nup107-160 complex seems to be required for spindle assembly during mitosis. NUP85 is required for membrane clustering of CCL2-activated CCR2. Seems to be involved in CCR2-mediated chemotaxis of monocytes and may link activated CCR2 to the phosphatidyl-inositol 3-kinase-Rac-lammellipodium protrusion cascade. Inv [...]
kmeans	10	Light Green	#65ff7e	3	NUTF2	9606.ENSP00000219169	Nuclear transport factor 2; Mediates the import of GDP-bound RAN from the cytoplasm into the nucleus which is essential for the function of RAN in cargo receptor-mediated nucleocytoplasmic transport. Thereby, plays indirectly a more general role in cargo receptor-mediated nucleocytoplasmic transport. Interacts with GDP-bound RAN in the cytosol, recruits it to the nuclear pore complex via its interaction with nucleoporins and promotes its nuclear import.
kmeans	11	Cornflower Blue	#75a6ef	3	COMMD3	9606.ENSP00000366032	COMM domain-containing protein 3; May modulate activity of cullin-RING E3 ubiquitin ligase (CRL) complexes. May down-regulate activation of NF- kappa-B. Modulates Na(+) transport in epithelial cells by regulation of apical cell surface expression of amiloride-sensitive sodium channel (ENaC) subunits.

kmeans	11	Cornflower Blue	#75a6ef	3	COMMD5	9606.ENSP00000394331	COMM domain-containing protein 5; May modulate activity of cullin-RING E3 ubiquitin ligase (CRL) complexes. Negatively regulates cell proliferation. Negatively regulates cell cycle G2/M phase transition probably by transactivating p21/CDKN1A through the p53/TP53-independent signaling pathway. Involved in kidney proximal tubule morphogenesis (By similarity). Down-regulates activation of NF-kappa-B.
kmeans	11	Cornflower Blue	#75a6ef	3	COMMD9	9606.ENSP00000263401	COMM domain-containing protein 9; May modulate activity of cullin-RING E3 ubiquitin ligase (CRL) complexes. May down-regulate activation of NF- kappa-B. Modulates Na(+) transport in epithelial cells by regulation of apical cell surface expression of amiloride-sensitive sodium channel (ENaC) subunits.
kmeans	12	Blue	#7588ef	3	GNAI2	9606.ENSP00000312999	Guanine nucleotide-binding protein G(i) subunit alpha-2; Guanine nucleotide-binding proteins (G proteins) are involved as modulators or transducers in various transmembrane signaling systems. The G(i) proteins are involved in hormonal regulation of adenylate cyclase: they inhibit the cyclase in response to beta- adrenergic stimuli. May play a role in cell division.
kmeans	12	Blue	#7588ef	3	GNB2	9606.ENSP00000305260	Guanine nucleotide-binding protein G(I)/G(S)/G(T) subunit beta-2; Guanine nucleotide-binding proteins (G proteins) are involved as a modulator or transducer in various transmembrane signaling systems. The beta and gamma chains are required for the GTPase activity, for replacement of GDP by GTP, and for G protein-effector interaction.
kmeans	12	Blue	#7588ef	3	LYN	9606.ENSP00000428924	Tyrosine-protein kinase Lyn; Non-receptor tyrosine-protein kinase that transmits signals from cell surface receptors and plays an important role in the regulation of innate and adaptive immune responses, hematopoiesis, responses to growth factors and cytokines, integrin signaling, but also responses to DNA damage and genotoxic agents. Functions primarily as negative regulator, but can also function as activator, depending on the context. Required for the initiation of the B-cell response, but also for its down-regulation and termination. Plays an important role in the regulation of B-c [...]
kmeans	13	Purple	#7f75ef	3	ESD	9606.ENSP00000367992	S-formylglutathione hydrolase; Serine hydrolase involved in the detoxification of formaldehyde.
kmeans	13	Purple	#7f75ef	3	GLO1	9606.ENSP00000362463	Lactoylglutathione lyase; Catalyzes the conversion of hemimercaptal, formed from methylglyoxal and glutathione, to S-lactoylglutathione. Involved in the regulation of TNF-induced transcriptional activity of NF-kappa-B.

							Required for normal osteoclastogenesis.
kmeans	13	Purple	#7f75ef	3	HAGH	9606.ENSP00000380514	Hydroxyacylglutathione hydrolase, mitochondrial; Thiolesterase that catalyzes the hydrolysis of S-D-lactoyl-glutathione to form glutathione and D-lactic acid.
kmeans	14	Medium Purple	#9c75ef	2	GNPDA1	9606.ENSP00000423674	Glucosamine-6-phosphate isomerase 1; Seems to trigger calcium oscillations in mammalian eggs. These oscillations serve as the essential trigger for egg activation and early development of the embryo (By similarity); Belongs to the glucosamine/galactosamine-6-phosphate isomerase family.
kmeans	14	Medium Purple	#9c75ef	2	GNPNAT1	9606.ENSP00000216410	Glucosamine-phosphate N-acetyltransferase 1; Belongs to the acetyltransferase family. GNA1 subfamily.
kmeans	15	Medium Orchid	#b975ef	2	EIF2B2	9606.ENSP00000266126	Translation initiation factor eIF-2B subunit beta; Catalyzes the exchange of eukaryotic initiation factor 2- bound GDP for GTP; Belongs to the eIF-2B alpha/beta/delta subunits family.
kmeans	15	Medium Orchid	#b975ef	2	EIF2B4	9606.ENSP00000429323	Translation initiation factor eIF-2B subunit delta; Catalyzes the exchange of eukaryotic initiation factor 2- bound GDP for GTP.
kmeans	16	Orchid	#d775ef	2	LAMP1	9606.ENSP00000333298	Lysosome-associated membrane glycoprotein 1; Presents carbohydrate ligands to selectins. Also implicated in tumor cell metastasis.
kmeans	16	Orchid	#d775ef	2	MIOS	9606.ENSP00000339881	GATOR complex protein MIOS; As a component of the GATOR subcomplex GATOR2, functions within the amino acid-sensing branch of the TORC1 signaling pathway. Indirectly activates mTORC1 and the TORC1 signaling pathway through the inhibition of the GATOR1 subcomplex. It is negatively regulated by the upstream amino acid sensors SESN2 and CASTOR1.
kmeans	17	Violet	#ef75ea	2	ARIH2	9606.ENSP00000348769	E3 ubiquitin-protein ligase ARIH2; E3 ubiquitin-protein ligase, which catalyzes ubiquitination of target proteins together with ubiquitin-conjugating enzyme E2 UBE2L3. Acts as an atypical E3 ubiquitin-protein ligase by working together with cullin-5-RING ubiquitin ligase complex (ECS complex, also named CRL5 complex) and initiating ubiquitination of ECS substrates: associates with ECS complex and specifically mediates addition of the first ubiquitin on ECS targets (By similarity). The initial ubiquitin is then elongated (By similarity). E3 ubiquitin-protein ligase activity is activated [...]
kmeans	17	Violet	#ef75ea	2	UBE2L3	9606.ENSP00000400906	Ubiquitin conjugating enzyme E2 L3.

kmeans	18	Orchid 2	#ef75cd	2	LAMTOR2	9606.ENSP00000357288	Ragulator complex protein LAMTOR2; As part of the Ragulator complex it is involved in amino acid sensing and activation of mTORC1, a signaling complex promoting cell growth in response to growth factors, energy levels, and amino acids. Activated by amino acids through a mechanism involving the lysosomal V- ATPase, the Ragulator functions as a guanine nucleotide exchange factor activating the small GTPases Rag. Activated Ragulator and Rag GTPases function as a scaffold recruiting mTORC1 to lysosomes where it is in turn activated. Adapter protein that enhances the efficiency of the MAP k [...]
kmeans	18	Orchid 2	#ef75cd	2	LAMTOR3	9606.ENSP00000424183	Ragulator complex protein LAMTOR3; As part of the Ragulator complex it is involved in amino acid sensing and activation of mTORC1, a signaling complex promoting cell growth in response to growth factors, energy levels, and amino acids. Activated by amino acids through a mechanism involving the lysosomal V- ATPase, the Ragulator functions as a guanine nucleotide exchange factor activating the small GTPases Rag. Activated Ragulator and Rag GTPases function as a scaffold recruiting mTORC1 to lysosomes where it is in turn activated. Adapter protein that enhances the efficiency of the MAP k [...]
kmeans	19	Hot Pink	#ef75b0	2	PAFAH1B2	9606.ENSP00000435289	Platelet-activating factor acetylhydrolase IB subunit beta; Inactivates PAF by removing the acetyl group at the sn-2 position. This is a catalytic subunit.
kmeans	19	Hot Pink	#ef75b0	2	PAFAH1B3	9606.ENSP00000444935	Platelet-activating factor acetylhydrolase IB subunit gamma; Inactivates paf by removing the acetyl group at the sn-2 position. This is a catalytic subunit. Plays an important role during the development of brain; Belongs to the 'GDSL' lipolytic enzyme family. Platelet- activating factor acetylhydrolase IB beta/gamma subunits subfamily.
kmeans	20	Pink	#ef7592	2	PPT1	9606.ENSP00000493153	Palmitoyl-protein thioesterase 1; Removes thioester-linked fatty acyl groups such as palmitate from modified cysteine residues in proteins or peptides during lysosomal degradation. Prefers acyl chain lengths of 14 to 18 carbons ; Belongs to the palmitoyl-protein thioesterase family.
kmeans	20	Pink	#ef7592	2	TPP1	9606.ENSP00000299427	Tripeptidyl-peptidase 1; Lysosomal serine protease with tripeptidyl-peptidase I activity. May act as a non-specific lysosomal peptidase which generates tripeptides from the breakdown products produced by lysosomal proteinases. Requires substrates with an unsubstituted N-terminus.

Supplementary Table S3: The table presents clusters of upregulated proteins (1 mM citicoline vs. untreated cells, 6h), which were identified through PPI analysis in STRING (refer to Supplementary Table 1). Clustering was performed by applying a stringent connectivity strength threshold of 0.9 and utilizing the knn algorithm. For each cluster, the table indicates the clustering method, a unique cluster number (consistent with the numbering in the Gene Ontology figures across the manuscript), the assigned cluster color (corresponding to the colors used in the GO charts), the protein name, protein identifier, and protein description.

Clustering Method	Cluster Number	Cluster Color	Hex Color	Gene Count	Protein Name	Protein Identifier	Protein Description
kmeans	1	Red	#ff0000	8	ERAL1	9606.ENSP00000254928	GTPase Era, mitochondrial; Probable GTPase that plays a role in the mitochondrial ribosomal small subunit assembly. Specifically binds the 12S mitochondrial rRNA (12S mt-rRNA) to a 33 nucleotide section delineating the 3' terminal stem-loop region. May act as a chaperone that protects the 12S mt-rRNA on the 28S mitoribosomal subunit during ribosomal small subunit assembly; Belongs to the TRAFAC class TrmE-Era-EngA-EngB-Septin-like GTPase superfamily. Era GTPase family.
kmeans	1	Red	#ff0000	8	MRPL13	9606.ENSP00000306548	Mitochondrial ribosomal protein L13; Belongs to the universal ribosomal protein uL13 family.
kmeans	1	Red	#ff0000	8	MRPL49	9606.ENSP00000279242	Mitochondrial ribosomal protein L49.
kmeans	1	Red	#ff0000	8	MRPS15	9606.ENSP00000362208	Mitochondrial ribosomal protein S15; Belongs to the universal ribosomal protein uS15 family.
kmeans	1	Red	#ff0000	8	MRPS18B	9606.ENSP00000259873	Mitochondrial ribosomal protein S18B; Belongs to the bacterial ribosomal protein bS18 family. Mitochondrion-specific ribosomal protein mS40 subfamily.
kmeans	1	Red	#ff0000	8	MRPS31	9606.ENSP00000315397	Mitochondrial ribosomal protein S31.
kmeans	1	Red	#ff0000	8	MRPS35	9606.ENSP00000081029	Mitochondrial ribosomal protein S35.
kmeans	1	Red	#ff0000	8	MRPS36	9606.ENSP00000256441	Mitochondrial ribosomal protein S36.
kmeans	2	Salmon	#ff8e65	6	ACO2	9606.ENSP00000216254	Aconitate hydratase, mitochondrial; Catalyzes the isomerization of citrate to isocitrate via cis- aconitate; Belongs to the aconitase/IPM isomerase family.
kmeans	2	Salmon	#ff8e65	6	MMUT	9606.ENSP00000274813	Methylmalonyl-CoA mutase, mitochondrial; Involved in the degradation of several amino acids, odd-chain fatty acids and cholesterol via propionyl-CoA to the tricarboxylic acid cycle. MCM has different functions in other species; Belongs to the methylmalonyl-CoA mutase family.
kmeans	2	Salmon	#ff8e65	6	NDUFB9	9606.ENSP00000276689	NADH dehydrogenase [ubiquinone] 1 beta subcomplex subunit 9; Accessory subunit of the mitochondrial membrane respiratory chain NADH dehydrogenase (Complex I), that is believed to be not involved in catalysis. Complex I functions in the transfer of electrons from NADH to the respiratory chain. The immediate electron acceptor for the enzyme is believed to be ubiquinone.

kmeans	2	Salmon	#ff8e65	6	NDUFS3	9606.ENSP00000263774	NADH dehydrogenase [ubiquinone] iron-sulfur protein 3, mitochondrial; Core subunit of the mitochondrial membrane respiratory chain NADH dehydrogenase (Complex I) that is believed to belong to the minimal assembly required for catalysis. Complex I functions in the transfer of electrons from NADH to the respiratory chain. The immediate electron acceptor for the enzyme is believed to be ubiquinone (By similarity).
kmeans	2	Salmon	#ff8e65	6	SUCLG1	9606.ENSP00000377446	Succinate--CoA ligase [ADP/GDP-forming] subunit alpha, mitochondrial; Succinyl-CoA synthetase functions in the citric acid cycle (TCA), coupling the hydrolysis of succinyl-CoA to the synthesis of either ATP or GTP and thus represents the only step of substrate-level phosphorylation in the TCA. The alpha subunit of the enzyme binds the substrates coenzyme A and phosphate, while succinate binding and specificity for either ATP or GTP is provided by different beta subunits.
kmeans	2	Salmon	#ff8e65	6	UQCRQ	9606.ENSP00000367939	Cytochrome b-c1 complex subunit 8; Component of the ubiquinol-cytochrome c oxidoreductase, a multisubunit transmembrane complex that is part of the mitochondrial electron transport chain which drives oxidative phosphorylation. The respiratory chain contains 3 multisubunit complexes succinate dehydrogenase (complex II, CII), ubiquinol-cytochrome c oxidoreductase (cytochrome b-c1 complex, complex III, CIII) and cytochrome c oxidase (complex IV, CIV), that cooperate to transfer electrons derived from NADH and succinate to molecular oxygen, creating an electrochemical gradient over the inn [...]
kmeans	3	Brown	#b25f00	4	AARS2	9606.ENSP00000244571	Alanine--tRNA ligase, mitochondrial; Catalyzes the attachment of alanine to tRNA(Ala) in a two- step reaction: alanine is first activated by ATP to form Ala-AMP and then transferred to the acceptor end of tRNA(Ala). Also edits incorrectly charged tRNA(Ala) via its editing domain. Belongs to the class-II aminoacyl-tRNA synthetase family.
kmeans	3	Brown	#b25f00	4	EARS2	9606.ENSP00000456218	Probable glutamate--tRNA ligase, mitochondrial; Catalyzes the attachment of glutamate to tRNA(Glu) in a two- step reaction: glutamate is first activated by ATP to form Glu-AMP and then transferred to the acceptor end of tRNA(Glu).
kmeans	3	Brown	#b25f00	4	GATB	9606.ENSP00000263985	Glutamyl-tRNA(Gln) amidotransferase subunit B, mitochondrial; Allows the formation of correctly charged Gln-tRNA(Gln) through the transamidation of misacylated Glu-tRNA(Gln) in the mitochondria. The reaction takes place in the presence of glutamine and ATP through an activated gamma-phospho-Glu-tRNA(Gln). Belongs to the GatB/GatE family. GatB subfamily.
kmeans	3	Brown	#b25f00	4	LARS2	9606.ENSP00000498867	Probable leucine--tRNA ligase, mitochondrial; leucyl-tRNA synthetase 2, mitochondrial.

kmeans	4	Yellow	#ffe065	3	SV2A	9606.ENSP00000358142	Synaptic vesicle glycoprotein 2A; Plays a role in the control of regulated secretion in neural and endocrine cells, enhancing selectively low-frequency neurotransmission. Positively regulates vesicle fusion by maintaining the readily releasable pool of secretory vesicles (By similarity).
kmeans	4	Yellow	#ffe065	3	SYT1	9606.ENSP00000261205	Synaptotagmin-1; Calcium sensor that participates in triggering neurotransmitter release at the synapse (By similarity). May have a regulatory role in the membrane interactions during trafficking of synaptic vesicles at the active zone of the synapse (By similarity). It binds acidic phospholipids with a specificity that requires the presence of both an acidic head group and a diacyl backbone. A Ca(2+)- dependent interaction between synaptotagmin and putative receptors for activated protein kinase C has also been reported. It can bind to at least three additional proteins in a Ca(2+)-in [...]
kmeans	4	Yellow	#ffe065	3	VAMP2	9606.ENSP00000418572	Vesicle-associated membrane protein 2; Involved in the targeting and/or fusion of transport vesicles to their target membrane. Modulates the gating characteristics of the delayed rectifier voltage-dependent potassium channel KCNB1. Belongs to the synaptobrevin family.
kmeans	5	Dark Golden Rod	#a6b200	3	CLPP	9606.ENSP00000245816	ATP-dependent Clp protease proteolytic subunit, mitochondrial; Protease component of the Clp complex that cleaves peptides and various proteins in an ATP-dependent process. Has low peptidase activity in the absence of CLPX. The Clp complex can degrade CSN1S1, CSN2 and CSN3, as well as synthetic peptides (in vitro) and may be responsible for a fairly general and central housekeeping function rather than for the degradation of specific substrates. Cleaves PINK1 in the mitochondrion.
kmeans	5	Dark Golden Rod	#a6b200	3	GRPEL1	9606.ENSP00000264954	GrpE protein homolog 1, mitochondrial; Essential component of the PAM complex, a complex required for the translocation of transit peptide-containing proteins from the inner membrane into the mitochondrial matrix in an ATP-dependent manner (By similarity). Seems to control the nucleotide-dependent binding of mitochondrial HSP70 to substrate proteins. Belongs to the GrpE family.
kmeans	5	Dark Golden Rod	#a6b200	3	TIMM44	9606.ENSP00000270538	Mitochondrial import inner membrane translocase subunit TIM44; Essential component of the PAM complex, a complex required for the translocation of transit peptide-containing proteins from the inner membrane into the mitochondrial matrix in an ATP-dependent manner. Recruits mitochondrial HSP70 to drive protein translocation into the matrix using ATP as an energy source. Belongs to the Tim44 family.

kmeans	6	Green Yellow	#cbff65	3	CCDC93	9606.ENSP00000365477	Coiled-coil domain-containing protein 93; Component of the CCC complex, which is involved in the regulation of endosomal recycling of surface proteins, including integrins, signaling receptor and channels. The CCC complex associates with SNX17, retriever and WASH complexes to prevent lysosomal degradation and promote cell surface recycling of numerous cargos such as integrins ITGA5:ITGB1. Involved in copper-dependent ATP7A trafficking between the trans-Golgi network and vesicles in the cell periphery; the function is proposed to depend on its association within the CCC complex and coop [...]
kmeans	6	Green Yellow	#cbff65	3	COMMD1	9606.ENSP00000308236	COMM domain-containing protein 1; Proposed scaffold protein that is implicated in diverse physiological processes and whose function may be in part linked to its ability to regulate ubiquitination of specific cellular proteins. Can modulate activity of cullin-RING E3 ubiquitin ligase (CRL) complexes by displacing CAND1; in vitro promotes CRL E3 activity and dissociates CAND1 from CUL1 and CUL2. Promotes ubiquitination of NF-kappa-B subunit RELA and its subsequent proteasomal degradation. Down-regulates NF-kappa-B activity. Involved in the regulation of membrane expression and ubiquitin [...]
kmeans	6	Green Yellow	#cbff65	3	COMMD6	9606.ENSP00000348054	COMM domain-containing protein 6; May modulate activity of cullin-RING E3 ubiquitin ligase (CRL) complexes. Down-regulates activation of NF- kappa-B. Inhibits TNF-induced NFKB1 activation.
kmeans	7	Lime Green	#47b200	3	NAA16	9606.ENSP00000368716	N-alpha-acetyltransferase 16, NatA auxiliary subunit; Auxillary subunit of the N-terminal acetyltransferase A (NatA) complex which displays alpha (N-terminal) acetyltransferase activity.
kmeans	7	Lime Green	#47b200	3	NAA20	9606.ENSP00000335636	N-alpha-acetyltransferase 20; Catalytic subunit of the NatB complex which catalyzes acetylation of the N-terminal methionine residues of peptides beginning with Met-Asp, Met-Glu, Met-Asn and Met-Gln. Proteins with cell cycle functions are overrepresented in the pool of NatB substrates. Required for maintaining the structure and function of actomyosin fibers and for proper cellular migration; Belongs to the acetyltransferase family. ARD1 subfamily.
kmeans	7	Lime Green	#47b200	3	NAA50	9606.ENSP00000240922	N-alpha-acetyltransferase 50; N-alpha-acetyltransferase that acetylates the N-terminus of proteins that retain their initiating methionine. Has a broad substrate specificity: able to acetylate the initiator methionine of most peptides, except for those with a proline in second position. Also displays N-epsilon-acetyltransferase activity by mediating acetylation of the side chain of specific lysines on proteins. Autoacetylates in vivo. The relevance of N-epsilon-acetyltransferase activity is however unclear: able to acetylate H4 in vitro, but this result has not been confirmed in vivo. [...]

kmeans	8	Light Green	#7aff65	2	IGBP1	9606.ENSP00000363661	Immunoglobulin-binding protein 1; Associated to surface IgM-receptor; may be involved in signal transduction. Involved in regulation of the catalytic activity of the phosphatases PP2A, PP4 and PP6 by protecting their partially folded catalytic subunits from degradative polyubiquitination until they associate with regulatory subunits.
kmeans	8	Light Green	#7aff65	2	PPP2CB	9606.ENSP00000221138	Serine/threonine-protein phosphatase 2A catalytic subunit beta isoform; PP2A can modulate the activity of phosphorylase B kinase casein kinase 2, mitogen-stimulated S6 kinase, and MAP-2 kinase. Belongs to the PPP phosphatase family. PP-1 subfamily.
kmeans	9	Green 2	#00b217	2	DHX8	9606.ENSP00000262415	ATP-dependent RNA helicase DHX8; Involved in pre-mRNA splicing as component of the spliceosome. Facilitates nuclear export of spliced mRNA by releasing the RNA from the spliceosome. Belongs to the DEAD box helicase family. DEAH subfamily. DDX8/PRP22 sub-subfamily.
kmeans	9	Green 2	#00b217	2	SYF2	9606.ENSP00000236273	Pre-mRNA-splicing factor SYF2; Involved in pre-mRNA splicing as component of the spliceosome. Belongs to the SYF2 family.
kmeans	10	Green	#65ffa3	2	HAUS1	9606.ENSP00000282058	HAUS augmin-like complex subunit 1; Contributes to mitotic spindle assembly, maintenance of centrosome integrity and completion of cytokinesis as part of the HAUS augmin-like complex.
kmeans	10	Green	#65ffa3	2	HAUS8	9606.ENSP00000253669	HAUS augmin-like complex subunit 8; Contributes to mitotic spindle assembly, maintenance of centrosome integrity and completion of cytokinesis as part of the HAUS augmin-like complex.
kmeans	11	Blue	#7585ef	2	ERC1	9606.ENSP00000354158	ELKS/Rab6-interacting/CAST family member 1; Regulatory subunit of the IKK complex. Probably recruits IkappaBalpha/NFKBIA to the complex. May be involved in the organization of the cytomatrix at the nerve terminals active zone (CAZ) which regulates neurotransmitter release. May be involved in vesicle trafficking at the CAZ. May be involved in Rab-6 regulated endosomes to Golgi transport.
kmeans	11	Blue	#7585ef	2	PPFIA1	9606.ENSP00000253925	Liprin-alpha-1; May regulate the disassembly of focal adhesions. May localize receptor-like tyrosine phosphatases type 2A at specific sites on the plasma membrane, possibly regulating their interaction with the extracellular environment and their association with substrates. Belongs to the liprin family. Liprin-alpha subfamily.
kmeans	12	Medium Slate Blue	#8575ef	2	LAMTOR4	9606.ENSP00000343118	Ragulator complex protein LAMTOR4, N-terminally processed; As part of the Ragulator complex it is involved in amino acid sensing and activation of mTORC1, a signaling complex promoting cell growth in response to growth factors, energy levels, and amino acids. Activated by amino acids through a mechanism involving the lysosomal V- ATPase, the Ragulator functions as a guanine nucleotide exchange factor activating the small GTPases Rag. Activated Ragulator and Rag GTPases function as a scaffold recruiting mTORC1 to lysosomes where it is in turn activated; Belongs to the LAMTOR4 family.

kmeans	12	Medium Slate Blue	#8575ef	2	RRAGB	9606.ENSP00000262850	Ras-related GTP-binding protein B; Guanine nucleotide-binding protein that plays a crucial role in the cellular response to amino acid availability through regulation of the mTORC1 signaling cascade. Forms heterodimeric Rag complexes with RRAGC or RRAGD and cycles between an inactive GDP-bound and an active GTP-bound form. In its active form participates in the relocalization of mTORC1 to the lysosomes and its subsequent activation by the GTPase RHEB. Involved in the RCC1/Ran-GTPase pathway. Belongs to the GTR/RAG GTP-binding protein family.
kmeans	13	Medium Purple	#a675ef	2	GTF2E2	9606.ENSP00000348168	Transcription initiation factor IIE subunit beta; Recruits TFIIH to the initiation complex and stimulates the RNA polymerase II C-terminal domain kinase and DNA-dependent ATPase activities of TFIIH. Both TFIIH and TFIIE are required for promoter clearance by RNA polymerase.
kmeans	13	Medium Purple	#a675ef	2	MED21	9606.ENSP00000282892	Mediator of RNA polymerase II transcription subunit 21; Component of the Mediator complex, a coactivator involved in the regulated transcription of nearly all RNA polymerase II-dependent genes. Mediator functions as a bridge to convey information from gene- specific regulatory proteins to the basal RNA polymerase II transcription machinery. Mediator is recruited to promoters by direct interactions with regulatory proteins and serves as a scaffold for the assembly of a functional preinitiation complex with RNA polymerase II and the general transcription factors.
kmeans	14	Orchid 2	#c675ef	2	ATL1	9606.ENSP00000351155	Atlastin-1; GTPase tethering membranes through formation of trans- homooligomers and mediating homotypic fusion of endoplasmic reticulum membranes. Functions in endoplasmic reticulum tubular network biogenesis. May also regulate Golgi biogenesis. May regulate axonal development. Belongs to the TRAFAC class dynamin-like GTPase superfamily. GB1/RHD3-type GTPase family. GB1 subfamily.
kmeans	14	Orchid 2	#c675ef	2	SPAST	9606.ENSP00000320885	Spastin; ATP-dependent microtubule severing protein that specifically recognizes and cuts microtubules that are polyglutamylated. Preferentially recognizes and acts on microtubules decorated with short polyglutamate tails: severing activity increases as the number of glutamates per tubulin rises from one to eight, but decreases beyond this glutamylation threshold. Severing activity is not dependent on tubulin acetylation or detyrosination. Microtubule severing promotes reorganization of cellular microtubule arrays and the release of microtubules from the centrosome following nucleation [...]

kmeans	15	Purple	#e775ef	2	CBL	9606.ENSP00000264033	E3 ubiquitin-protein ligase CBL; Adapter protein that functions as a negative regulator of many signaling pathways that are triggered by activation of cell surface receptors. Acts as an E3 ubiquitin-protein ligase, which accepts ubiquitin from specific E2 ubiquitin-conjugating enzymes, and then transfers it to substrates promoting their degradation by the proteasome. Recognizes activated receptor tyrosine kinases, including KIT, FLT1, FGFR1, FGFR2, PDGFRA, PDGFRB, EGFR, CSF1R, EPHA8 and KDR and terminates signaling. Recognizes membrane-bound HCK, SRC and other kinases of the SRC family [...]
kmeans	15	Purple	#e775ef	2	CD2AP	9606.ENSP00000352264	CD2-associated protein; Seems to act as an adapter protein between membrane proteins and the actin cytoskeleton. In collaboration with CBLC, modulates the rate of RET turnover and may act as regulatory checkpoint that limits the potency of GDNF on neuronal survival. Controls CBLC function, converting it from an inhibitor to a promoter of RET degradation (By similarity). May play a role in receptor clustering and cytoskeletal polarity in the junction between T-cell and antigen-presenting cell (By similarity). May anchor the podocyte slit diaphragm to the actin cytoskeleton in renal glom [...]
kmeans	16	Orchid	#ef75d7	2	BRD4	9606.ENSP00000263377	Bromodomain-containing protein 4; Chromatin reader protein that recognizes and binds acetylated histones and plays a key role in transmission of epigenetic memory across cell divisions and transcription regulation. Remains associated with acetylated chromatin throughout the entire cell cycle and provides epigenetic memory for postmitotic G1 gene transcription by preserving acetylated chromatin status and maintaining high-order chromatin structure. During interphase, plays a key role in regulating the transcription of signal-inducible genes by associating with the P-TEFb complex and re [...]
kmeans	16	Orchid	#ef75d7	2	CDK9	9606.ENSP00000362361	Cyclin-dependent kinase 9; Protein kinase involved in the regulation of transcription. Member of the cyclin-dependent kinase pair (CDK9/cyclin-T) complex, also called positive transcription elongation factor b (P-TEFb), which facilitates the transition from abortive to productive elongation by phosphorylating the CTD (C-terminal domain) of the large subunit of RNA polymerase II (RNAP II) POLR2A, SUPT5H and RDBP. This complex is inactive when in the 7SK snRNP complex form. Phosphorylates EP300, MYOD1, RPB1/POLR2A and AR and the negative elongation factors DSIF and NELF. Regulates cytoki [...]

kmeans	17	Hot Pink	#ef75b6	2	ERO1A	9606.ENSP00000379042	ERO1-like protein alpha; Oxidoreductase involved in disulfide bond formation in the endoplasmic reticulum. Efficiently reoxidizes P4HB/PDI, the enzyme catalyzing protein disulfide formation, in order to allow P4HB to sustain additional rounds of disulfide formation. Following P4HB reoxidation, passes its electrons to molecular oxygen via FAD, leading to the production of reactive oxygen species (ROS) in the cell. Required for the proper folding of immunoglobulins. Involved in the release of the unfolded cholera toxin from reduced P4HB/PDI in case of infection by V.cholerae, thereby pla [...]
kmeans	17	Hot Pink	#ef75b6	2	ERP44	9606.ENSP00000262455	Endoplasmic reticulum resident protein 44; Mediates thiol-dependent retention in the early secretory pathway, forming mixed disulfides with substrate proteins through its conserved CRFS motif. Inhibits the calcium channel activity of ITPR1. May have a role in the control of oxidative protein folding in the endoplasmic reticulum. Required to retain ERO1A and ERO1B in the endoplasmic reticulum.
kmeans	18	Pink	#ef7595	2	DHX37	9606.ENSP00000311135	Probable ATP-dependent RNA helicase DHX37; DEAH-box helicase 37.
kmeans	18	Pink	#ef7595	2	UTP25	9606.ENSP00000419005	Digestive organ expansion factor homolog; Regulates the p53 pathway to control the expansion growth of digestive organs.

Supplementary Table S4: The table presents clusters of downregulated proteins (1 mM citicoline vs. untreated cells, 6h), which were identified through PPI analysis in STRING (refer to Supplementary Table 1). Clustering was performed by applying a stringent connectivity strength threshold of 0.9 and utilizing the knn algorithm. For each cluster, the table indicates the clustering method, a unique cluster number (consistent with the numbering in the Gene Ontology figures across the manuscript), the assigned cluster color (corresponding to the colors used in the GO charts), the protein name, protein identifier, and protein description.

Accession	Description	LogFC	p.mod	q.mod
**Q4G176**	Malonate--CoA ligase ACSF3, mitochondrial [OS=Homo sapiens]	1,682667	0,000727	0,537251
**Q9UJX4**	Anaphase-promoting complex subunit 5 [OS=Homo sapiens]	0,739955	0,001931	0,543858
**Q9H490**	Phosphatidylinositol glycan anchor biosynthesis class U protein [OS=Homo sapiens]	1,169372	0,00223	0,543858
**Q9HAU5**	Regulator of nonsense transcripts 2 [OS=Homo sapiens]	0,649776	0,002382	0,543858
**Q12986**	Transcriptional repressor NF-X1 [OS=Homo sapiens]	4,319418	0,002533	0,543858
**Q96RE7**	Nucleus accumbens-associated protein 1 [OS=Homo sapiens]	0,631962	0,0031	0,543858
**Q96RQ3**	Methylcrotonoyl-CoA carboxylase subunit alpha, mitochondrial [OS=Homo sapiens]	3,215884	0,003207	0,543858
**Q17RY0**	Cytoplasmic polyadenylation element-binding protein 4 [OS=Homo sapiens]	0,627651	0,005322	0,543858
**Q5EBL4**	RILP-like protein 1 [OS=Homo sapiens]	1,871499	0,005398	0,543858
**O00584**	Ribonuclease T2 [OS=Homo sapiens]	1,152717	0,005712	0,543858
**Q9NW82**	WD repeat-containing protein 70 [OS=Homo sapiens]	0,68384	0,006414	0,543858
**Q86UV5**	Ubiquitin carboxyl-terminal hydrolase 48 [OS=Homo sapiens]	0,678052	0,006889	0,543858
**P17612**	cAMP-dependent protein kinase catalytic subunit alpha [OS=Homo sapiens]	0,950672	0,007164	0,543858
**Q9NP50**	SIN3-HDAC complex-associated factor [OS=Homo sapiens]	1,716581	0,007253	0,543858
**Q08AM6**	Protein VAC14 homolog [OS=Homo sapiens]	0,632033	0,008241	0,543858
**P10253**	Lysosomal alpha-glucosidase [OS=Homo sapiens]	1,202341	0,010251	0,543858
**Q99519**	Sialidase-1 [OS=Homo sapiens]	0,807086	0,01077	0,543858
**Q9P0J7**	E3 ubiquitin-protein ligase KCMF1 [OS=Homo sapiens]	2,799182	0,011745	0,543858
**Q08AD1**	Calmodulin-regulated spectrin-associated protein 2 [OS=Homo sapiens]	0,746292	0,01397	0,543858
**Q8IXM3**	Large ribosomal subunit protein mL41 [OS=Homo sapiens]	1,74957	0,016152	0,543858
**Q9NYP9**	Protein Mis18-alpha [OS=Homo sapiens]	1,212715	0,016377	0,543858
**Q9BWW4**	Single-stranded DNA-binding protein 3 [OS=Homo sapiens]	1,002599	0,01705	0,543858
**Q6AI08**	HEAT repeat-containing protein 6 [OS=Homo sapiens]	1,8591	0,017136	0,543858
**P18085**	ADP-ribosylation factor 4 [OS=Homo sapiens]	0,91638	0,017138	0,543858
**P49407**	Beta-arrestin-1 [OS=Homo sapiens]	0,787967	0,020491	0,543858
**Q9Y663**	Heparan sulfate glucosamine 3-O-sulfotransferase 3A1 [OS=Homo sapiens]	2,152602	0,022252	0,543858
**Q07864**	DNA polymerase epsilon catalytic subunit A [OS=Homo sapiens]	1,250414	0,022627	0,543858
**Q9BTM9**	Ubiquitin-related modifier 1 [OS=Homo sapiens]	0,617449	0,022888	0,543858
**Q8TBX8**	Phosphatidylinositol 5-phosphate 4-kinase type-2 gamma [OS=Homo sapiens]	0,658719	0,025854	0,543858
**P14373**	Zinc finger protein RFP [OS=Homo sapiens]	1,614283	0,027449	0,543858
**Q9NTM9**	Copper homeostasis protein cutC homolog [OS=Homo sapiens]	1,37275	0,028323	0,543858

**Q96GX5**	Serine/threonine-protein kinase greatwall [OS=Homo sapiens]	0,916824	0,02892	0,543858
**A8CG34**	Nuclear envelope pore membrane protein POM 121C [OS=Homo sapiens]	0,898175	0,033071	0,543858
**P42773**	Cyclin-dependent kinase 4 inhibitor C [OS=Homo sapiens]	1,055952	0,034892	0,543858
**Q6NW34**	Nucleolus and neural progenitor protein [OS=Homo sapiens]	1,808202	0,037695	0,543858
**Q5UCC4**	ER membrane protein complex subunit 10 [OS=Homo sapiens]	0,664417	0,038598	0,543858
**Q9BUM1**	Glucose-6-phosphatase 3 [OS=Homo sapiens]	1,404312	0,039811	0,543858
**Q8IWA4**	Mitofusin-1 [OS=Homo sapiens]	1,42995	0,041016	0,543858
**Q5T6V5**	Queuosine 5'-phosphate N-glycosylase/hydrolase [OS=Homo sapiens]	0,712522	0,042485	0,543858
**P56377**	AP-1 complex subunit sigma-2 [OS=Homo sapiens]	1,873425	0,044723	0,543858
**Q6GMV3**	Putative peptidyl-tRNA hydrolase PTRHD1 [OS=Homo sapiens]	2,171374	0,045903	0,543858
**Q8WTV0**	Scavenger receptor class B member 1 [OS=Homo sapiens]	1,239732	0,046171	0,543858
**Q9Y6D5**	Brefeldin A-inhibited guanine nucleotide-exchange protein 2 [OS=Homo sapiens]	2,006161	0,048327	0,543858

Supplementary Table S5: Reported in this table are the proteins upregulated by 1 mM citicoline (compared to untreated cells) after 18 h of stimulated, with data filtered for *p.mod* ≤ 0.01. For each entry, the Uniprot Accession number, protein description, log_2FC, p.mod and FDR (*q.mod*) from the Limma test are provided.

Accession	Description	LogFC	p.mod	q.mod
**Q9H2F5**	Enhancer of polycomb homolog 1 [OS=Homo sapiens]	-4,16023	2,67E-06	0,00791
**Q9P2Y4**	Zinc finger protein 219 [OS=Homo sapiens]	-5,93805	4,34E-05	0,064212
**P82914**	Small ribosomal subunit protein uS15m [OS=Homo sapiens]	-4,1268	0,000469	0,462175
**Q7Z5K2**	Wings apart-like protein homolog [OS=Homo sapiens]	-0,66219	0,001003	0,543858
**Q8WUY1**	Protein THEM6 [OS=Homo sapiens]	-1,29997	0,001501	0,543858
**Q8N5F7**	NF-kappa-B-activating protein [OS=Homo sapiens]	-0,82716	0,002192	0,543858
**P56181-2**	Isoform 2 of NADH dehydrogenase [ubiquinone] flavoprotein 3, mitochondrial [OS=Homo sapiens]	-0,75638	0,002768	0,543858
**Q4LE39**	AT-rich interactive domain-containing protein 4B [OS=Homo sapiens]	-0,90944	0,00331	0,543858
**Q969G6**	Riboflavin kinase [OS=Homo sapiens]	-1,56398	0,003787	0,543858
**Q9Y2R4**	Probable ATP-dependent RNA helicase DDX52 [OS=Homo sapiens]	-0,60583	0,004885	0,543858
**Q0VDG4**	Secernin-3 [OS=Homo sapiens]	-0,62058	0,008808	0,543858
**Q9NQ48**	Leucine zipper transcription factor-like protein 1 [OS=Homo sapiens]	-0,60742	0,010761	0,543858
**Q13686**	Nucleic acid dioxygenase ALKBH1 [OS=Homo sapiens]	-2,86923	0,010807	0,543858
**Q96CB8**	Integrator complex subunit 12 [OS=Homo sapiens]	-0,62201	0,010813	0,543858
**Q13459**	Unconventional myosin-IXb [OS=Homo sapiens]	-3,81492	0,010832	0,543858
**Q3V6T2**	Girdin [OS=Homo sapiens]	-0,59716	0,011795	0,543858
**Q9BV29**	Coiled-coil domain-containing protein 32 [OS=Homo sapiens]	-0,92047	0,012383	0,543858
**Q9P2B4**	CTTNBP2 N-terminal-like protein [OS=Homo sapiens]	-1,02315	0,013518	0,543858
**Q5VW36**	Focadhesin [OS=Homo sapiens]	-1,62077	0,014997	0,543858
**P22466**	Galanin peptides [OS=Homo sapiens]	-1,32467	0,015088	0,543858
**Q8IY37**	Probable ATP-dependent RNA helicase DHX37 [OS=Homo sapiens]	-1,16002	0,017259	0,543858
**Q9HCD6**	Protein TANC2 [OS=Homo sapiens]	-2,02023	0,01831	0,543858
**Q86TB9**	Protein PAT1 homolog 1 [OS=Homo sapiens]	-2,12621	0,018374	0,543858
**Q15043**	Metal cation symporter ZIP14 [OS=Homo sapiens]	-2,06126	0,018735	0,543858
**Q5TEC6**	Histone H3-7 [OS=Homo sapiens]	-1,71593	0,019474	0,543858
**Q15398-1**	Isoform 2 of Disks large-associated protein 5 [OS=Homo sapiens]	-0,84875	0,019649	0,543858
**Q8WUF5**	RelA-associated inhibitor [OS=Homo sapiens]	-2,15368	0,020143	0,543858
**Q02241**	Kinesin-like protein KIF23 [OS=Homo sapiens]	-1,32593	0,021186	0,543858
**Q13043**	Serine/threonine-protein kinase 4 [OS=Homo sapiens]	-0,59079	0,021471	0,543858

**O15091**	Mitochondrial ribonuclease P catalytic subunit [OS=Homo sapiens]	-1,22131	0,023348	0,543858
**Q8N122**	Regulatory-associated protein of mTOR [OS=Homo sapiens]	-0,58775	0,023449	0,543858
**Q96GX2**	Ataxin-7-like protein 3B [OS=Homo sapiens]	-1,04606	0,024973	0,543858
**Q9Y2E8**	Sodium/hydrogen exchanger 8 [OS=Homo sapiens]	-0,65618	0,026113	0,543858
**Q13158**	FAS-associated death domain protein [OS=Homo sapiens]	-0,93822	0,026825	0,543858
**Q6ZVC0**	Neuronal tyrosine-phosphorylated phosphoinositide-3-kinase adapter 1 [OS=Homo sapiens]	-1,63789	0,031802	0,543858
**Q9HCU9**	Breast cancer metastasis-suppressor 1 [OS=Homo sapiens]	-0,58636	0,032015	0,543858
**P30414**	NK-tumor recognition protein [OS=Homo sapiens]	-1,36598	0,035099	0,543858
**Q3B726**	DNA-directed RNA polymerase I subunit RPA43 [OS=Homo sapiens]	-2,34447	0,03597	0,543858
**Q92995**	Ubiquitin carboxyl-terminal hydrolase 13 [OS=Homo sapiens]	-0,8517	0,038369	0,543858
**Q96JP5**	E3 ubiquitin-protein ligase ZFP91 [OS=Homo sapiens]	-1,28984	0,038788	0,543858
**Q9UQC2**	GRB2-associated-binding protein 2 [OS=Homo sapiens]	-0,66663	0,04337	0,543858
**P43694**	Transcription factor GATA-4 [OS=Homo sapiens]	-1,97798	0,04399	0,543858
**Q7Z4G1**	COMM domain-containing protein 6 [OS=Homo sapiens]	-1,01251	0,044151	0,543858
**Q6UXV4**	MICOS complex subunit MIC27 [OS=Homo sapiens]	-1,12219	0,045371	0,543858
**Q5T0F9-2**	Isoform 2 of Coiled-coil and C2 domain-containing protein 1B [OS=Homo sapiens]	-3,68121	0,046906	0,543858
**O14920**	Inhibitor of nuclear factor kappa-B kinase subunit beta [OS=Homo sapiens]	-3,0762	0,047092	0,543858
**Q7LGA3**	Heparan sulfate 2-O-sulfotransferase 1 [OS=Homo sapiens]	-1,34809	0,047209	0,543858
**Q86VQ1**	Glucocorticoid-induced transcript 1 protein [OS=Homo sapiens]	-0,67567	0,047756	0,543858
**Q9NYF8-3**	Isoform 3 of Bcl-2-associated transcription factor 1 [OS=Homo sapiens]	-2,0921	0,049176	0,543858

Supplementary Table S6: Reported in this table are the proteins downregulated by 1 mM citicoline (compared to untreated cells) after 18 h of stimulation, with data filtered for *p.mod* ≤ 0.01. For each entry, the Uniprot Accession number, protein description, log_2FC, p.mod and FDR (*q.mod*) from the Limma test are provided.
